# Uncovering the Relationship between Sulphation Patterns and Conformation of Iduronic Acid in Heparan Sulphate

**DOI:** 10.1038/srep29602

**Published:** 2016-07-14

**Authors:** Po-Hung Hsieh, David F. Thieker, Marco Guerrini, Robert J. Woods, Jian Liu

**Affiliations:** 1 Division of Chemical Biology and Medicinal Chemistry, Eshelman School of Pharmacy, University of North Carolina, Chapel Hill, North Carolina 27599, USA; 2 Complex Carbohydrate Research Center, University of Georgia, Athens, Georgia 30602, USA; 3 Istituto di Ricerche Chimiche e Biochimiche ‘G. Ronzoni’, via G. Colombo 81, 20133 Milan, Italy

## Abstract

The L-iduronic acid (IdoA) residue is a critically important structural component in heparan sulphate polysaccharide for the biological functions. The pyranose ring of IdoA is present in ^*1*^*C*_*4*_-chair, ^*2*^*S*_*O*_-skew boat, and less frequently, in ^*4*^*C*_*1*_-chair conformations. Here, we analyzed the conformation of IdoA residue in eight hexasaccharides by NMR. The data demonstrate a correlation between the conformation of IdoA and sulphations in the surrounding saccharide residues. For the 2-*O*-sulpho IdoA residue, a high degree of sulphation on neighboring residues drives ring dynamics towards the ^*2*^*S*_*O*_-skew boat conformer. In contrast, the nonsulphated IdoA residue is pushed towards the ^*1*^*C*_*4*_-chair conformer when the neighboring residues are highly sulphated. Our data suggest that the conformation of IdoA is regulated by the sulphation pattern of nearby saccharides that is genetically controlled by the heparan sulphate biosynthetic pathway.

Heparan sulphate (HS) is a polysaccharide that consists of disaccharide repeating units of a glucuronic acid (GlcA) or iduronic acid (IdoA) residue linked to a glucosamine (GlcN) residue. Capable of carrying sulpho groups by IdoA, GlcA and GlcN residues, HS is a highly negatively charged macromolecule under physiological conditions. HS participates in numerous physiological and pathophysiological processes, such as embryonic development, inflammatory responses, blood coagulation and viral/bacterial infections^1^,^2^,^3^. Most notably, heparin, a special form of HS containing higher sulphation and IdoA levels, is a commonly used anticoagulant drug in clinics for treatment of patients with thrombotic disorders^4^.

The molecular mechanism that allows HS to interact with specific protein targets remains elusive. The existing evidence suggests that the binding selectivity of HS to different proteins is dominated by sulphation patterns^5^. The types of sulphation found in HS include the 2-OH of IdoA and GlcA residue, as well as the *N*-, 3-OH and 6-OH position of GlcN residues. The negatively charges from the sulpho groups interact with the positively charged amino acid residues from proteins. Moreover, the pyranose rings of 2-*O*-sulpho iduronic acid (IdoA2S) and IdoA residues adopt both chair (^*1*^*C*_*4*_) and skew boat (^*2*^*S*_*O*_) conformations. The pyranose rings of GlcA and GlcN are, however, predominantly present in ^*4*^*C*_*1*_ conformation^6^,^7^. The conformational flexibility of IdoA2S residues is known to be essential for the binding of HS to antithrombin to display anticoagulant activity^8^ and fibroblast growth factors to regulate cell growth^9^.

Whether sulphations of neighboring residues affects the ring conformation of IdoA has not been systematically examined. The previous conformational analysis of IdoA or IdoA2S residues were carried out using sporadic structures of heparin or HS oligosaccharides either isolated from depolymerized heparin or through chemical synthesis^10^,^11^,^12^,^13^,^14^,^15^,^16^. The only available systematic study was to limited to the combined effect of *N*-sulphation and 6-*O*-sulphation in synthetic trisaccharides^17^,^18^. The authors found that 6-*O*-sulphation drives the conformation equilibrium to ^*2*^*S*_*O*_ for IdoA2S, but the study did not offer the information on the effects of 2-*O*- and 3-*O*-sulphations. In the present study, we demonstrate that the conformation of IdoA is influenced by combined effects from three sulphation types, including 2-*O*-, 3-*O*- and 6-*O*-sulphation using structurally defined model hexasaccharides. Our findings suggest a cellular mechanism to control the conformation of IdoA through the biosynthetic pathway, offering a new hypothesis for the genetic regulation of conformation of carbohydrates.

## Results

**Figure 1:**
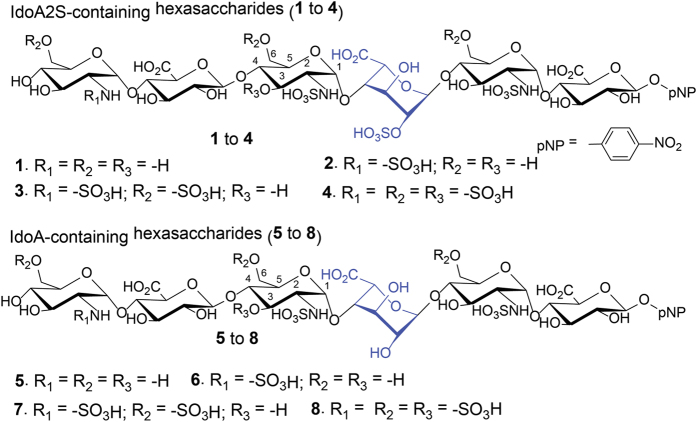
Chemical structures of hexasaccharides used in this study. Structures of hexasaccharides synthesized in this study. Both IdoA2S (in **1** to **4**) and IdoA (**5** to **8**) residues are colored in blue.

Eight different hexasaccharides (Fig. 1) were synthesized using the chemoenzymatic method^19^,^20^. Hexasaccharides (**1**–**4**) contained an IdoA2S residue that is flanked by two saccharides on the reducing end and three residues at the nonreducing end with different degrees of sulphations. The sulphation types cover *N*-unsubstituted GlcN (**1**), *N*-sulphation (**2**), 6-*O*-sulphation (**3**) and 3-*O*-sulphation (**4**) (Fig. 1). Hexasaccharides (**5**–**8**) are nearly carbon copies of **1**–**4** with the exception that an IdoA residue was introduced to substitute the IdoA2S residue (Fig. 1). It should be noted that the syntheses of IdoA-containing hexasaccharides (**5** to **8**) were not previously reported using the chemoenzymatic method. The purity analysis and molecular mass determination was completed by anion exchange HPLC and electrospray ionization mass spectrometry (Supplementary Figs 1 and 2). The structures of hexasaccharide were also confirmed by ^1^H-NMR and ^13^C-NMR (Supplementary Figs 3–58). The NMR signals were fully assigned (Supplementary Table 1 and 2).

**Figure 2:**
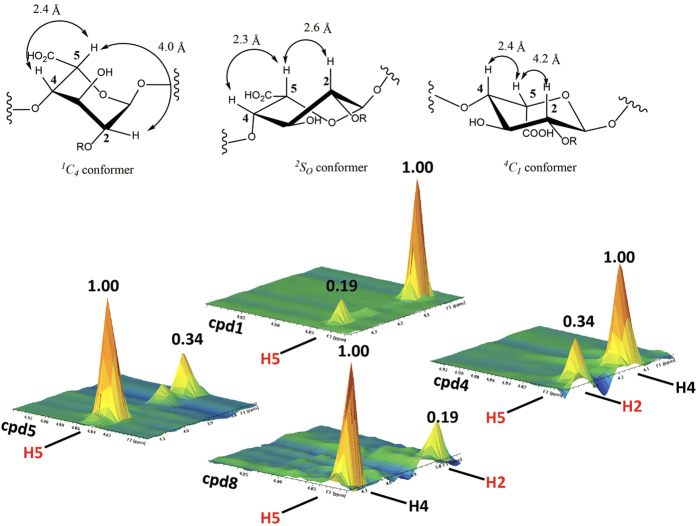
3-D Images of the NOE cross-peaks of H2-H5 and H4-H5 signals from representative hexasaccharides. The images of NOE cross-peak signals of H2-H5 and H4-H5 from compound **1**, **4**, **5** and **8** are presented. Cpd **1** and **4** are IdoA2S-containing hexasaccharides, and cpd **5** and cpd **8** are IdoA-containing hexasaccharides. The structures of three different conformers for IdoA or IdoA2S are presented on top of the figure, where R represents –H or –SO_3_H. The calculated distance between H2 and H5, as well as H4 and H5 and displayed in lines with doubled arrows.

Both IdoA and IdoA2S residues interconvert between different conformations, including ^*1*^*C*_*4*_, ^*2*^*S*_*O*_^21^ and ^*4*^*C*_*1*_ (Fig. 2)^22^ Each conformer has a unique set of three bond proton-proton coupling constants (^*3*^*J*_*H-H*_)^23^,^24^, which were obtained for the IdoA2S and IdoA residues in the eight hexasaccharides (Table 1). The relative populations of IdoA/IdoA2S ring conformations for the eight compounds were generated by minimizing the residual sum of squares (RSS) between the observed NMR *J*-values and the total contribution back-calculated for at least 350,000 snapshots of each structure from the molecular dynamics (MD). Due to the slow rate of ring flipping for IdoA (μs timescale)^25^, three independent simulations were performed for **1**–**8** (1 μs each), in which the IdoA/IdoA2S ring was restrained in each of the three conformations. As shown in Table 1, the *J*-value RSS for each oligosaccharide was less than ~1.0 Hz, which compares favorably with the deviations in *J*-values reported recently in a study that employed single energy-minimized conformations of a related HS fragment^26^.

A correlation between the sulphation degree and the population of ^*2*^*S*_*O*_ conformer was observed. For IdoA2S, the ring with lower sulphation levels (**1** and **2)** predominantly populated the ^*1*^*C*_*4*_ conformation (~68%). As the level of sulphation increased by adding 6-*O*-sulphation (hexasaccharide **3**) and 3-*O*-sulphation (hexasaccharide **4**), the dominant conformation for IdoA2S was shifted to the ^*2*^*S*_*O*_ conformer. In comparing the percentage of ^*2*^*S*_*O*_ in **3** and **2**, it appears that 6-*O*-sulphation drives the IdoA2S residue towards ^*2*^*S*_*O*_ conformation, confirming the results by Munoz-Garcia and colleagues using trisaccharide model compounds^17^. In addition, the ^*2*^*S*_*O*_ conformer was populated as high as 75% in hexasaccharide **4**, the most highly sulphated hexasaccharide among the tested compounds (Table 1). The ^*2*^*S*_*O*_ conformer population for **4** is similar to that recently reported for a closely-related pentasaccharide of HS^26^. Together, the data suggests that neighboring sulphation upregulates the ^*2*^*S*_*O*_ conformer for an IdoA2S residue.

For the IdoA-containing hexasaccharides (**5**–**8**), the sulphation impact on the ^*2*^*S*_*O*_ conformer for IdoA residues exhibited a different trend from that was observed for an IdoA2S residue. Here, the IdoA residue displayed 47% and 49% of ^*2*^*S*_*O*_ conformer in compounds **5** and **6**, the low sulphated hexasaccharides (Table 1). As the sulphation level increased, the population of ^*2*^*S*_*O*_ diminished to 29% in hexasaccharide **7** and 19% in hexasaccharide **8**, respectively (Table 1). In addition, a small percentage of ^*4*^*C*_*1*_ conformer was detected in **7** and **8**. This low abundance of the ^*4*^*C*_*1*_ conformer is in agreement with previous studies^11^,^21^. Sulphation of the *N*-unsubstituted GlcN residue at the nonreducing end of **1** and **5** had no effects on the conformer distribution for IdoA2S and IdoA residue. This observation is not surprising considering that the GlcN residue is three saccharide residues away from the IdoA2S and IdoA residue.

The distributions of conformers were further confirmed by ^1^H-^1^H NOE analysis. This analysis is uniquely sensitive to detect the presence of the ^*2*^*S*_*O*_ conformer due to the difference in the atomic distance of H2 and H5 in the ^*2*^*S*_*O*_ conformer (2.6 Å), compared with the ^*1*^*C*_*4*_ and ^*4*^*C*_*1*_ conformer (4.0 Å and 4.2 Å, respectively) (Fig. 2)^27^ The shorter atomic distance of H2 and H5 in the ^*2*^*S*_*O*_ conformer offers a stronger intensity for NOE signal. Since heparin oligosaccharides may exhibit anisotropic motion in solution^28^, inter-proton distances cannot be calculated using the two-spin approximation, and a more extended experimental procedure, such as quantifying the anisotropy by relaxation or by off-resonance ROESY measurements^27^, would be necessary. However, together with the analysis of ^3^*J*_H-H_ couplings, the analysis of H2-H5/H4-H5 NOE ratio can be used to confirm the conformer population of IdoA2S and IdoA residues. To compare the relative intensities of the H2-H5 NOE among hexasaccharides, a ratio of the volume of H2-H5 cross peak and that of H4-H5 was obtained (Table 1 and Fig. 2). The relative intensity of H2-H5 NOE signal from the IdoA2S residue in **4** was 0.34, while this signal in **1** was only 0.19, suggesting that the IdoA2S residue in **4** displayed higher population of ^*2*^*S*_*O*_ than that in **1** (Fig. 2). For IdoA residue in hexasaccharide **5** and **8**, the relative intensity of H2-H5 NOE signal from IdoA residue in **8** was 0.19, and this signal in **5** was 0.34, suggesting that the IdoA residue in **8** displayed lower population of ^*2*^*S*_*O*_ than that in **5** (Fig. 2). The intensity of H4-H5 cross-peak of NOE signals should remain unchanged as the atomic distance of H4-H5 is similar amongst the different conformers (Fig. 2). The ratio of H2-H5/H4-H5 NOE signals among **1**–**4** exhibited an increasing trend as the level of sulphation elevated (Table 1), consistent with the conclusion from the J analysis. In the IdoA-containing series of hexasaccharides (**5**–**8**), the NOESY analysis indicated a decreasing trend in the ratio of H2-H5/H4-H5 NOE signals, suggesting that the ^*2*^*S*_*O*_ conformer becomes less abundant as the surrounding sulphation level increased.

**Figure 3:**
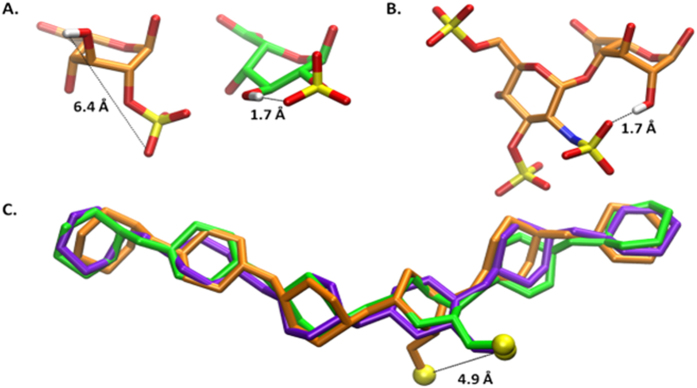
Images of IdoA2S or IdoA and hexasaccharides from the MD simulations. Atomic distances are indicated by dashed lines. The ^*1*^*C*_*4*_, ^*2*^*S*_*O*_, and ^*4*^*C*_*1*_ conformations of IdoA2S are colored orange, green, and purple, respectively. Panel A shows the IdoA2S residue in the ^*1*^*C*_*4*_ and ^*2*^*S*_*O*_ conformations (from a representative snapshot of the simulations of 4). Panel B displays the GlcNS(3S,6S) and IdoA residues from a representative frame of the simulation of 8 in the ^*2*^*S*_*O*_ conformation. Panel C contains a superimposition of the ring atoms in oligosaccharides from simulations of 4 in each of the three IdoA ring conformations. The sulfur atom of the IdoA2S residue is also displayed in yellow for each conformation. The RMSD of the ring atoms of the ligands with IdoA in the ^*1*^*C*_*4*_ and ^*4*^*C*_*1*_ conformation are 0.9 Å and 0.7 Å, respectively, relative to the molecule containing IdoA in the ^*2*^*S*_*O*_ conformation. Each model is the snapshot most similar to the average structure during the MD simulations.

The observed conformational preferences can be explained in part by an examination of the hydrogen bonds detected during molecular dynamics (MD) simulations of the oligosaccharides (Supplementary Table 3). For example, an intra-residue hydrogen bond between the 2-*O*-sulpho and the 3-OH group in IdoA2S stabilizes the ^*2*^*S*_*o*_ conformation; however, the distance between these two groups is prohibitive for hydrogen bond formation in the ^*1*^*C*_*4*_ conformation (Fig. 3A and Supplementary Video 1), potentially leading to the preference for the former conformation. Apparently the presence of this hydrogen bond in the ^*4*^*C*_*1*_ conformation provides insufficient stabilization to result in appreciable population of this higher energy form^23^. The absence of sulphation at the 2-position in compounds **5**–**8** prevents the described intra-residue hydrogen bond from forming. However, in IdoA-containing hexasaccharides, the 2-OH group can form a hydrogen bond with the *N*-sulpho moiety in the adjacent residue on the nonreducing end, but only when the IdoA is in the ^*1*^*C*_*4*_ conformation (Fig. 3B), potentially leading to the preference for this conformation. While the overall conformation of the oligosaccharides is relatively insensitive to the conformation of the IdoA ring under the conditions of MD simulations^29^, the position of the sulpho group in IdoA2S is markedly impacted by ring conformation, differing by 4.9 Å between the ^*2*^*S*_*o*_ and ^*1*^*C*_*4*_ structures (Fig. 3C).

**Figure 4:**
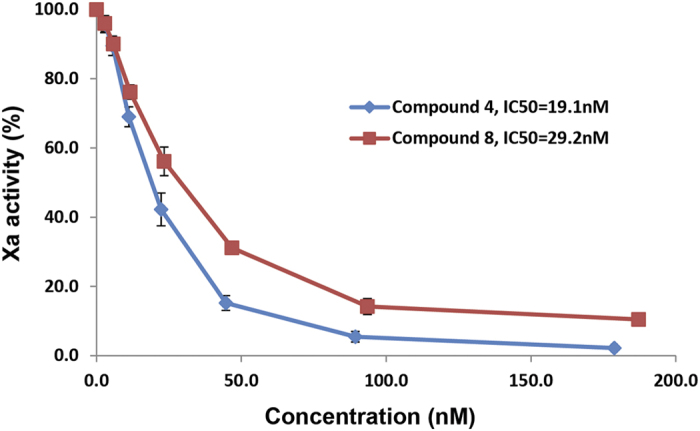
Inhibition curve of Xa activity by compound 4 and 8. Different concentrations of compound **4** and **8** were incubated with antithrombin and Xa. The activity of Xa was then measured using a chromogenic substrate.

The biological outcome was analyzed using the antithrombin-HS binding model. Antithrombin deactivates coagulating factor Xa upon the binding to HS. Among the eight synthesized hexasaccharides, only **4** and **8** produce anticoagulant activity due to the presence of 3-*O*-sulphation^30^,^31^. The binding affinity (K_d_) of **4** and **8** to antithrombin was determined to be 15.5 ± 3.4 nM, and was 32.6 ± 5.3 nM, respectively. The IC_50_ value for the inhibition of the activity of Xa, a measurement for the anticoagulant activity, was determined to be 19 nM and 29 nM for **4** and **8**, respectively (Fig. 4). A small, but notable, difference in the binding affinity to antithrombin and potency for the anti-Xa activity between **4** and **8** was observed. The co-crystal structure analysis of antithrombin and a pentasaccharide, structurally very similar to **4**, revealed no amino acid residues from antithrombin directly interacted with the 2-*O*-sulpho group from the IdoA2S residue in the pentasaccharide^32^,^33^, suggesting that a decrease in binding affinity from **8** was not due to the lack of 2-*O*-sulpho group. The IdoA2S residue is known to display the ^*2*^*S*_*O*_ conformer when it interacts with antithrombin in the co-crystal structure^32^, the solution complex^34^, as well as from study of the interactions of antithrombin with a conformationally-fixed oligosaccharide^8^. Gurerrini *et al*. demonstrated that the presence of unnatural saccharide residues at the reducing end of the IdoA2S residue domain reduces the binding affinity to antithrombin and decreases the anti-Xa activity. Authors attributed to the decreased effects to lower population of the ^*2*^*S*_*O*_ conformer of the IdoA2S residue^35^. Although the difference is small in our study, it is tempting to attribute the higher binding affinity to antithrombin and stronger anti-Xa activity of **4** to the increased ^*2*^*S*_*O*_ conformer population.

## Discussion

Sulphation plays a critical role in determining the biological functions of HS. The interactions between HS and proteins are primarily mediated by the negative charges from the sulpho groups and the positive charges from lysine and arginine residues from the proteins as well as some hydrogen bonding interactions^36^,^37^. Here, we demonstrate that sulphation also affects the dynamics of the ring conformation of IdoA and IdoA2S. Interestingly, the sulphation of neighboring residues impacts the conformer population differently depending on whether the IdoA carries a 2-*O*-sulpho group. Our study relies on two crucial pieces of innovation that were unavailable to previous studies: the ability to design and synthesize HS oligosaccharides and a thorough computational analysis of these molecules. A uniquely designed group of eight hexasaccharides allowed us to investigate the effects of 2-*O*-sulphation, 6-*O*-sulphation and 3-*O*-sulphation on the conformation of IdoA. The oligosaccharides were synthesized by the chemoenzymatic method that offers an efficient synthesis of HS oligosaccharides^19^,^20^. It would be very difficult to obtain a similar set of oligosaccharides by isolation or the chemical synthetic approach. Improvements in molecular dynamics allowed us to extend the simulation from the traditional nanosecond timescale to a full microsecond for each compound. Considering the slow rate of conversion between conformations for IdoA^29^, simulations of the compounds were performed for each conformation (24 in total). As a result, the proton-proton coupling constants (^*3*^*J*_*H-H*_) obtained from NMR are close to the simulated values (Table 1). Furthermore, extending the length of the simulations allowed us to identify key intra-molecular interactions that stabilize the ring conformations.

**Figure 5:**
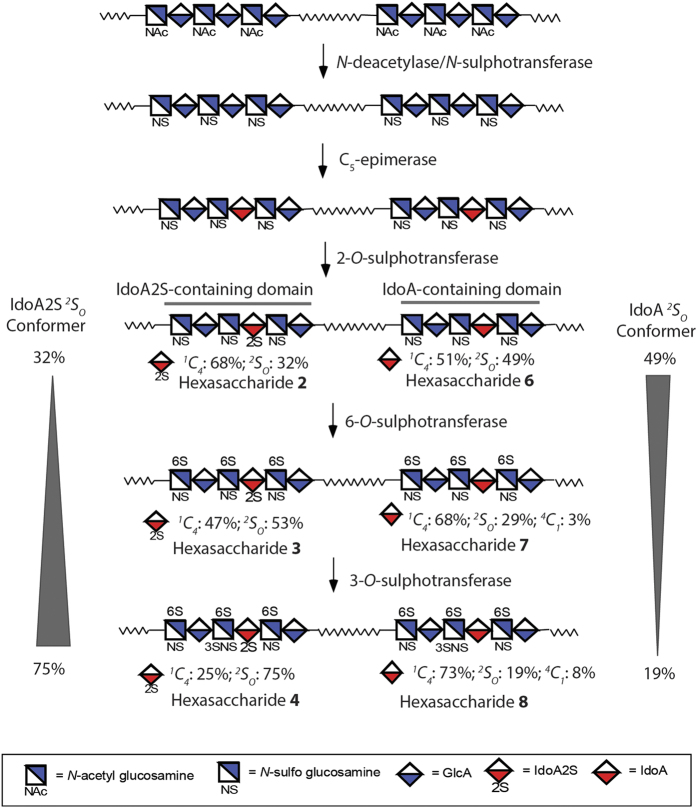
Biosynthetic pathway of HS and relevant to the hexasaccharide model compounds. HS biosynthesis is initiated from the polysaccharide containing -GlcA-GlcNAc- repeating units, followed by the modifications of *N*-deacetylase/*N*-sulphotransferase, C_5_-epimerase, 2-*O*-sulphotransferase, 6-*O*-sulphotransferase and 3-*O*-sulphotransferase. The 2-*O*-sulphotransferase modification can be incomplete, resulting the polysaccharide product consists of IdoA2S-containing domain and IdoA-containing domain. Hexasaccharide **2** and **6** represent these two domain structures. Hexasaccharide **3** and **7** represent the domain structures after 6-*O*-sulphotransferase modification, and hexasaccharide **4** and **8** represent the structures after 3-*O*-sulphotransferase modification. The wavy lines represent the linker between domains in HS polysaccharide with unknown structures.

This investigation offers insight into the control of the ring conformation of IdoA through the HS biosynthetic pathway. The pathway involves a series of sulphotransferases and a specialized C_5_-epimerase (Fig. 5). After the predominantly ^*4*^*C*_*1*_ GlcA residue is epimerized to IdoA, the HS sequence diverges into two separate pathways to form both IdoA2S-containing and IdoA-containing domains. Both paths are then subjected to 6-*O*-sulphotransferase and 3-*O*-sulphotransferase modifications to form matured HS polysaccharides. For the IdoA2S-containing domains, additional sulphation modifications, *i.e*. 6-*O*-sulphation and 3-*O*-sulphation, increase the ^*2*^*S*_*O*_ conformer population, whereas for the IdoA-containing domains, additional sulphation modification decreases the ^*2*^*S*_*O*_ conformer population. Given that IdoA and IdoA2S residues both exist in HS^38^, balancing the distribution of different conformers may represent an important structural feature for shaping the selectivity of HS towards potential binding partners. It has been well established that the amino acid sequences determine the secondary and tertiary structures of proteins. The availability of structurally defined oligosaccharides coupled with sophisticated NMR and computational techniques will open the door to unveil the structure of HS chain beyond the primary saccharide sequence.

## Methods

### Synthesis of compound **1** to **8**

The synthesis of **1** to **8** was completed according to the chemoenzymatic method published previously^6^,^19^. Briefly, heparosan synthase-2 (PmHS2) from *Pasteurella multocida* to elongate the monosaccharide, *p*-nitrophenyl glucuronide (GlcA-pnp), to a hexasaccharide backbone. The hexasaccharide was then subjected to the modification of *N*-sulphotransferase, C_5_-epimerase, 6-*O*-sulphotransferase, and 3-*O*-sulphotransferase to generate IdoA residues and introduce the sulpho group at the desired position to produce the final products. For the synthesis of IdoA2S-containing hexasaccharides (**1** to **4**), 2-*O*-sulphotransferase was employed to introduce the 2-*O*-sulpho group to the IdoA residue. The 2-*O*-sulphotransferase modification step was omitted during the synthesis of **5** to **8**. About 5–10 mg of each hexasaccharide was prepared for the current study. For the preparation of **5** to **8**, a HPLC purification step was necessary after the hexasaccharide backbone modified by C_5_-epimerase to separate the hexasaccharide containing an IdoA residue.

### MS analysis of oligosaccharides

MS analyses were performed at a Thermo LCQ-Deca. A syringe pump (Harvard Apparatus) was used to introduce the sample by direct infusion (35 μl min^−1^). Hexasaccharide (1 mg ml^−1^, 1 μl) was diluted by 200-fold with 10 mM ammonium bicarbonate. Experiments were carried out in negative ionization mode with the electrospray source set to 2 KV and 200 °C. The automatic gain control was set to 1 × 10^7^ for full scan MS. The MS data were acquired and processed using Xcalibur 1.3.

### Structural analysis of compound **1** to **8** by NMR

NMR experiments were performed at 298 K on Bruker Avance 700 MHz and 850 MHz spectrometer with Topsin 3.2 software. Samples (1.0 to 5.0 mg) were each dissolved in 0.5 ml D_2_O (99.996%, Sigma-Aldrich) and lyophilized three times to remove the exchangeable protons. The samples were re-dissolved in 0.4 ml D_2_O and transferred to NMR microtubes (O.D. 5 mm, Norrell). Chemical shifts are referenced to external 2,2-dimethyl-2-silapentane-5-sulphonate sodium salt (DSS, Sigma, Co.). Deuterated EDTA (Sigma, Co.) was added to remove the effect of paramagnetic ions. 1D ^1^H NMR experiments “zg” pulse sequence were performed with 64 scans and an acquisition time of 3.8 sec. 1D ^13^C NMR experiments “zgdc30” pulse sequence were performed with 30000 scans, 1.5 sec relaxation delay, and 1.0 sec acquisition time. 2D ^1^H-^1^H COSY experiments “cosygpmfphpp” pulse sequence were performed with 48 scans, 512 increments, 1.5 sec relaxation delay, and 120 msec acquisition time. 2D ^1^H-^1^H NOESY experiments “noesygpph” pulse sequence were performed with 48 scans, 512 increments, 1.5 sec relaxation delay, 120 msec acquisition time, and 300 msec mixing time. 2D ^1^H-^13^C HSQC experiments “hsqcgpph” pulse sequence were performed with 48 scans, 512 increments, 1.5 sec relaxation delay, and 120 msec acquisition time. 2D ^1^H-^13^C HMBC experiments “hmbcgpndqf” pulse sequence were performed with 72 scans, 512 increments, 1.5 sec relaxation delay, and 120 msec acquisition time. 2D ^1^H-^13^C HSQC-TOCSY experiments “hsqcetgpml” pulse sequence were performed with 48 scans, 512 increments, 80 msec TOCSY mixing time, 1.5 sec relaxation delay, and 120 msec acquisition time. 2D spectra were recorded with GARP carbon decoupling. 48 dummy scans were used prior to the start of acquisition. 2048 total points were collected in f2. ^13^C transmitter offset was set at 90.0 ppm. The polarization transfer delay CNST2 was set with a ^1^*J*_C-H_ coupling value of 145 Hz. The delay for evolution of long-range couplings CNST13 in HMBC was set with *J*_lr_ coupling value of 7.4 Hz.

The structural characterization were also performed by Varian Inova 500 MHz spectrometer equipped with 5 mm triple resonance XYZ or broadband PFG probe, and processed by VnmrJ 2.2D software. The constructs were analyzed by NMR experiments, including 1D- (“s2pul” pulse sequence ^1^H or ^13^C), 2D- (^1^H-^1^H “gCOSY” pulse sequence COSY, “TOCSY” pulse sequence TOCSY, ^1^H-^13^C “gHSQC” pulse sequence HSQC, “gHMBC” pulse sequence HMBC, and “gHSQCTOXY” pulse sequence HSQC-TOCSY) NMR. NMR experiments were performed at 298 K. Samples (5.0 to 10.0 mg) were each dissolved in 0.5 mL D_2_O (99.994%, Sigma, Co.) and lyophilized three times to remove the exchangeable protons.

### Molecular dynamics pre-processing

The hexasaccharides were created with the GLYCAM06 (version j-1) parameter set^39^ and sulphate parameters^29^, neutralized with Na^+^ ions, and solvated by the TIP5P water model in a truncated octahedral box 12 Å around the ligand^40^. Ensemble charges were developed for the GlcN residue according to the standard GLYCAM protocol^39^. Briefly, electrostatic potentials were calculated by GAUSSIAN09 at the HF/6-31G(*) level of theory for 100 snapshots at 0.5 ns intervals (50 ns total). Partial atomic charges were computed using the RESP module of AMBER14 by fitting to these electrostatic potentials with a restraint weight of 0.01^41^.

## Additional Information

**How to cite this article**: Hsieh, P.-H. *et al*. Uncovering the Relationship between Sulphation Patterns and Conformation of Iduronic Acid in Heparan Sulphate. *Sci. Rep*. **6**, 29602; doi: 10.1038/srep29602 (2016).

## Supplementary Material

Supplementary Information

Supplementary Video S1

## Figures and Tables

**Table 1:** Measurement of the population of conformers for IdoA2S and IdoA residues in hexasaccharides.

Hexasaccharides	Measured ^*3*^*J*_*H-H*_ couplings (Hz) (Calculated ^*3*^*J*_*H-H*_ couplings)				Population of conformers^d^			Sum of square difference^e^	NOE signals for measuring ^*2*^*S*_*O*_ conformer population
	^*3*^*J*_*H1-H2*_	^*3*^*J*_*H2-H3*_	^*3*^*J*_*H3-H4*_	^*3*^*J*_*H4-H5*_	^*1*^*C*_*4*_ a	^*2*^*S*_*O*_^b^	^*4*^*C*_*1*_ c		H2-H5/H4-H5^f^
**1**-IdoA2S	2.2 (2.7)	4.3 (4.1)	3.2 (3.0)	2.2 (2.1)	68%	32%	–	0.34	0.19
**2**-IdoA2S	2.2 (2.7)	4.3 (4.1)	3.2 (3.0)	2.3 (2.2)	68%	32%	–	0.34	0.21
**3**-IdoA2S	3.0 (3.6)	5.8 (5.5)	3.6 (3.6)	2.7 (2.5)	47%	53%	–	0.49	0.26
**4**-IdoA2S	3.7 (4.5)	7.4 (6.9)	4.0 (4.2)	3.1 (2.8)	25%	75%	–	1.02	0.34
**5**-IdoA	3.0 (3.3)	5.2 (5.1)	3.5 (3.4)	2.7 (2.4)	53%	47%	–	0.20	0.34
**6**-IdoA	3.1 (3.4)	5.3 (5.2)	3.5 (3.5)	2.7 (2.4)	51%	49%	–	0.19	0.35
**7**-IdoA	2.3 (2.8)	4.3 (4.1)	3.4 (3.2)	2.4 (2.2)	68%	29%	3%	0.37	0.22
**8**-IdoA	2.3 (2.7)	3.9 (3.8)	3.5 (3.2)	2.2 (2.2)	73%	19%	8%	0.26	0.19

^a^Calculated ^*3*^*J*_*H-H*_ values using Amber 14 with GLYCAM06 parameter^39^ for ^*1*^*C*_*4*_ conformer are 1.35 (^*3*^*J*_*H1-H2*_), 2.04 (^*3*^*J*_*H2-H3*_), 2.19 (^*3*^*J*_*H3-H4*_) and 1.69 Hz (^*3*^*J*_*H4-H5*_), respectively.

^b^Calculated ^*3*^*J*_*H-H*_ values using Amber 14 with GLYCAM06 parameter^39^ for ^*2*^*S*_*O*_ conformer are 5.55 (^*3*^*J*_*H1-H2*_), 8.48 (^*3*^*J*_*H2-H3*_), 4.82 (^*3*^*J*_*H3-H4*_) and 3.14 Hz (^*3*^*J*_*H4-H5*_), respectively.

^c^Calculated ^*3*^*J*_*H-H*_ values using Amber 14 with GLYCAM06 parameter^39^ for ^*4*^*C*_*1*_ conformer are 7.85 (^*3*^*J*_*H1-H2*_), 8.03 (^*3*^*J*_*H2-H3*_), 8.5 (^*3*^*J*_*H3-H4*_) and 5.08 Hz (^*3*^*J*_*H4-H5*_), respectively.

^d^Obtained by least sum of square difference analysis of the calculated and experimental values.

^e^The residual sum of squares (RSS) was used to determine how well the calculated population ratios fit the experimental data^42^.

^f^The calculated ratio of NOE H2-H5 signal and H4-H5 signal for pure ^*2*^*S*_*O*_, ^*1*^*C*_*4*_ and ^*4*^*C*_*1*_ conformer is 0.53, 0.05, and 0.03 respectively^39^.
