# Supplementary material for: Uncovering the Relationship between Sulphation Patterns and Conformation of Iduronic Acid in Heparan Sulphate
Source: Sci Rep. 2016 Jul 14;6:29602. doi: 10.1038/srep29602 (PMC4944151; doi:10.1038/srep29602)

Uncovering the Relationship between Sulphation Patterns and Conformation of Iduronic Acid in Heparan Sulphate

Supplementary information

Po-Hung Hsieh[1], David F. Thieker[2], Marco Guerrini[3], Robert J. Woods[2], and Jian Liu[1*]

1. Division of Chemical Biology and Medicinal Chemistry, Eshelman School of Pharmacy, University of North Carolina, Chapel Hill, North Carolina 27599, USA.
2. Complex Carbohydrate Research Center, University of Georgia, Athens, Georgia 30602, USA.
3. Istituto di Ricerche Chimiche e Biochimiche 'G. Ronzoni', via G. Colombo 81, 20133 Milan, Italy.

This work is supported in part by grants from National Institutes of Health (GM102137, HL094463 and P41 GM103390) and by a grant from Eshelman Institute for Innovation.

Corresponding author contact information: email, jian_liu@unc.edu; Tel, 919-843-6511

Experimental Procedures

HPLC Analysis-Both DEAE-HPLC and polyamine-based anion exchange (PAMN)-HPLC were used to analyze the purity of the products. The elution conditions for the HPLC analysis have been described elsewhere.[1] Briefly, for DEAE-HPLC method, the DEAE-NPR column ((0.46 × 7.5 cm, Tosohaas) was eluted with a linear gradient of NaCl in 20 mM sodium acetate buffer (pH 5.0) from 0 to 1 M in 60 min at a flow rate of 0.4 ml/min. For PAMN-HPLC, the column (silica-based polyamine column, 0.45 × 25 cm, YMC) was eluted with a linear gradient of KH_2PO_4 from 0 to 1 M in 40 min at a flow rate of 0.5 ml min^{-1}.

Energy minimization-Energy minimizations and MD simulations were performed using the *pmemd.cuda* module from AMBER14.[2] The system was minimized using the steepest descent method for the initial 1000 cycles before switching to conjugate gradient for the remaining 24,000 cycles. Three minimizations were performed with varying atomic restraints. Initially, cartesian restraints (10 kcal/mol $Å^2$) were applied to each atom of the hexasaccharide. The second stage involved restraining the ring atoms. All restraints were removed for the final minimization. During the remaining simulations, internal restraints were applied to the IdoA residue in order to maintain a $^1C_4$, ^2S_O, or $^4C_1$ conformation. Details of these restraints are provided in the Supplementary Table 4.

Molecular dynamics settings-MD simulations were performed with *pmemd.cuda* from AMBER14.[2] Electrostatic interactions were treated with the Particle-Mesh Ewald algorithm.[3] An 8 Å cutoff for non-bonded interactions was employed. The SHAKE algorithm was included in order to constrain hydrogen-containing bonds, enabling an

integration time step of 2 fs. The system was heated to 300 K under NVT conditions over 60 ps by employing the Berendsen thermostat with a coupling time constant of 1 ps, and allowed to equilibrate for a total of 1 ns under NPT conditions. A post-equilibration data set was collected for 1 µs, also under NPT conditions.

*Simulation data analysis***-** The Best-fit, Four-Membered Plane program was applied to the trajectory, and only those frames containing an idealized ring were considered for further analysis.[4] Structural metrics (i.e. torsion angles, hydrogen bond properties, and coordinate averaging) were generated using the cpptraj module of AmberTools14.[5] The Φ- and Ψ-glycosidic torsion angles were defined by the H1-C1-O4-C4 and C1-O4-C4-H4 atomic sequences, respectively. Theoretical J-coupling values were calculated by collecting the H1-H2, H2-H3, H3-H4, and H4-H5 torsion angles for the IdoA ring for the three ring conformations. An average torsion angle from 8 µs of data, 1 µs per compound, was transformed into a J-coupling value via the reparameterized Haasnoot Karplus equation-14.63*cos2(φ)-0.78*cos(φ)+0.60+∑λi{0.34-2.31cos2[sin(φ)+18.4|λi|]}.[6, 7] Images and RMSD values were collected using the Visual Molecular Dynamics program.[8]

Determination of the binding affinity to antithrombin-^{35}S-labeled **4** and **8** were prepared via 3-*O*-sulphation, using [^{35}S]PAPS (0.5 nmol, specific activity 2.2 × 10^4 cpm $pmol^{-1}$) and hexasaccharide **3** and **7** (5 µg), respectively, and 3-*O*-sulphotransferase (5 µg) in total 500 µl at 37°C overnight. The products were purified by DEAE-HPLC column. Affinity co-electrophoresis[9] was used for measurement of dissociation constant (K_d) of oligosaccharides. ^{35}S-labeled constructs (1500-2500 cpm) were loaded for lanes with antithrombin concentration at 0, 8, 16, 32, 60, 120, 250, 500, and 1000 nM. The

agarose gel was run with 300 mA electrophoresis for two hours, and was dried at room temperature overnight. The dried gel was analyzed by PhosphoImager (Amersham Biosciences, Storm 860). The retardation coefficient was calculated at $R = (M_0\text{-}M)/M_0$, where M_0 is the mobility of the oligosaccharide flow through in the lane without antithrombin, and M is the mobility of the sample in an individual lane. The retardation coefficient was then plotted against the retardation coefficient divided by its respective antithrombin concentration. The constant $-1/K_d$ is the slope of the trend line.

Determination of anti-Xa activity-Assays were based on a previously published method.[10, 11] Briefly, human factor Xa (Xa) (Enzyme Research Laboratories, South Bend, IN) was diluted to 0.074 µM with PBS, and antithrombin was diluted to 0.55 µM in 1 mg ml^{-1} bovine serum albumin. The chromogenic substrates, S-2765 was from Diapharma (Westchester, OH) and made up at 1 mg ml^{-1} in water. Hexasaccharide **4** or **8** was dissolved in PBS at various concentrations (0.01 to 6.4 µg ml^{-1}). The reaction mixture, which consisted of 20 µl of human plasma (Sigma-Aldrich) and 10 µl of the solution containing the sample, was incubated at room temperature for 2 min. Factor Xa (100 µl) was then added. After incubating at room temperature for 4 min, 30 µl of S-2765 substrate was added. The absorbance of the reaction mixture was measured at 405 nm continuously for 5 min using spectrophotometer. The absorbance values were plotted against the reaction time to measure the reaction rate. The initial reaction rates were used to measure the activity of Xa.

Supplementary Figure legends

Supplementary Fig 1. HPLC chromatograms and ESI-MS spectra of compound **1** to **4**. *Panel A* shows the ESI-MS spectrum (left) and HPLC chromatogram of **1**. The structure of **1** is shown at the top of *Panel A*. The calculated m/z values for **1** are shown in the ESI-MS spectrum. *Panel B* shows the ESI-MS spectrum (left) and HPLC chromatogram of **2**. The structure of **2** is shown at the top of *Panel B*. The calculated m/z values for **2** are shown in the ESI-MS spectrum. *Panel C* shows the ESI-MS spectrum (left) and HPLC chromatogram of **3**. The structure of **3** is shown at the top of *Panel C*. The calculated m/z values for **3** are shown in the ESI-MS spectrum. *Panel D* shows the ESI-MS spectrum (left) and HPLC chromatogram of **4**. The structure of **4** is shown at the top of *Panel D*. The calculated m/z values for **4** are shown in the ESI-MS spectrum.

Supplementary Fig 2. HPLC chromatograms and ESI-MS spectra of compound **5** to **8**. *Panel A* shows the ESI-MS spectrum (left) and HPLC chromatogram of **5**. The structure of **5** is shown at the top of *Panel A*. The calculated m/z values for **5** are shown in the ESI-MS spectrum. *Panel B* shows the ESI-MS spectrum (left) and HPLC chromatogram of **6**. The structure of **6** is shown at the top of *Panel B*. The calculated m/z values for **6** are shown in the ESI-MS spectrum. *Panel C* shows the ESI-MS spectrum (left) and HPLC chromatogram of **7**. The structure of **7** is shown at the top of *Panel C*. The calculated m/z values for **7** are shown in the ESI-MS spectrum. *Panel D* shows the ESI-MS spectrum (left) and HPLC chromatogram of **8**. The structure of **8** is shown at the top of *Panel D*. The calculated m/z values for **8** are shown in the ESI-MS spectrum.

Supplementary Fig 3. ^{1}H-NMR spectrum of **1**. The structure of **1** is shown at the top left corner. The anomeric proton signals are labeled. The area (4.5 to 5.7 ppm) displaying the signals from anomeric protons are enlarged.

Supplementary Fig 4. ^{13}C-NMR spectrum of **1**. The signals from anomeric carbons are indicated.

Supplementary Fig 5. 2D ^{1}H-^{13}C HSQC NMR spectrum of **1**. The proton and carbon cross-peak signals are assigned.

Supplementary Fig 6. 2D ^{1}H-^{1}H COSY NMR spectrum of **1**. The cross-peaks between the protons correlated to the anomeric protons signals are assigned.

Supplementary Fig 7. 2D ^{1}H-^{13}C HSQC-TOCSY NMR spectrum of **1**. The proton-carbon cross-peaks within a single saccharide residue are assigned.

Supplementary Fig 8. 2D ^{1}H-^{13}C HMBC key correlations of **1**. The correlations of three-bond proton-carbon coupling for the glycosidic linkages are assigned.

Supplementary Fig 9. 2D ^{1}H-^{1}H NOESY key correlations of **1**. Protons having NOE effects with the anomeric protons are assigned.

Supplementary Fig 10. ^{1}H-NMR spectrum of **2**. The structure of **2** is shown at the top left corner. The anomeric proton signals are labeled. The area (4.5 to 5.7 ppm) displayed the signals from anomeric protons are enlarged.

Supplementary Fig 11. ^{13}C-NMR spectrum of **2**. The signals from anomeric carbons are indicated.

Supplementary Fig 12. 2D ^{1}H-^{13}C HSQC NMR spectrum of **2**. The proton and carbon cross-peak signals are assigned.

Supplementary Fig 13. 2D ^{1}H-^{1}H COSY NMR spectrum of **2**. The cross-peak between the protons correlated to the anomeric protons signals are assigned.

Supplementary Fig 14. 2D ^{1}H-^{13}C HSQC-TOCSY NMR spectrum of **2**. The proton-carbon cross-peaks within a single saccharide residue are assigned.

Supplementary Fig 15. 2D ^{1}H-^{13}C HMBC key correlations of **2**. The correlations of three-bond proton-carbon coupling for the glycosidic linkages are assigned.

Supplementary Fig 16. 2D ^{1}H-^{1}H NOESY key correlations of **2**. Protons having NOE effects with the anomeric protons are assigned.

Supplementary Fig 17. ^{1}H-NMR spectrum of **3**. The structure of **3** is shown at the top left corner. The anomeric proton signals are labeled. The area (4.5 to 5.7 ppm) displaying the signals from anomeric protons are enlarged.

Supplementary Fig 18. ^{13}C-NMR spectrum of **3**. The signals from anomeric carbons are indicated.

Supplementary Fig 19. 2D ^{1}H-^{13}C HSQC NMR spectrum of **3**. The proton and carbon cross-peak signals are assigned.

Supplementary Fig 20. 2D ^{1}H-^{1}H COSY NMR spectrum of **3**. The cross-peak between the protons correlated to the anomeric protons signals are assigned.

Supplementary Fig 21. 2D ^{1}H-^{13}C HSQC-TOCSY NMR spectrum of **3**. The proton-carbon cross-peaks within a single saccharide residue are assigned.

Supplementary Fig 22. 2D ^{1}H-^{13}C HMBC key correlations of **3**. The correlations of three-bond proton-carbon coupling for the glycosidic linkages are assigned.

Supplementary Fig 23. 2D ^{1}H-^{1}H NOESY key correlations of **3**. Protons having NOE effects with the anomeric protons are assigned.

Supplementary Fig 24. ^{1}H-NMR spectrum of **4**. The structure of **4** is shown at the top left corner. The anomeric proton signals are labeled. The area (4.5 to 5.7 ppm) displaying the signals from anomeric protons are enlarged.

Supplementary Fig 25. ^{13}C-NMR spectrum of **4**. The signals from anomeric carbons are indicated.

Supplementary Fig 26. 2D ^{1}H-^{13}C HSQC NMR spectrum of **4**. The proton and carbon cross-peak signals are assigned.

Supplementary Fig 27. 2D ^{1}H-^{1}H COSY NMR spectrum of **4**. The cross-peaks between the protons correlated to the anomeric protons signals are assigned.

Supplementary Fig 28. 2D ^{1}H-^{13}C HSQC-TOCSY NMR spectrum of **4**. The proton-carbon cross-peaks within a single saccharide residue are assigned.

Supplementary Fig 29. 2D ^{1}H-^{13}C HMBC key correlations of **4**. The correlations of three-bond proton-carbon coupling for the glycosidic linkages are assigned.

Supplementary Fig 30. 2D ^{1}H-^{1}H NOESY key correlations of **4**. Protons having NOE effects with the anomeric protons are assigned.

Supplementary Fig 31. ^{1}H-NMR spectrum of **5**. The structure of **5** is shown at the top left corner. The anomeric proton signals are labeled. The area (4.5 to 5.7 ppm) displaying the signals from anomeric protons are enlarged.

Supplementary Fig 32. ^{13}C-NMR spectrum of **5**. The signals from anomeric carbons are indicated.

Supplementary Fig 33. 2D ^{1}H-^{13}C HSQC NMR spectrum of **5**. The proton and carbon cross-peak signals are assigned.

Supplementary Fig 34. 2D ^{1}H-^{1}H COSY NMR spectrum of **5**. The cross-peaks between the protons correlated to the anomeric protons signals are assigned.

Supplementary Fig 35. 2D ^{1}H-^{13}C HSQC-TOCSY NMR spectrum of **5**. The proton-carbon cross-peaks within a single saccharide residue are assigned.

Supplementary Fig 36. 2D ^{1}H-^{13}C HMBC key correlations of **5**. The correlations of three-bond proton-carbon coupling for the glycosidic linkages are assigned.

Supplementary Fig 37. 2D ^{1}H-^{1}H NOESY key correlations of **5**. Protons having NOE effects with the anomeric protons are assigned.

Supplementary Fig 38. ^{1}H-NMR spectrum of **6**. The structure of **6** is shown at the top left corner. The anomeric proton signals are labeled. The area (4.5 to 5.7 ppm) displaying the signals from anomeric protons are enlarged.

Supplementary Fig 39. ^{13}C-NMR spectrum of **6**. The signals from anomeric carbons are indicated.

Supplementary Fig 40. 2D ^{1}H-^{13}C HSQC NMR spectrum of **6**. The proton and carbon cross-peak signals are assigned.

Supplementary Fig 41. 2D ^{1}H-^{1}H COSY NMR spectrum of **6**. The cross-peaks between the protons correlated to the anomeric protons signals are assigned.

Supplementary Fig 42. 2D ^{1}H-^{13}C HSQC-TOCSY NMR spectrum of **6**. The proton-carbon cross-peaks within a single saccharide residue are assigned.

Supplementary Fig 43. 2D ^{1}H-^{13}C HMBC key correlations of **6**. The correlations of three-bond proton-carbon coupling for the glycosidic linkages are assigned.

Supplementary Fig 44. 2D ^{1}H-^{1}H NOESY key correlations of **6**. Protons having NOE effects with the anomeric protons are assigned.

Supplementary Fig 45. ^{1}H-NMR spectrum of **7**. The structure of **7** is shown at the top left corner. The anomeric proton signals are labeled. The area (4.5 to 5.7 ppm) displaying the signals from anomeric protons are enlarged.

Supplementary Fig 46. ^{13}C-NMR spectrum of **7**. The signals from anomeric carbons are indicated.

Supplementary Fig 47. 2D ^{1}H-^{13}C HSQC NMR spectrum of **7**. The proton and carbon cross-peak signals are assigned.

Supplementary Fig 48. 2D ^{1}H-^{1}H COSY NMR spectrum of **7**. The cross-peaks between the protons correlated to the anomeric protons signals are assigned.

Supplementary Fig 49. 2D ^{1}H-^{13}C HSQC-TOCSY NMR spectrum of **7**. The proton-carbon cross-peaks within a single saccharide residue are assigned.

Supplementary Fig 50. 2D ^{1}H-^{13}C HMBC key correlations of **7**. The correlations of three-bond proton-carbon coupling for the glycosidic linkages are assigned.

Supplementary Fig 51. 2D ^{1}H-^{1}H NOESY key correlations of **7**. Protons having NOE effects with the anomeric protons are assigned.

Supplementary Fig 52. ^{1}H-NMR spectrum of **8**. The structure of **8** is shown at the top left corner. The anomeric proton signals are labeled. The area (4.5 to 5.7 ppm) displaying the signals from anomeric protons are enlarged.

Supplementary Fig 53. ^{13}C-NMR spectrum of **8**. The signals from anomeric carbons are indicated.

Supplementary Fig 54. 2D ^{1}H-^{13}C HSQC NMR spectrum of **8**. The proton and carbon cross-peak signals are assigned.

Supplementary Fig 55. 2D ^{1}H-^{1}H COSY NMR spectrum of **8**. The cross-peaks between the protons correlated to the anomeric protons signals are assigned.

Supplementary Fig 56. 2D ^{1}H-^{13}C HSQC-TOCSY NMR spectrum of **8**. The proton-carbon cross-peaks within a single saccharide residue are assigned.

Supplementary Fig 57. 2D ^{1}H-^{13}C HMBC key correlations of **8**. The correlations of three-bond proton-carbon coupling for the glycosidic linkages are assigned.

Supplementary Fig 58. 2D ^{1}H-^{1}H NOESY key correlations of **8**. Protons having NOE effects with the anomeric protons are assigned.

Reference

1. Liu, R. et al. Chemoenzymatic design of heparan sulphate oligosaccharides. *J Biol Chem* **285**, 34240-34249 (2010).
2. Case, D.A. et al. AMBER 14. University of California, San Francisco (2014).
3. Darden, T., York, D. & Pedersen, L.C. Particle Mesh Ewald-An N.log(N) method for Ewald sums in Large Systems. *J. Chem. Phys.* **98**, 10089-10091 (1993).
4. Makeneni, S., Foley, B.L. & Woods, R.J. BFMP: A method for discretizing and visualizing pyranose conformations. *J. Chem. Inf. Model.* **54**, 2744-2750 (2014).
5. Roe, D.R. & Cheatham III, T.E. PTRAJ and CPPTRAJ: Software for Processing and Analysis of Molecular Dynamics Trajectory Data. *J. Chem. Theory Comput* **9**, 3084-3095 (2013).
6. Haasnoot, C., de Leeuw, F.A. & Atltona, C. The relationship between proton-proton NMR coupling constants and substituent electronegativities-I: an empirical generation of the Karplus equation. *Tetrahedron* **36**, 2783-2792 (1980).
7. Altona, C. et al. Empirical group electronegativities for vicinal NMR proton-proton couplings along a C-C bond: solvent effects and reparameterization of the Haasnoot equation. *Magn. Reson. Chem.* **32**, 670-678 (1994).
8. Humphrey, W., Dalke, A. & Schulten, K. VMD: Visual molecular dynamics. *J. Mol. Graph.* **14**, 33-38 (1996).
9. Lee, M.K. & Lander, A.D. Analysis affinity and structural selectivity in the binding of proteins to glycosamineglycans: Development of a sensitive electrophoretc approach. *Proc. Natl. Acad. Sci. USA* **88**, 2768-2772 (1991).

10. Zhang, L. et al. 6-O-sulphotransferase-1 represents a critical enzyme in the anticoagulant heparan sulphate biosynthetic pathway. *J. Biol. Chem.* **276**, 42311-42321 (2001).
11. Duncan, M.B., Chen, J., Krise, J.P. & Liu, J. The biosynthesis of anticoagulant heparan sulphate by the heparan sulphate 3-O-sulphotransferase isoform 5. *Biochim Biophys Acta* **1671**, 34-43 (2004).

Supplementary Video 1. The IdoA2S ring transitions from the $^{1}C_{4}$ to the $^{2}S_{O}$ and then to the $^{4}C_{1}$ conformation; colored orange, green, and purple, respectively. The video was created by morphing between three structures that were obtained from the MD simulations.

Supplementary Table 1. $^{1}H/^{13}C$ NMR chemical shift assignments (in p.p.m.) of compound **1 – 4** (25°C, D_2O, pD 7.4, 3 mM EDTA)

	1	2	3	4	5	6a	6b
**1** ($GlcNH_2$-GlcA-GlcNS-IdoA2S-GlcNS-GlcA-pnp)							
I	5.27	3.69	3.95	3.87	4.00		
	102.0	75.1	78.5	79.7	79.6		
II	5.58	3.26	3.66	3.70	3.80	3.83	3.84
	100.3	61.0	72.4	79.7	73.8	62.4	62.4
III	5.24	4.31	4.22	4.02	4.83		
	101.8	77.3	70.4	78.3	70.9		
IV	5.29	3.24	3.72	3.67	3.90	3.84	3.85
	99.8	60.4	72.1	80.6	73.2	62.3	62.3
V	4.50	3.40	3.75	3.83	3.83		
	104.9	75.8	78.6	78.7	78.8		
VI	5.60	3.10	3.74	3.44	3.72	3.78	3.79
	99.1	57.2	73.5	72.0	75.0	62.6	62.6
**2** (GlcNS-GlcA-GlcNS-IdoA2S-GlcNS-GlcA-pnp)							
I	5.28	3.70	3.96	3.88	4.02		
	102.0	75.1	78.5	79.7	79.4		
II	5.59	3.26	3.66	3.70	3.80	3.84	3.84
	100.3	61.0	72.4	79.8	73.8	62.3	62.3
III	5.24	4.31	4.22	4.02	4.83		
	101.8	77.2	70.3	78.4	70.7		
IV	5.30	3.25	3.71	3.69	3.85	3.87	3.87
	100.0	60.4	72.1	80.6	73.4	62.1	62.1
V	4.53	3.39	3.84	3.78	3.82		
	104.9	75.5	78.6	78.9	79.1		
VI	5.62	3.22	3.59	3.45	3.68	3.77	3.77
	100.0	60.8	73.9	72.4	74.4	62.9	62.9
**3** (GlcNS6S-GlcA-GlcNS6S-IdoA2S-GlcNS6S-GlcA-pnp)							
I	5.29	3.71	3.95	3.90	4.04		
	102.0	75.1	78.5	79.7	79.3		
II	5.60	3.30	3.67	3.77	3.95	4.22	4.38
	100.3	60.7	72.4	78.5	71.9	69.0	69.0
III	5.23	4.32	4.19	4.09	4.80		
	101.8	78.3	71.5	78.8	71.7		
IV	5.40	3.28	3.69	3.74	4.05	4.22	4.44
	99.6	60.2	72.1	79.7	71.6	68.6	68.6
V	4.60	3.38	3.84	3.78	3.82		
	104.5	75.5	78.6	79.4	79.0		
VI	5.61	3.25	3.61	3.56	3.87	4.15	4.33
	100.3	60.6	73.8	71.7	72.5	69.0	69.0
**4** (GlcNS6S-GlcA-GlcNS3S6S-IdoA2S-GlcNS6S-GlcA-pnp)							
I	5.28	3.71	3.95	3.90	4.00		
	102.0	75.1	78.5	80.0	79.5		
II	5.58	3.30	3.65	3.79	3.95	4.22	4.44
	100.4	60.8	72.4	78.2	72.0	68.9	68.9
III	5.18	4.31	4.15	4.13	4.78		
	102.2	79.6	73.0	78.6	72.8		
IV	5.50	3.43	4.35	3.95	4.15	4.25	4.47
	98.8	59.4	78.9	75.6	72.3	68.7	68.7
V	4.61	3.40	3.83	3.82	3.76		
	103.8	75.5	79.5	78.8	79.8		
VI	5.61	3.24	3.60	3.56	3.87	4.14	4.35
	100.2	60.7	73.8	71.7	72.4	69.0	69.0

Supplementary Table 2. $^{1}H/^{13}C$ NMR chemical shift assignments (in p.p.m.) of compound **5 – 8** (25°C, D_2O, pD 7.4, 3 mM EDTA)

	1	2	3	4	5	6a	6b
**5** (GlcNH$_2$-GlcA-GlcNS-IdoA-GlcNS-GlcA-pnp)							
I	5.28	3.69	3.96	3.87	4.02		
	102.0	75.1	78.6	79.3	79.3		
II	5.61	3.27	3.65	3.70	3.79	3.79	3.79
	100.2	61.0	72.4	79.9	73.7	62.4	62.4
III	4.95	3.73	4.10	4.04	4.84		
	104.3	71.7	70.9	77.5	71.5		
IV	5.35	3.22	3.69	3.68	3.82	3.83	3.87
	98.2	60.2	72.3	80.6	73.3	62.2	62.2
V	4.52	3.40	3.76	3.81	3.87		
	104.7	75.9	78.6	78.6	78.6		
VI	5.69	3.31	3.84	3.48	3.72	3.79	3.79
	97.9	56.9	72.1	71.8	75.1	62.5	62.5
**6** (GlcNS-GlcA-GlcNS-IdoA-GlcNS-GlcA-pnp)							
I	5.28	3.69	3.96	3.87	4.00		
	102.0	75.1	78.6	79.4	79.5		
II	5.61	3.27	3.64	3.70	3.81	3.80	3.80
	100.2	60.9	72.5	79.8	73.7	62.4	62.4
III	4.94	3.72	4.09	4.04	4.76		
	104.4	71.9	71.3	77.6	71.9		
IV	5.36	3.23	3.67	3.68	3.86	3.83	3.87
	98.0	60.2	72.4	80.6	73.3	62.2	62.2
V	4.52	3.39	3.83	3.78	3.80		
	105.0	75.5	78.6	78.9	79.2		
VI	5.63	3.22	3.59	3.46	3.69	3.77	3.77
	100.0	60.8	73.9	72.4	74.3	62.9	62.9
**7** (GlcNS6S-GlcA-GlcNS6S-IdoA-GlcNS6S-GlcA-pnp)							
I	5.29	3.71	3.97	3.89	4.01		
	102.0	75.1	78.6	79.4	79.4		
II	5.62	3.30	3.66	3.73	3.99	4.20	4.32
	100.2	60.8	72.6	80.2	71.7	68.9	68.9
III	5.01	3.78	4.11	4.05	4.81		
	104.7	71.2	70.2	77.1	71.1		
IV	5.34	3.25	3.68	3.75	4.03	4.19	4.46
	98.1	60.0	72.3	79.7	71.5	68.6	68.6
V	4.60	3.38	3.85	3.78	3.81		
	104.5	75.6	78.6	79.3	79.1		
VI	5.62	3.25	3.61	3.56	3.88	4.15	4.34
	100.2	60.6	73.8	71.7	72.5	69.1	69.1
**8** (GlcNS6S-GlcA-GlcNS3S6S-IdoA-GlcNS6S-GlcA-pnp)							
I	5.29	3.71	3.97	3.89	4.01		
	102.0	75.1	78.6	79.5	79.4		
II	5.62	3.29	3.67	3.73	3.99	4.19	4.32
	100.2	60.9	72.6	80.4	71.7	68.9	68.9
III	5.04	3.75	4.16	4.10	4.83		
	104.7	71.3	69.5	77.3	70.9		
IV	5.37	3.43	4.38	3.97	4.06	4.21	4.50
	98.3	59.3	78.7	75.5	72.3	68.6	68.6
V	4.61	3.40	3.83	3.83	3.75		
	104.0	75.5	78.8	79.5	79.8		
VI	5.61	3.24	3.60	3.56	3.88	4.15	4.35
	100.2	60.7	73.8	71.7	72.4	69.0	69.0

Supplementary Table 3. All hydrogen bonding occupancies greater than 15% are reported below. Residues are specified by a letter (see figure below), and atom names match those from the GLYCAM force field. Occupancies for oxygen atoms within carboxyl groups were combined and are listed as O6. Oxygen atoms within sulphate groups were similarly combined and are described by the site of attachment (i.e. 2-linked sulphate is 2-SO_3).

Hexasaccharides (**1** to **8**)

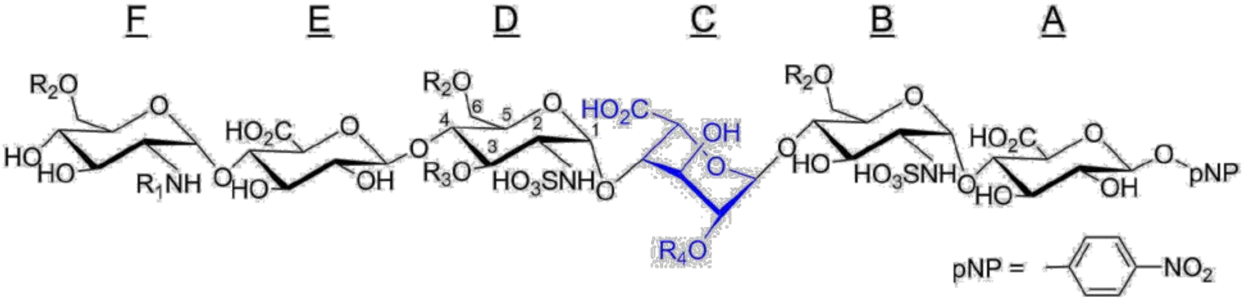

1. $R_1 = R_2 = R_3 = -H; R_4 = -SO_3H$
2. $R_1 = -SO_3H; R_2 = R_3 = -H; R4 = -SO_3H$
3. $R_1 = R_2 = -SO_3H; R_3 = -H; R_4 = -SO_3H$
4. $R_1 = R_2 = R_3 = R_4 = -SO_3H$
5. $R_1 = R_2 = R_3 = R_4 = -H$
6. $R_1 = -SO_3H; R_2 = R_3 = R_4 = -H$
7. $R_1 = R_2 = -SO_3H; R_3 = R_4 = -H$
8. $R_1 = R_2 = R_3 = -SO_3H; R_4 = -H$

Acceptor Residue	Acceptor Atom	Donor Residue	Donor Atom	Compound	Conformation	Occupancy
A	O6	B	H6O	1	1C4	21%
				1	2So	21%
				1	4C1	31%
				2	2So	20%
				2	1C4	24%
				2	4C1	26%
				5	2So	16%
				5	4C1	27%
				6	1C4	19%
				6	2So	22%
B	N-SO3	A	H3O	1	1C4	45%
				1	2So	50%
				1	4C1	52%
				2	1C4	48%
				2	2So	50%
				2	4C1	52%
				3	1C4	43%
				3	2So	50%
				3	4C1	53%
				4	1C4	45%
				4	2So	49%
				4	4C1	51%
				5	1C4	50%
				5	2So	47%
				5	4C1	46%
				6	1C4	47%

				6	2So	50%
				6	4C1	45%
				7	1C4	49%
				7	2So	47%
				7	4C1	48%
				8	1C4	49%
				8	2So	46%
				8	4C1	48%
B	N-SO3	B	H3O	1	1C4	21%
				1	2So	16%
				1	4C1	19%
				2	1C4	21%
				2	2So	15%
				2	4C1	18%
				3	1C4	22%
				3	2So	16%
				3	4C1	18%
				4	1C4	22%
				4	2So	17%
				4	4C1	20%
				5	1C4	19%
				5	2So	18%
				5	4C1	23%
				6	1C4	21%
				6	2So	18%
				6	4C1	23%
				7	1C4	19%
				7	2So	19%
				7	4C1	20%
				8	1C4	19%
				8	2So	18%
				8	4C1	20%
C	O2	D	H2N	3	1C4	16%
C	O5	B	H3O	1	4C1	21%
				2	4C1	19%
C	O6	D	H6O	1	1C4	26%
				1	2So	21%
				2	1C4	23%
				2	2So	17%
				5	1C4	25%
				5	2So	31%
				6	1C4	29%
				6	2So	23%
C	2-SO3	C	H3O	1	2So	38%
				1	4C1	35%
				2	2So	39%

				2	4C1	35%
				3	2So	31%
				3	4C1	31%
				4	2So	32%
D	3-SO3	E	H2O	4	1C4	19%
D	3-SO3	D	H2N	4	2So	30%
				4	4C1	46%
				8	1C4	32%
				8	4C1	47%
D	N-SO3	D	H3O	1	1C4	30%
				1	2So	23%
				1	4C1	28%
				2	1C4	30%
				2	2So	19%
				2	4C1	28%
				3	1C4	31%
				3	2So	25%
				3	4C1	25%
				5	1C4	40%
				5	2So	22%
				5	4C1	26%
				6	1C4	40%
				6	2So	18%
				6	4C1	25%
				7	1C4	41%
				7	2So	22%
				7	4C1	27%
D	N-SO3	C	H2O	5	1C4	17%
				6	1C4	17%
				7	1C4	15%
				8	1C4	47%
D	N-SO3	C	H3O	1	4C1	15%
				2	4C1	16%
				3	4C1	17%
				4	2So	20%
				4	4C1	48%
				5	2So	26%
				5	4C1	20%
				6	2So	25%
				6	4C1	21%
				7	2So	21%
				7	4C1	20%
				8	4C1	54%
E	O6	F	H6O	1	1C4	26%
				1	2So	27%

				1	4C1	22%
				2	1C4	27%
				2	2So	22%
				2	4C1	29%
				5	1C4	29%
				5	2So	25%
				5	4C1	26%
				6	1C4	24%
				6	2So	22%
				6	4C1	30%
F	N-SO3	E	H3O	2	1C4	34%
				2	2So	30%
				2	4C1	35%
				3	1C4	32%
				3	2So	31%
				3	4C1	30%
				4	1C4	32%
				4	2So	32%
				4	4C1	30%
				6	1C4	35%
				6	2So	28%
				6	4C1	34%
				7	1C4	32%
				7	2So	31%
				7	4C1	33%
				8	1C4	30%
				8	2So	30%
				8	4C1	31%
F	N-SO3	F	H3O	2	1C4	34%
				2	2So	35%
				2	4C1	33%
				3	1C4	36%
				3	2So	36%
				3	4C1	36%
				4	1C4	37%
				4	2So	36%
				4	4C1	38%
				6	1C4	34%
				6	2So	38%
				6	4C1	34%
				7	1C4	36%
				7	2So	36%
				7	4C1	36%
				8	1C4	37%
				8	2So	36%
				8	4C1	37%

Supplementary Table 4. Internal restraints applied for IdoA during MD simulation. The IdoA ring conformations were restrained by limiting the rotational freedom of each dihedral of the 6-membered ring. The restraint is imposed by an energy potential in the shape of a flat-welled parabola which becomes linear past specified torsion angles (R1 and R4). The R2 and R3 parameters define the points where the parabola flattens. The force constants (RK2 and RK3) were set at 300 kcal/mol for each ring conformation.

All<br>Rk2=300<br>Rk3=300				
$^{1}C_{4}$	R1	R2	R3	R4
O5-C1-C2-C3	-74.6	-64.6	-44.6	-34.6
C1-C2-C3-C4	33.6	43.6	63.6	73.6
C2-C3-C4-C5	-72.9	-62.9	-42.9	-32.9
C3-C4-C5-O5	34.1	44.1	64.1	74.1
C4-C5-O5-C1	-77.9	-67.9	-47.9	-37.9
C5-O5-C1-C2	36.4	46.4	66.4	76.4
$^{2}S_{o}$	R1	R2	R3	R4
O5-C1-C2-C3	9.7	19.7	39.7	49.7
C1-C2-C3-C4	-77.1	-67.1	-47.1	-37.1
C2-C3-C4-C5	-1.6	8.4	28.4	38.4
C3-C4-C5-O5	24.1	34.1	54.1	64.1
C4-C5-O5-C1	-98.4	-88.4	-68.4	-58.4
C5-O5-C1-C2	14.5	24.5	44.5	54.5
$^{4}C_{1}$	R1	R2	R3	R4
O5-C1-C2-C3	27.2	37.2	57.2	67.2
C1-C2-C3-C4	-67.7	-57.7	-37.7	-27.7
C2-C3-C4-C5	35.1	45.1	65.1	75.1
C3-C4-C5-O5	-79.3	-69.3	-49.3	-39.3
C4-C5-O5-C1	45.1	55.1	75.1	85.1
C5-O5-C1-C2	-77.2	-67.2	-47.2	-37.2

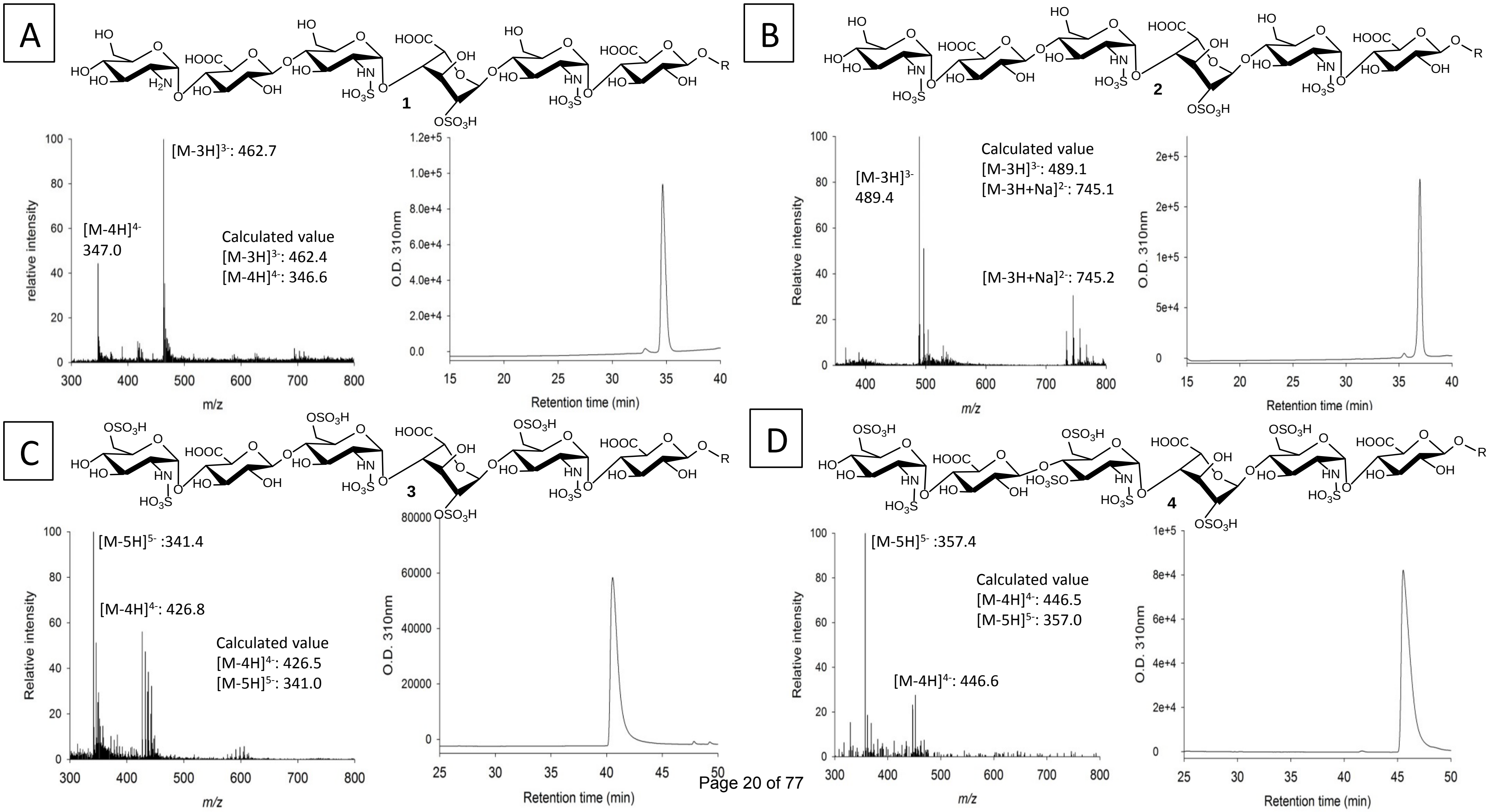

Supplementary Fig 1. Mass and HPLC analysis of **1**, **2**, **3** and **4**

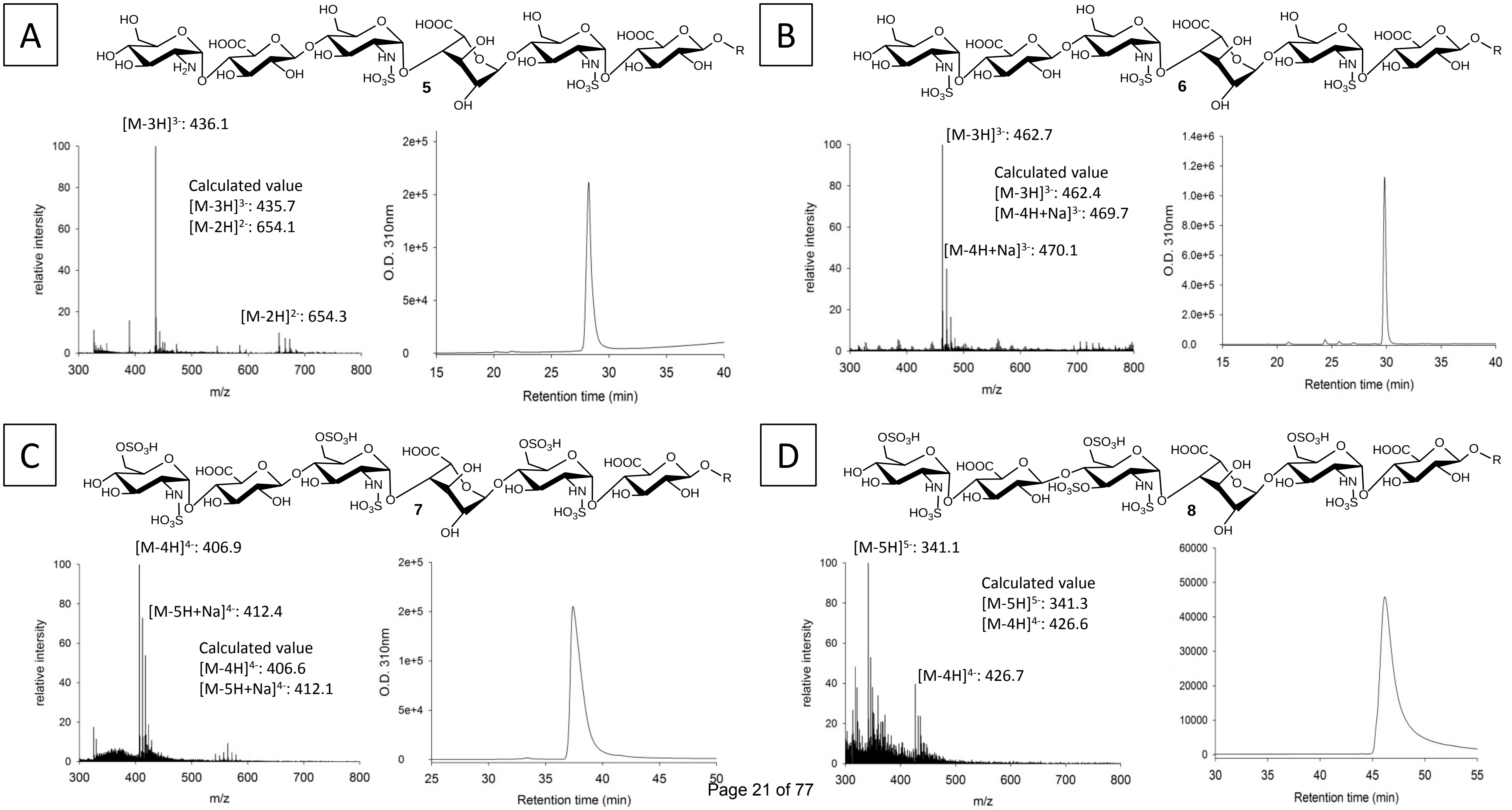

Supplementary Fig 2. Mass and HPLC analysis of **5**, **6**, **7** and **8**

Supplementary Fig 3. ^{1}H-NMR spectrum of **1**

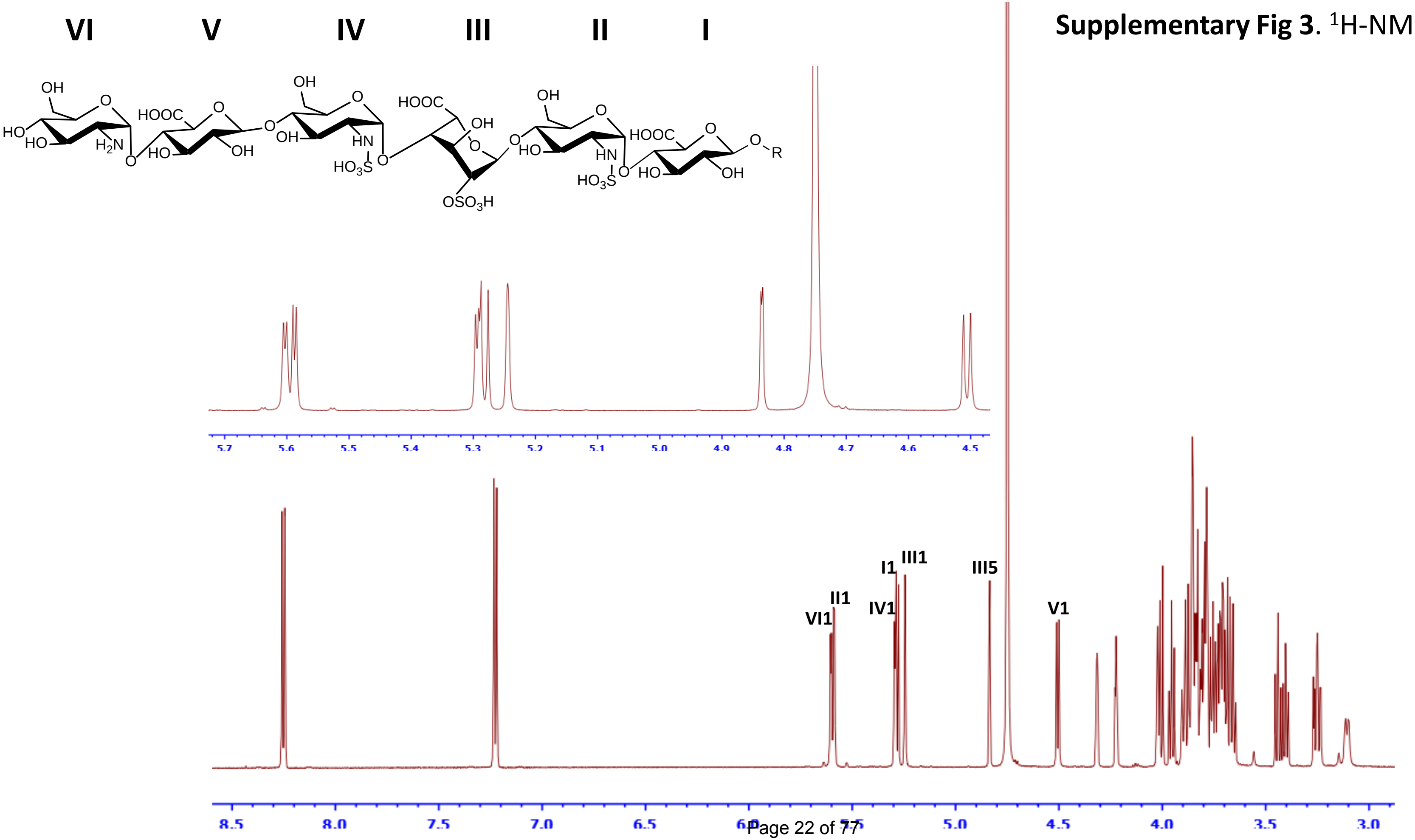

Supplementary Fig 4. ^{13}C-NMR spectrum of **1**

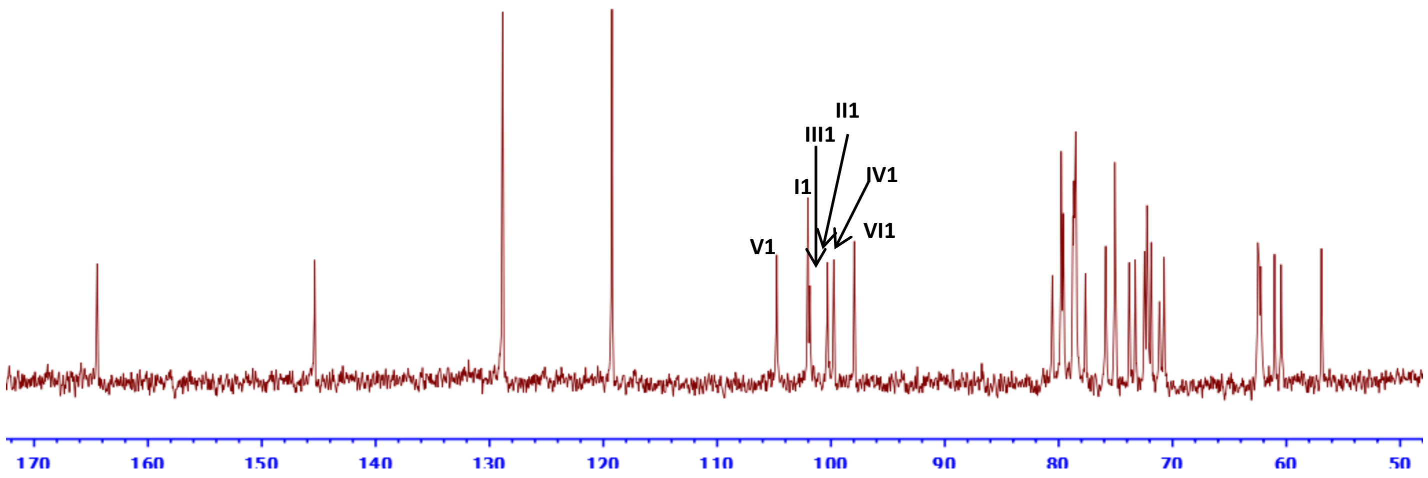

Supplementary Fig 5. 2D ^{1}H-^{13}C HSQC NMR spectrum of **1**

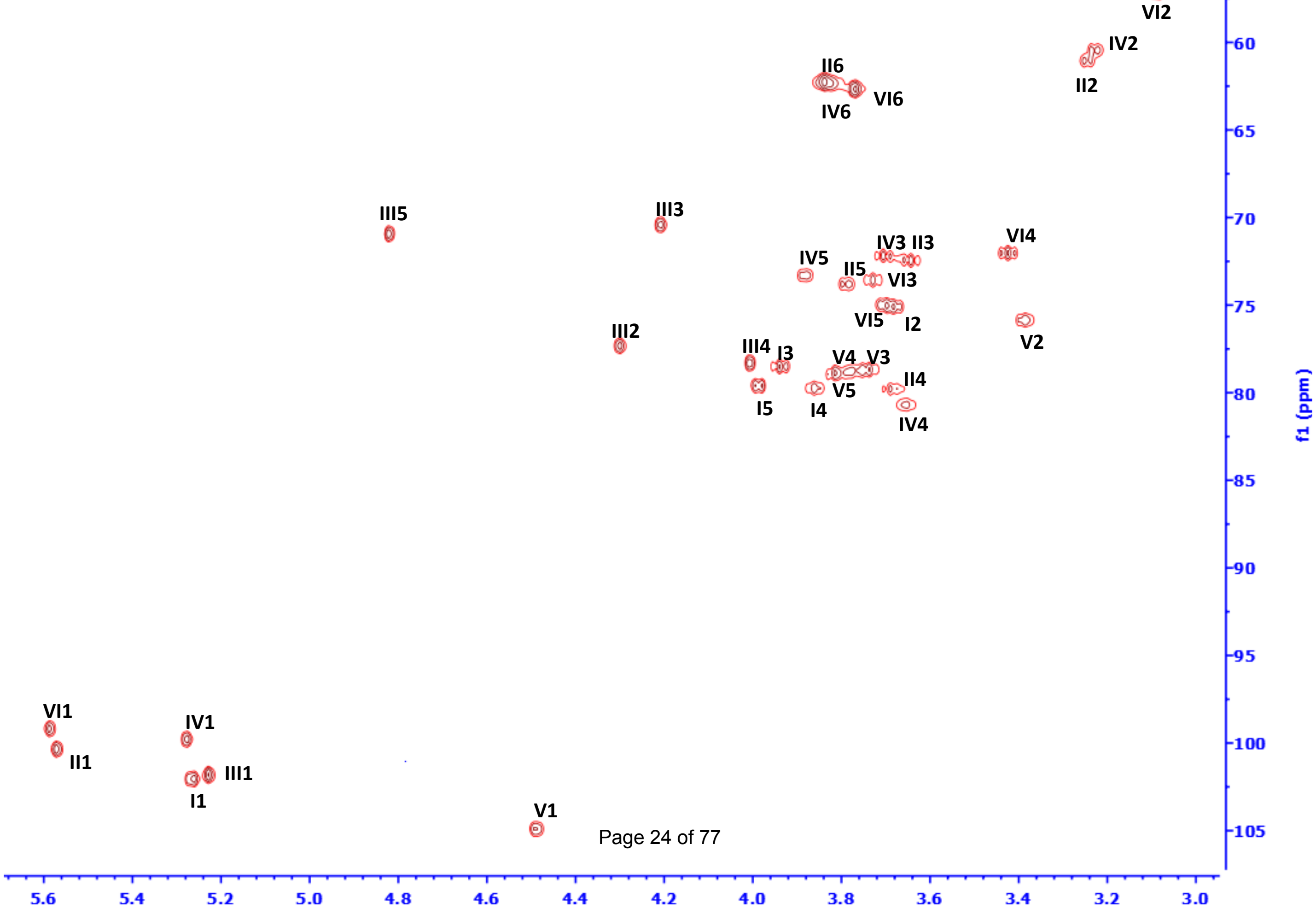

Supplementary Fig 6. 2D ^1H-^1H COSY NMR spectrum of **1**

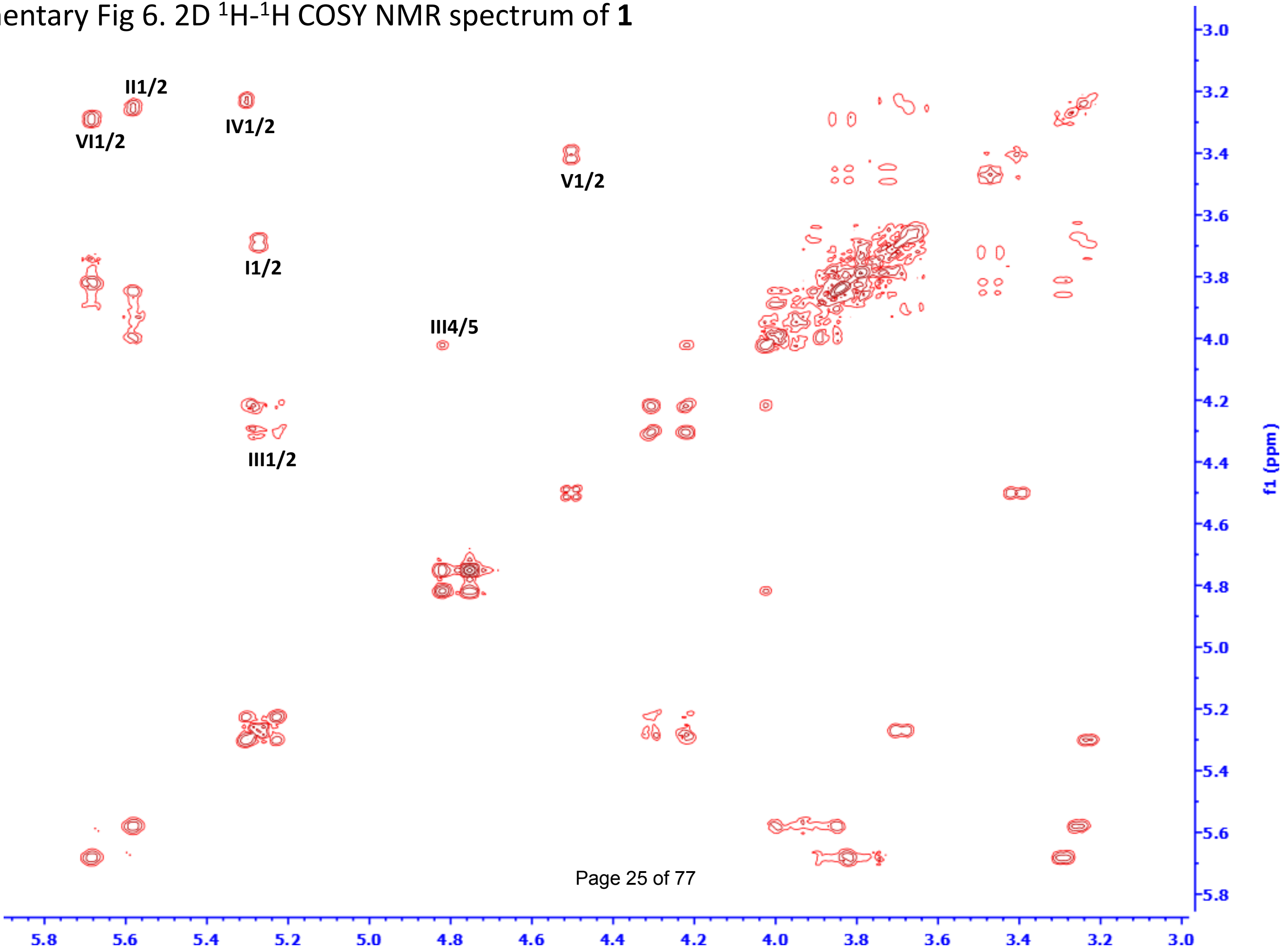

Supplementary Fig 7. 2D ^{1}H-^{13}C HSQC-TOCSY NMR spectrum of **1**

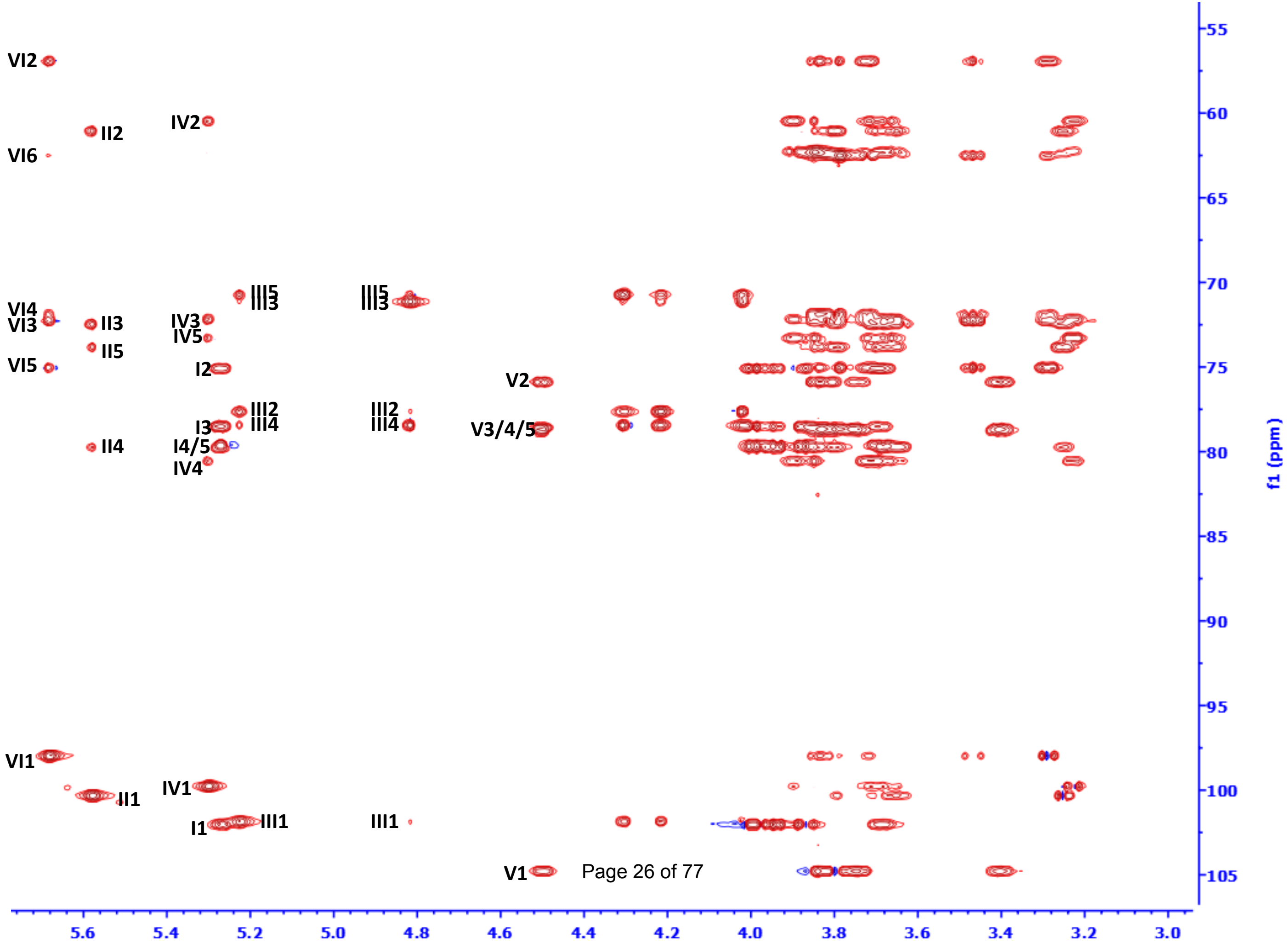

Supplementary Fig 8. 2D ^{1}H-^{13}C HMBC key correlations of **1**

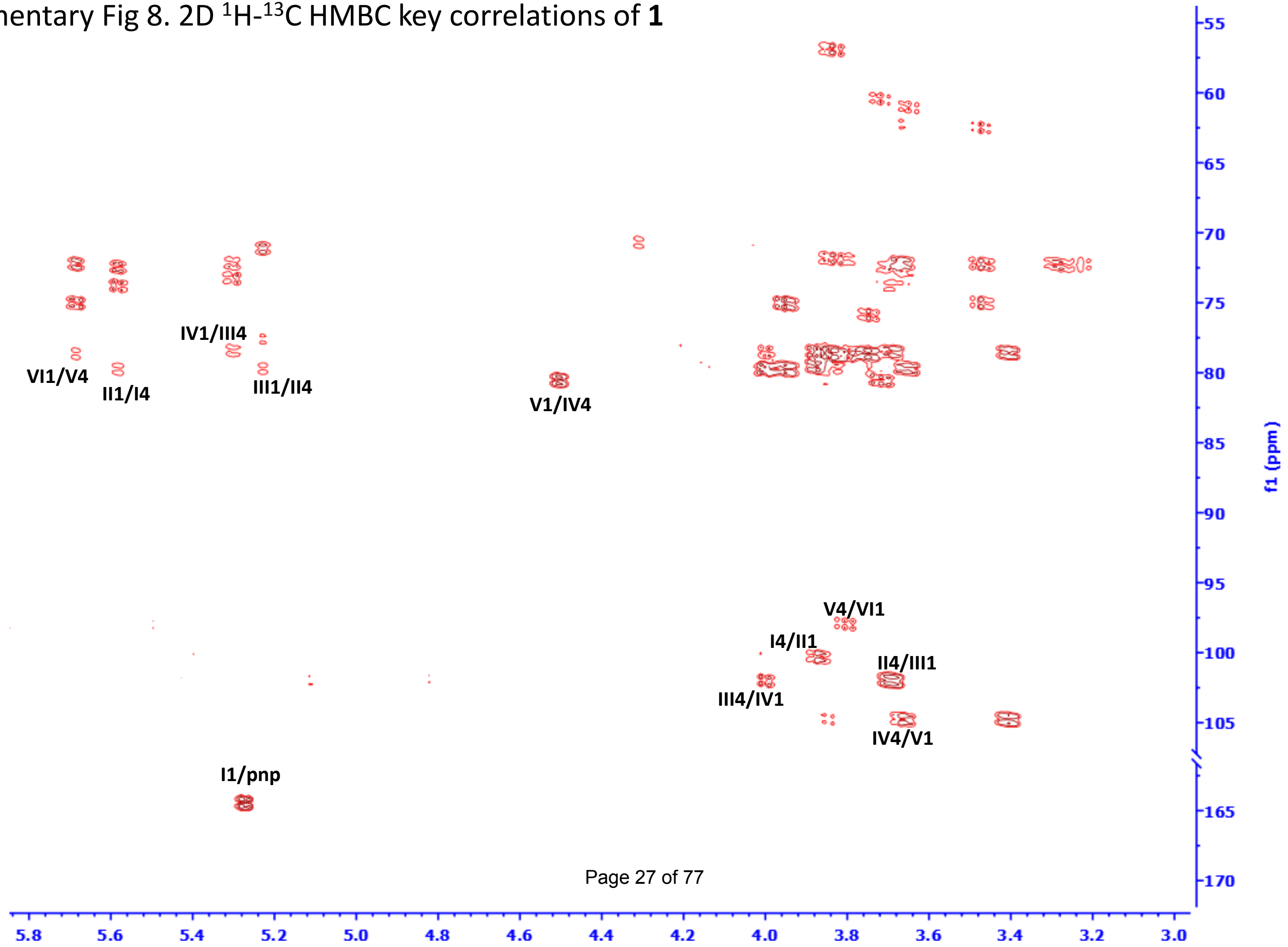

Supplementary Fig 9. 2D ^1H-^1H NOESY key correlations of **1**

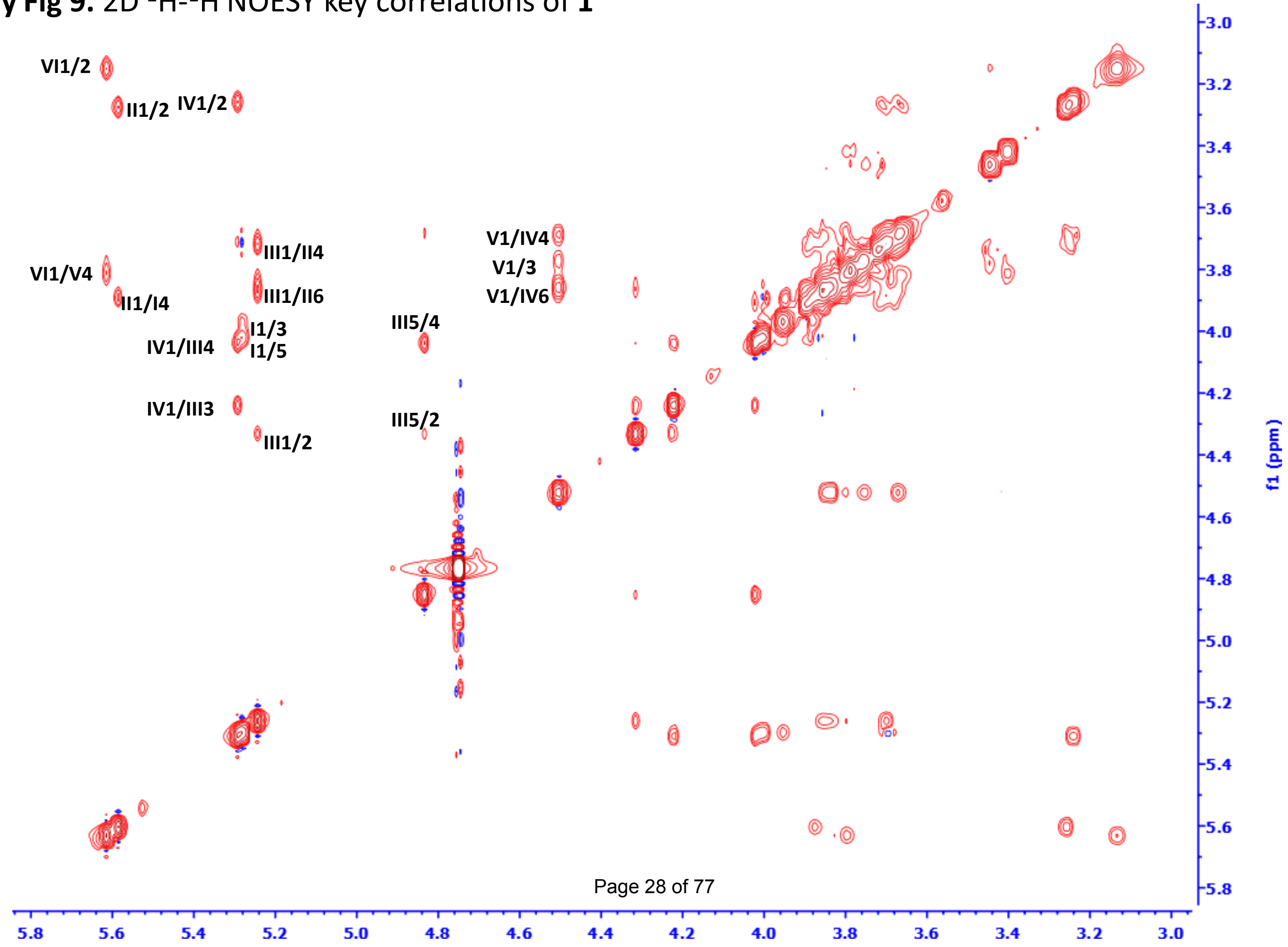

Supplementary Fig 10. ^{1}H-NMR spectrum of **2**

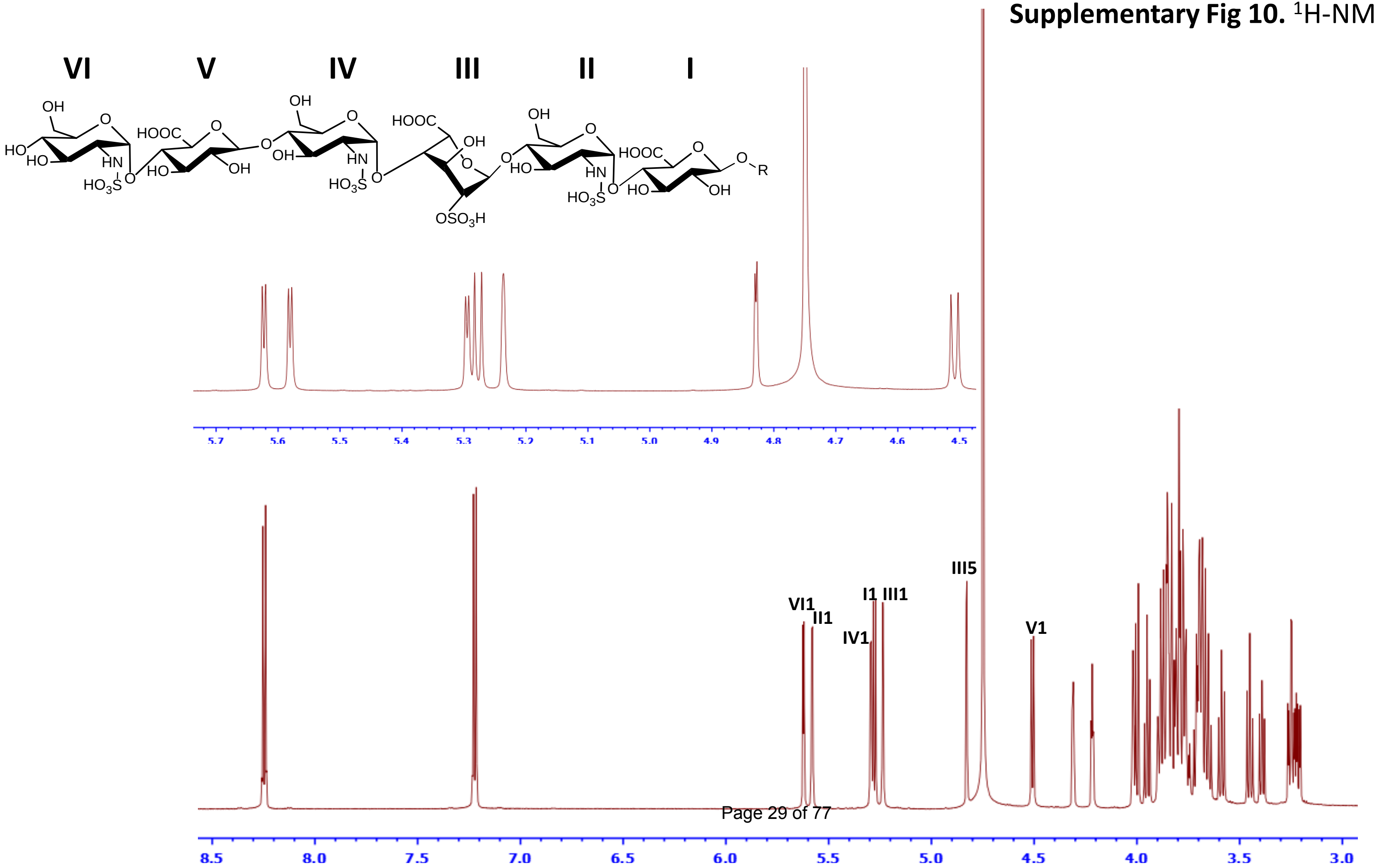

Supplementary Fig 11. ^{13}C-NMR spectrum of **2**

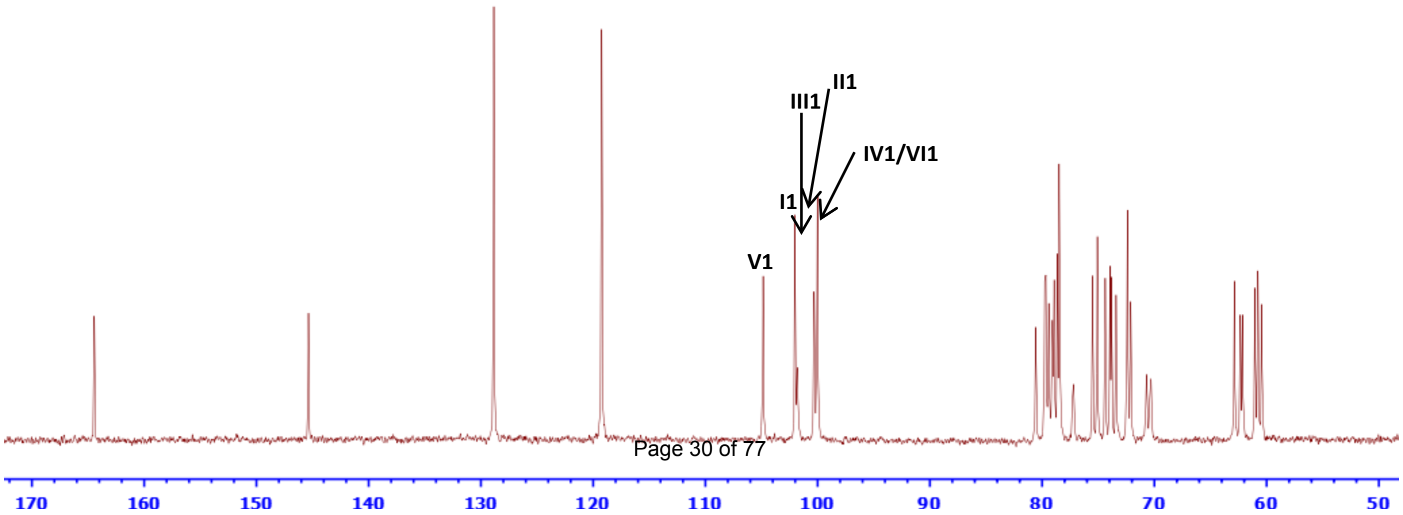

Supplementary Fig 12. 2D ^{1}H-^{13}C HSQC NMR spectrum of **2**

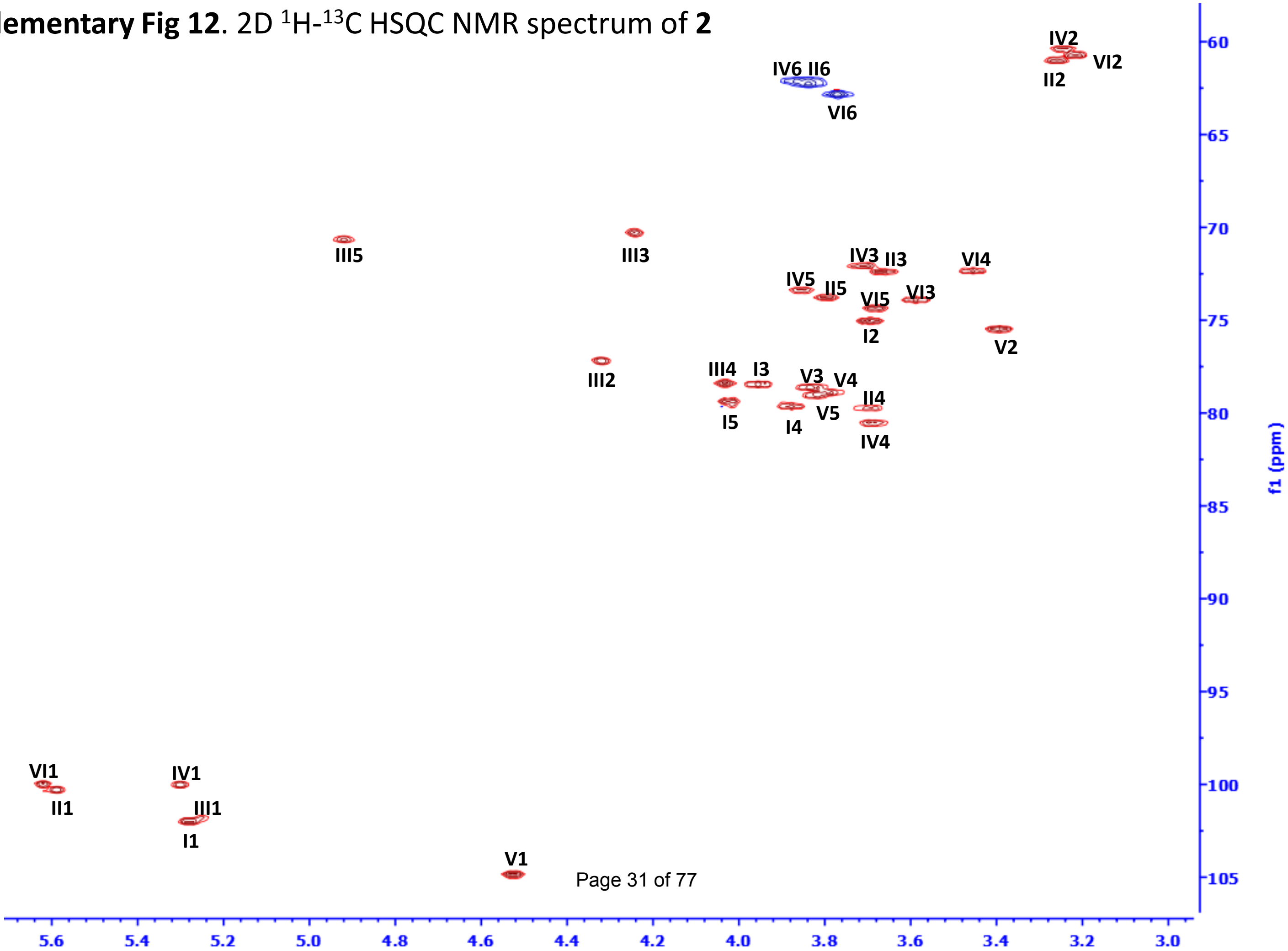

Supplementary Fig 13. 2D ^{1}H-^{1}H COSY NMR spectrum of **2**

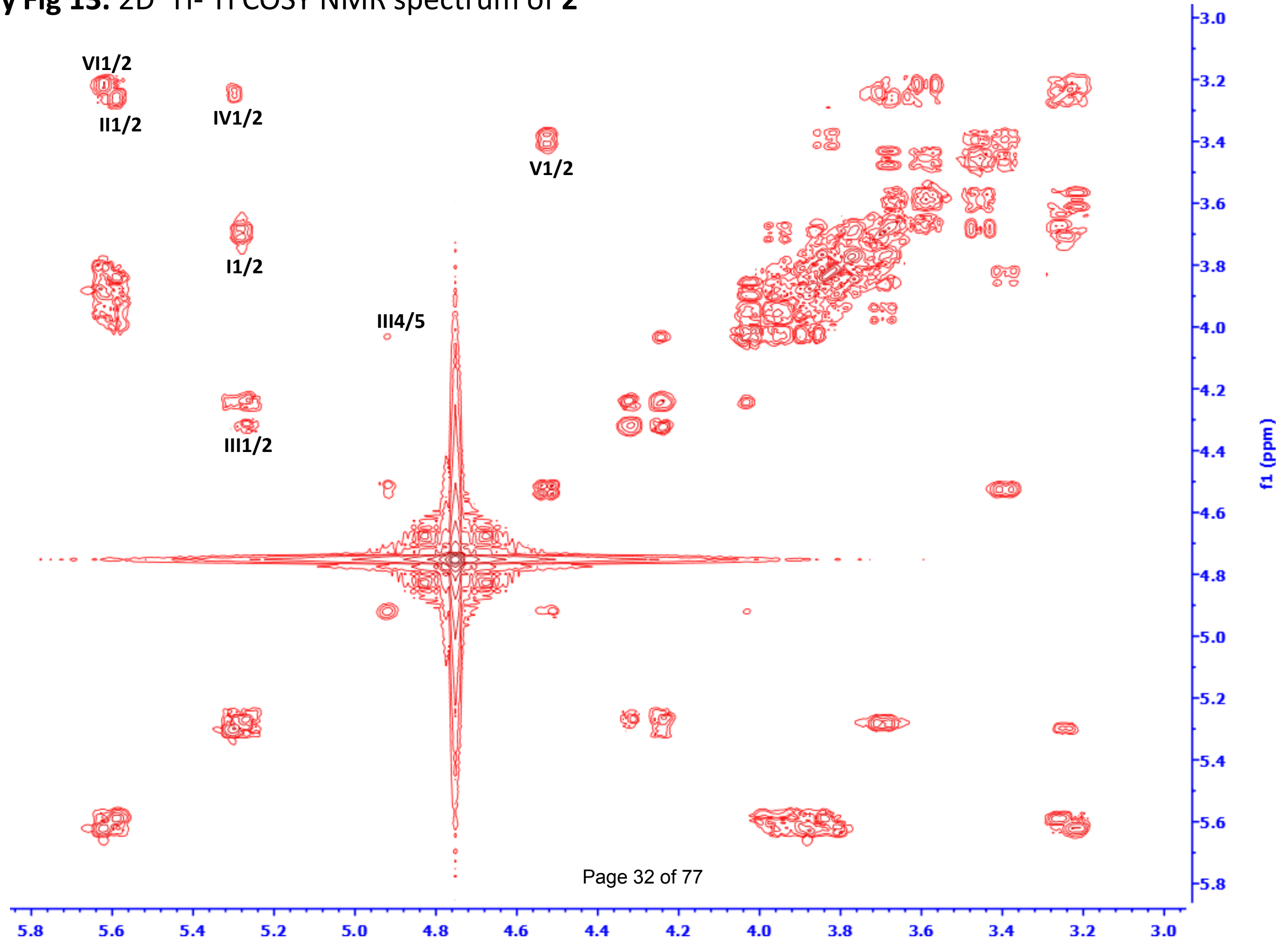

Supplementary Fig 14. 2D ^{1}H-^{13}C HSQC-TOCSY NMR spectrum of **2**

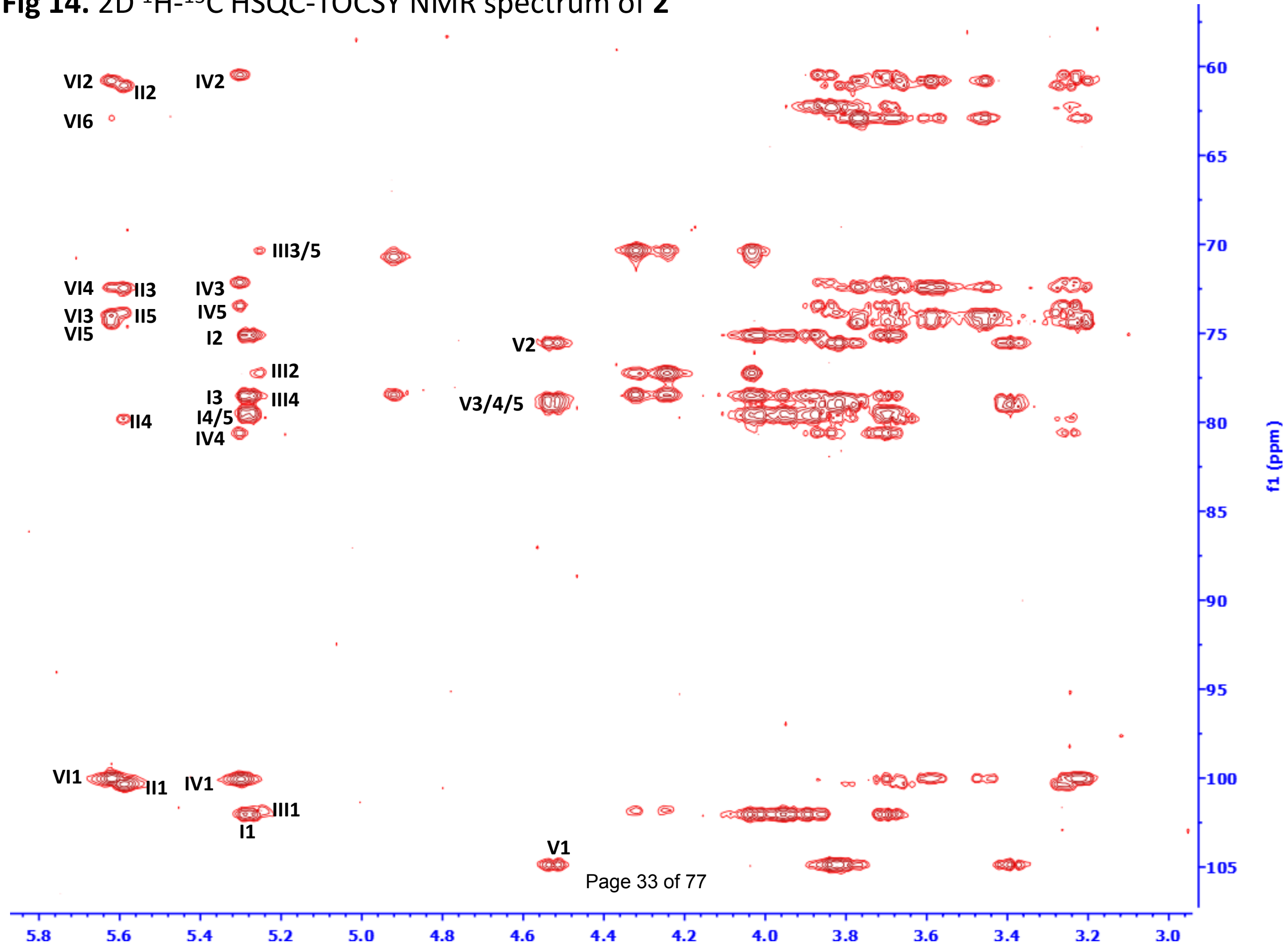

Supplementary Fig 15. 2D ^1H-^{13}C HMBC key correlations of **2**

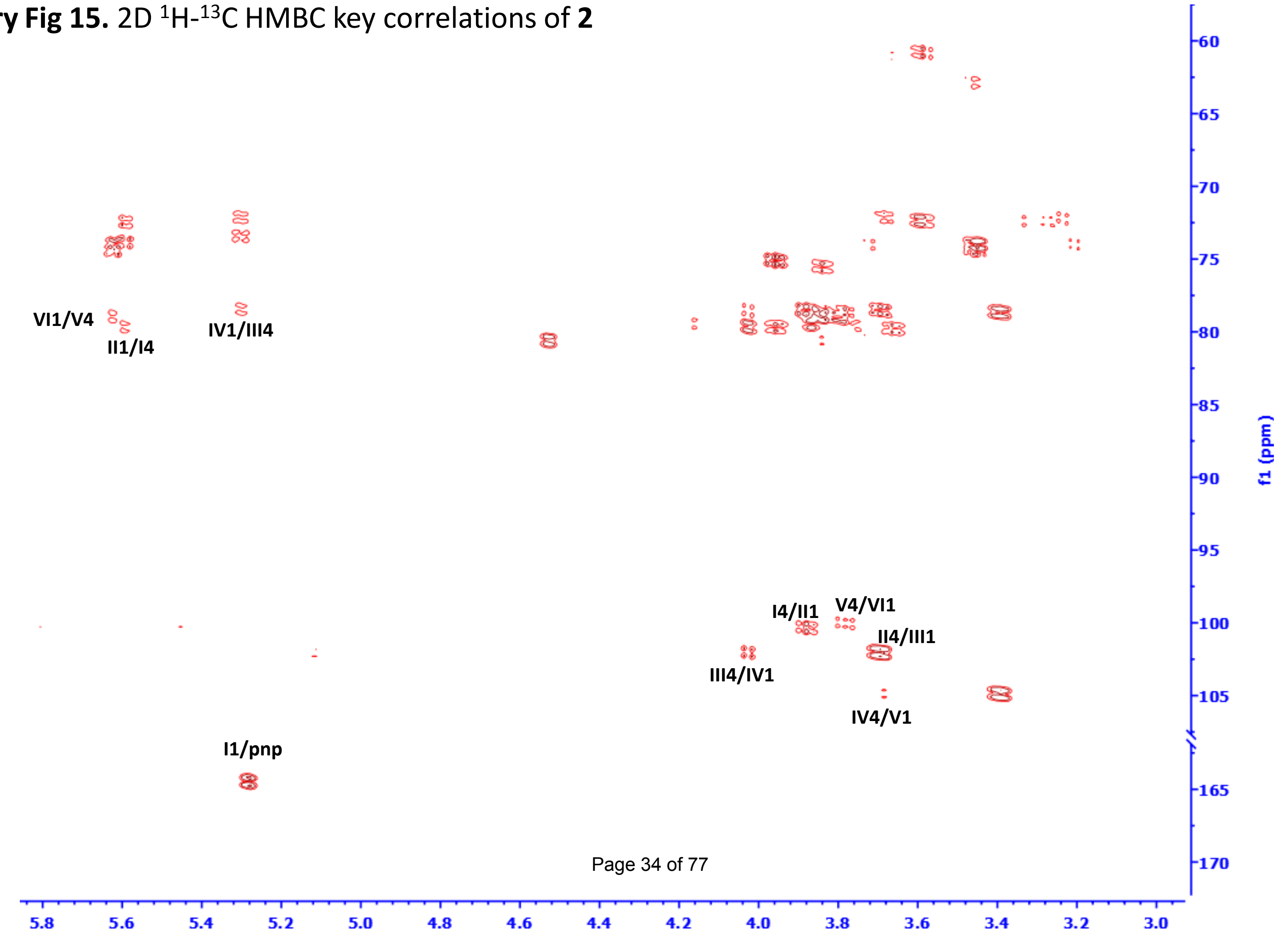

Supplementary Fig 16. 2D ^{1}H-^{1}H NOESY key correlations of **2**

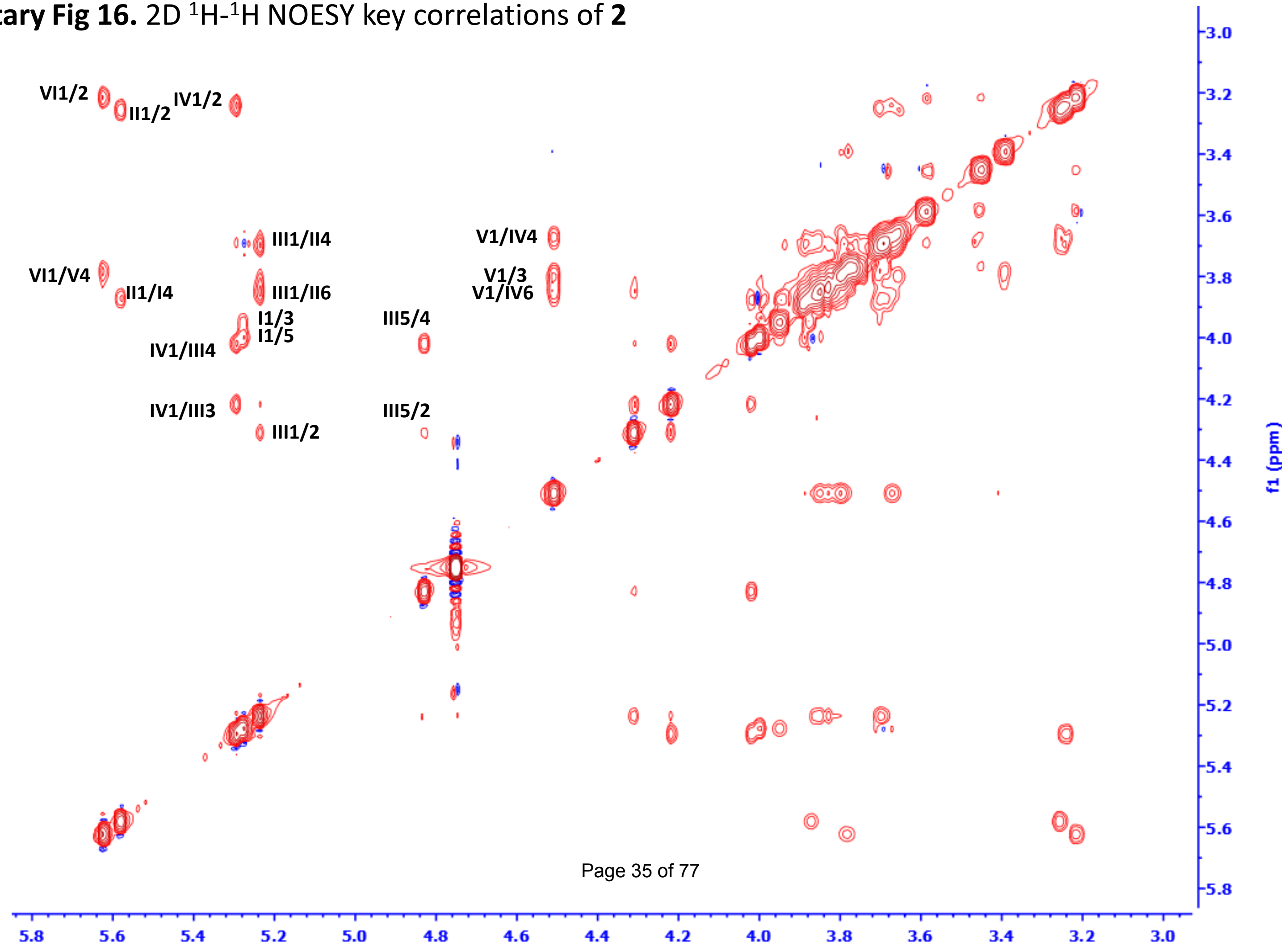

Supplementary Fig 17. ^{1}H-NMR spectrum of **3**

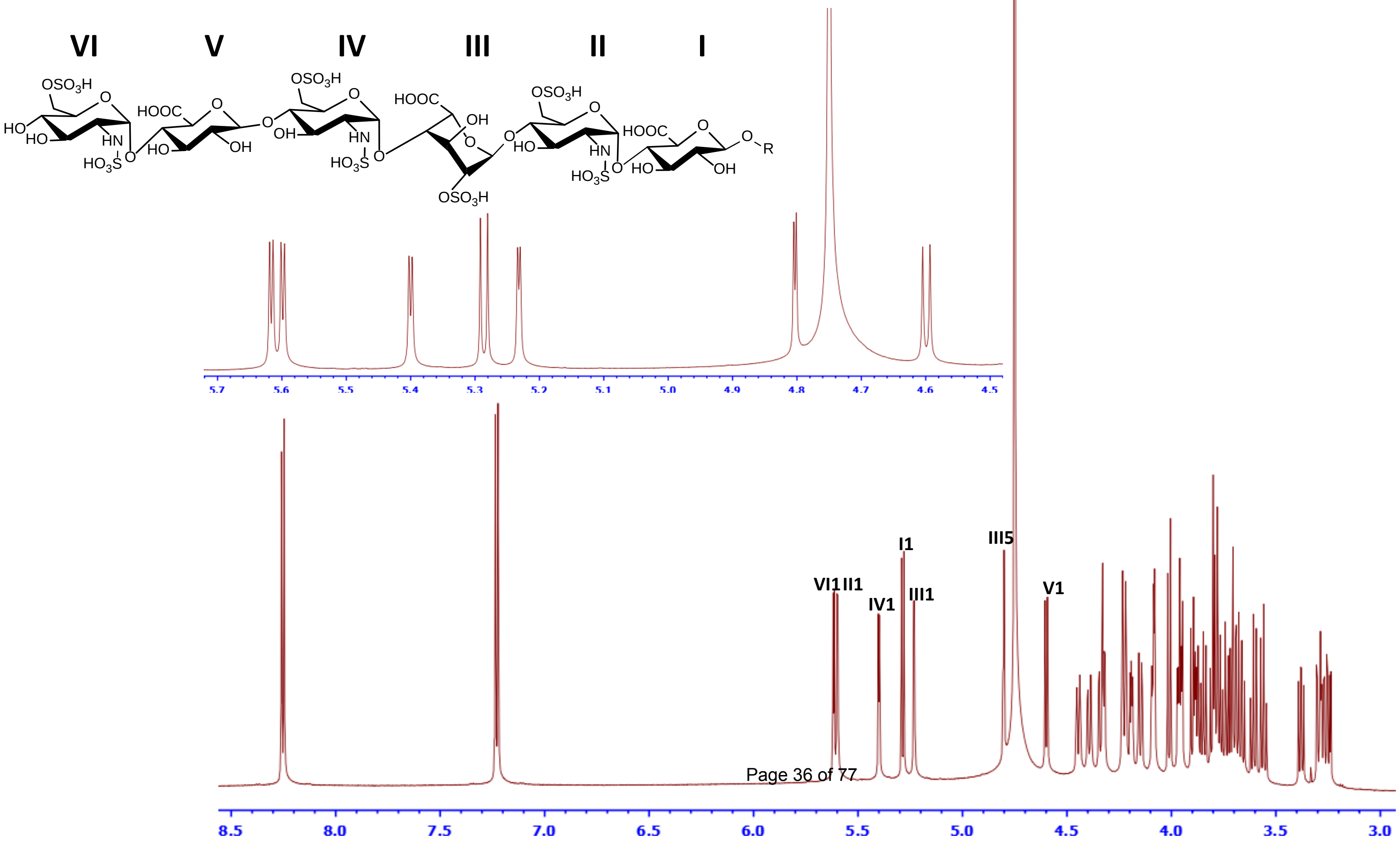

Supplementary Fig 18. ^{13}C-NMR spectrum of **3**

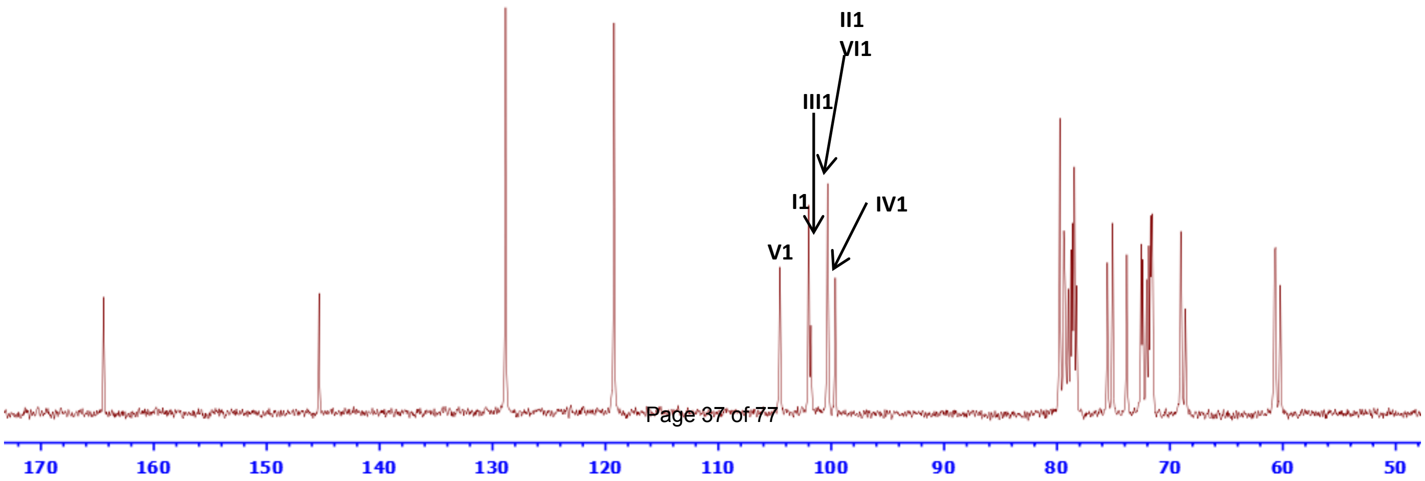

Supplementary Fig 19. 2D ^{1}H-^{13}C HSQC NMR spectrum of **3**

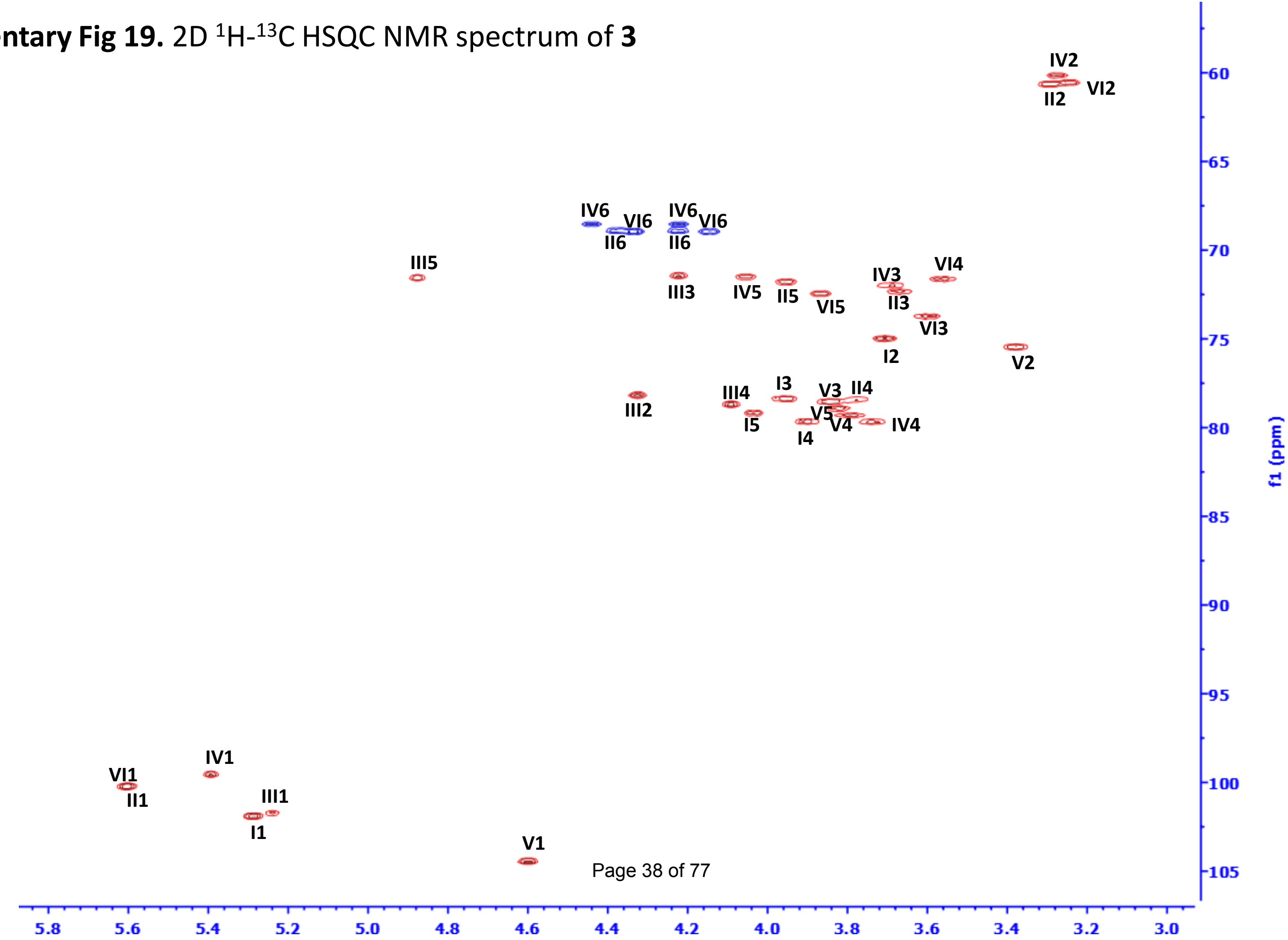

Supplementary Fig 20. 2D ^1H-^1H COSY NMR spectrum of **3**

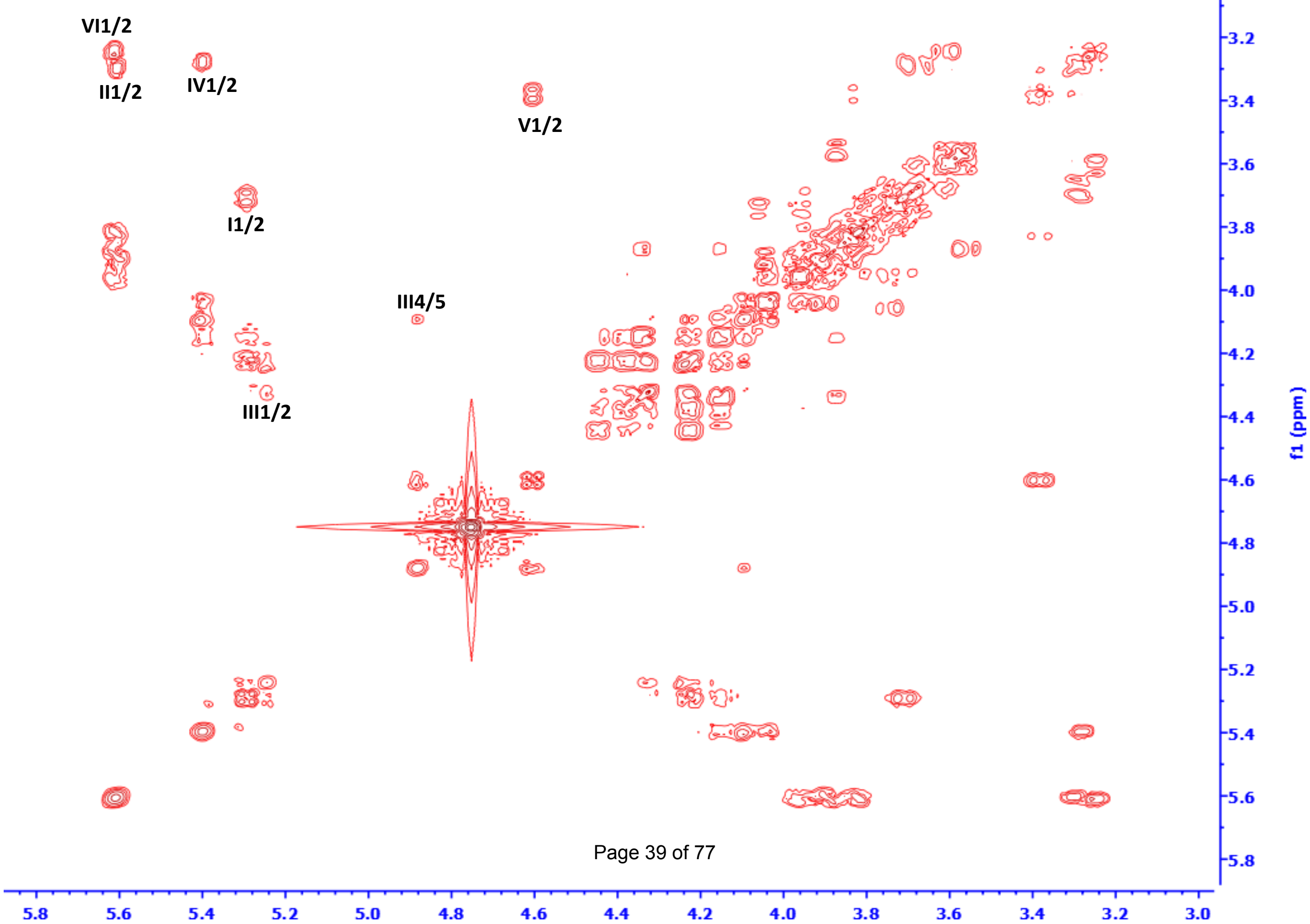

Supplementary Fig 21. 2D ^{1}H-^{13}C HSQC-TOCSY NMR spectrum of **3**

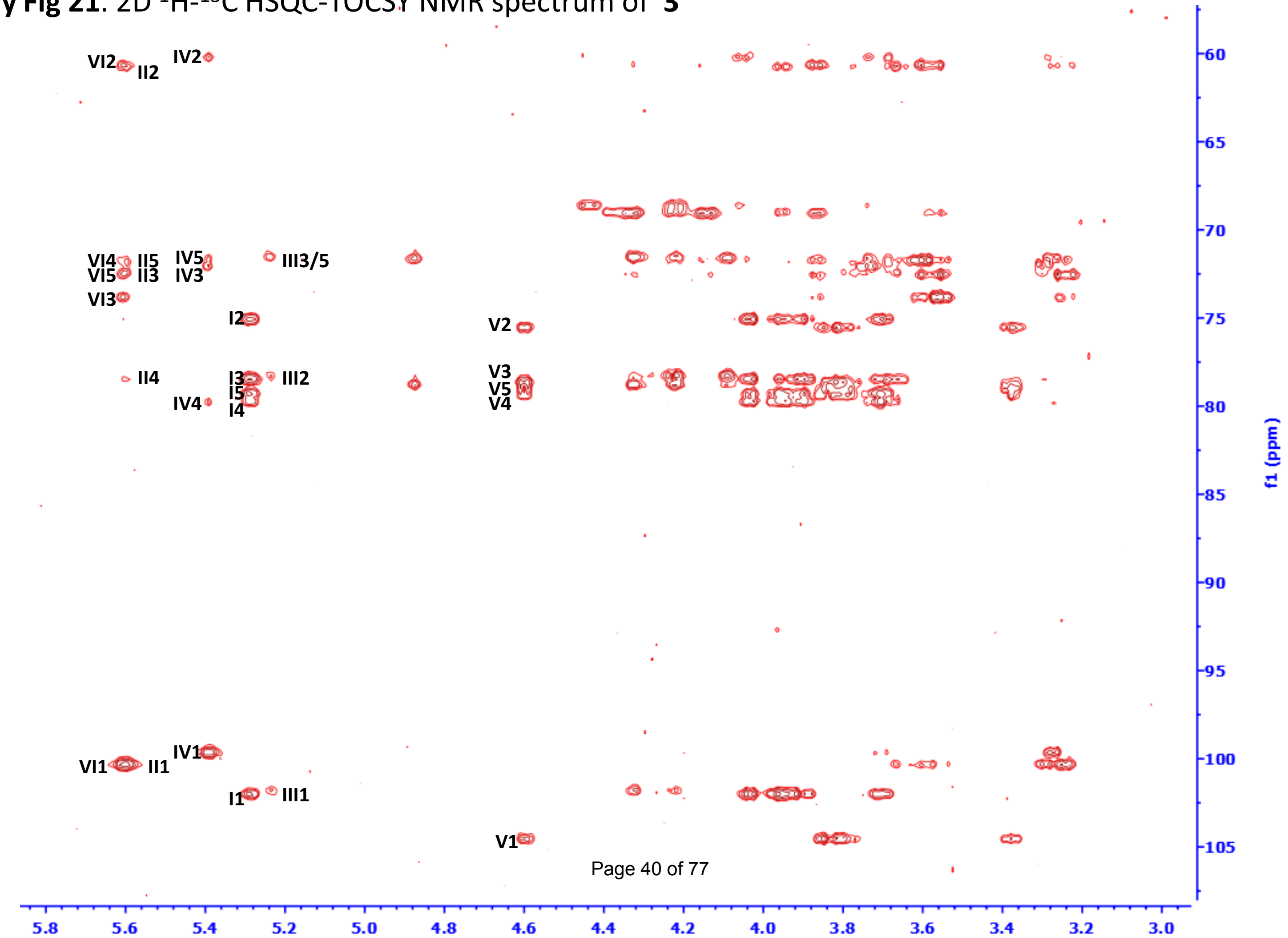

Supplementary Fig 22. 2D ^{1}H-^{13}C HMBC key correlations of **3**

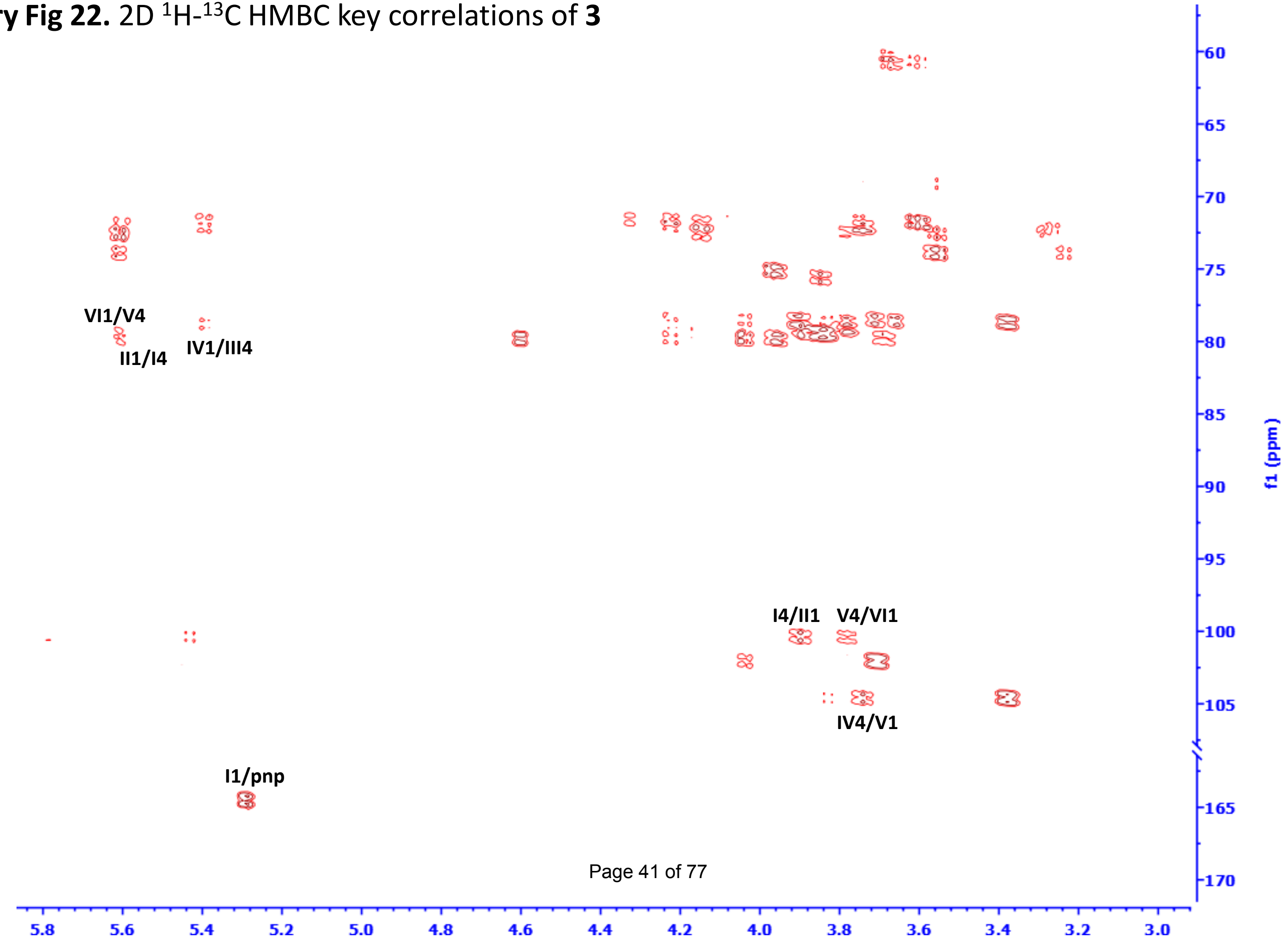

Supplementary Fig 23. 2D ^{1}H-^{1}H NOESY key correlations of **3**

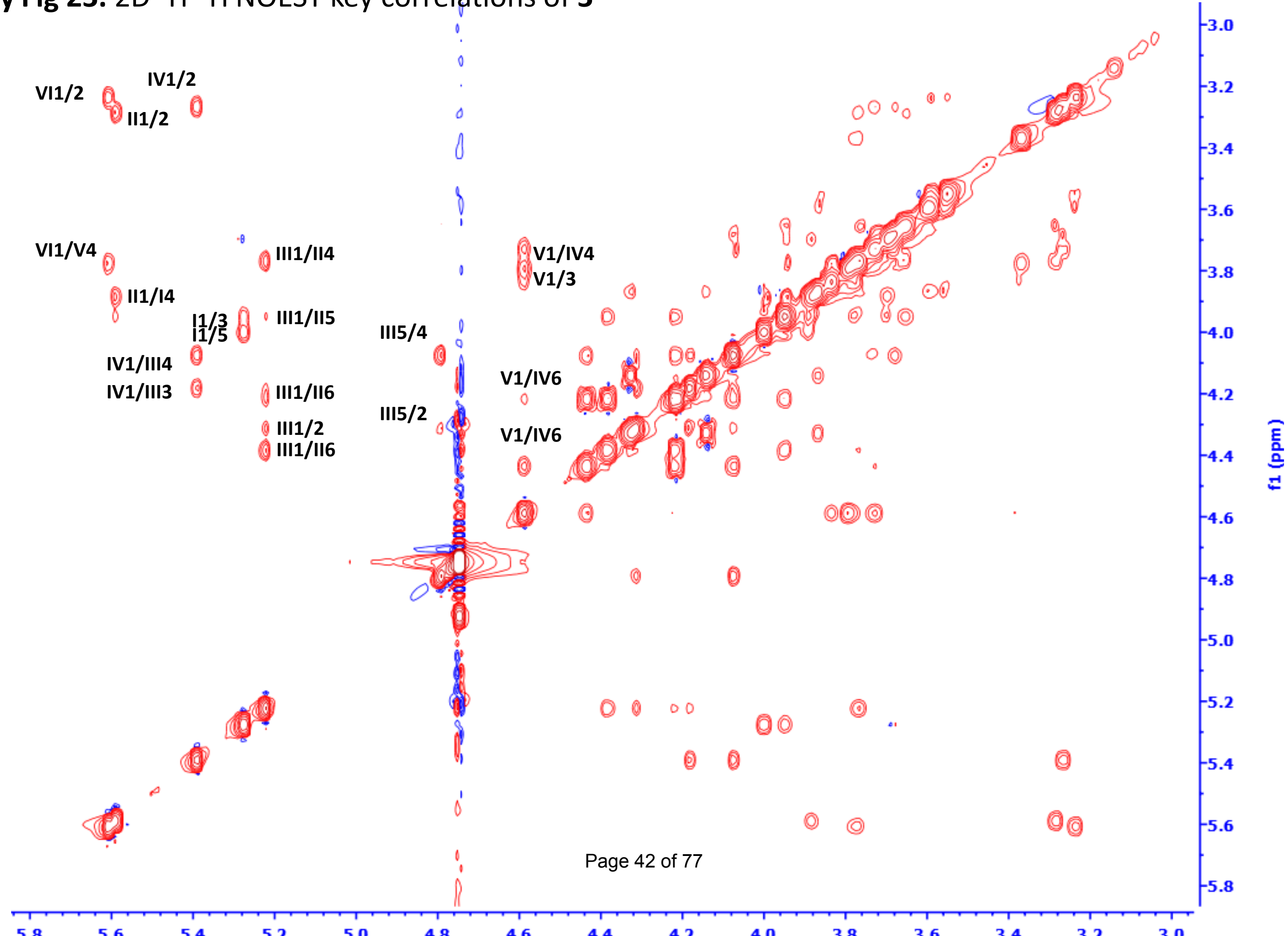

Supplementary Fig 24. ^{1}H-NMR spectrum of **4**

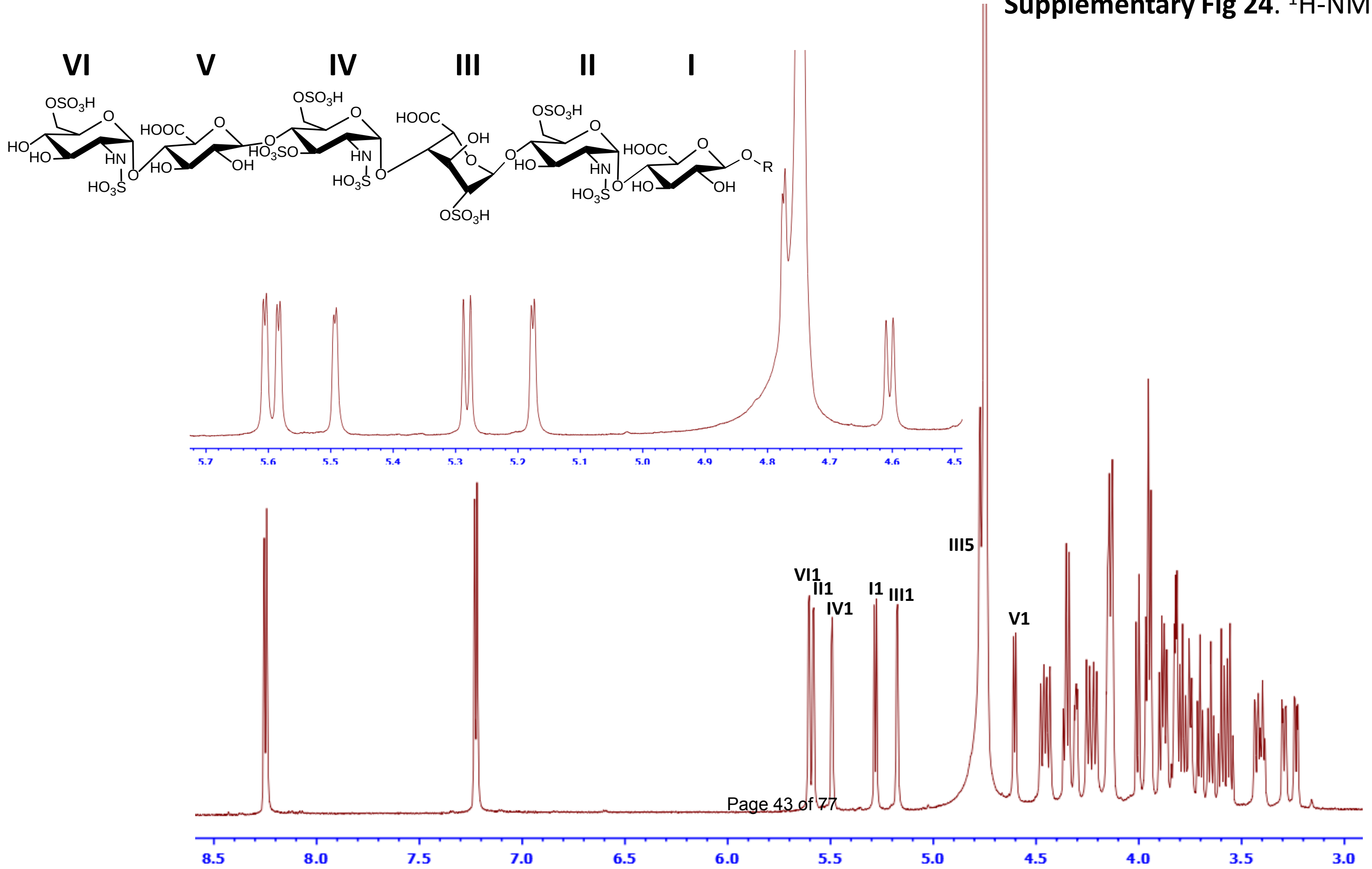

Supplementary Fig 25. ^{13}C-NMR spectrum of **4**

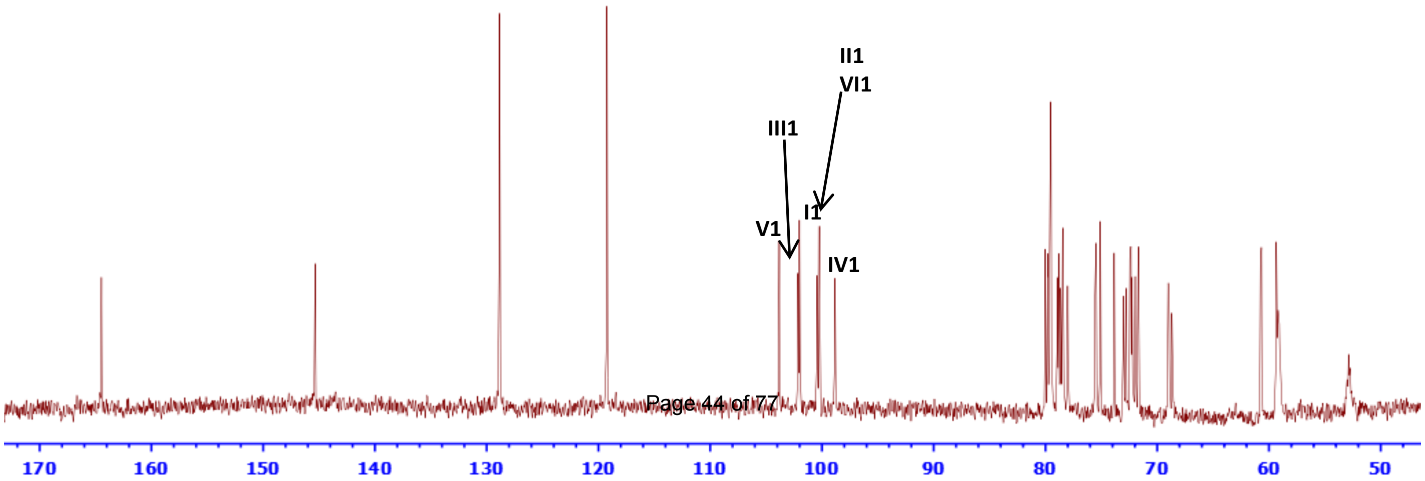

Supplementary Fig 26. 2D ^{1}H-^{13}C HSQC NMR spectrum of **4**

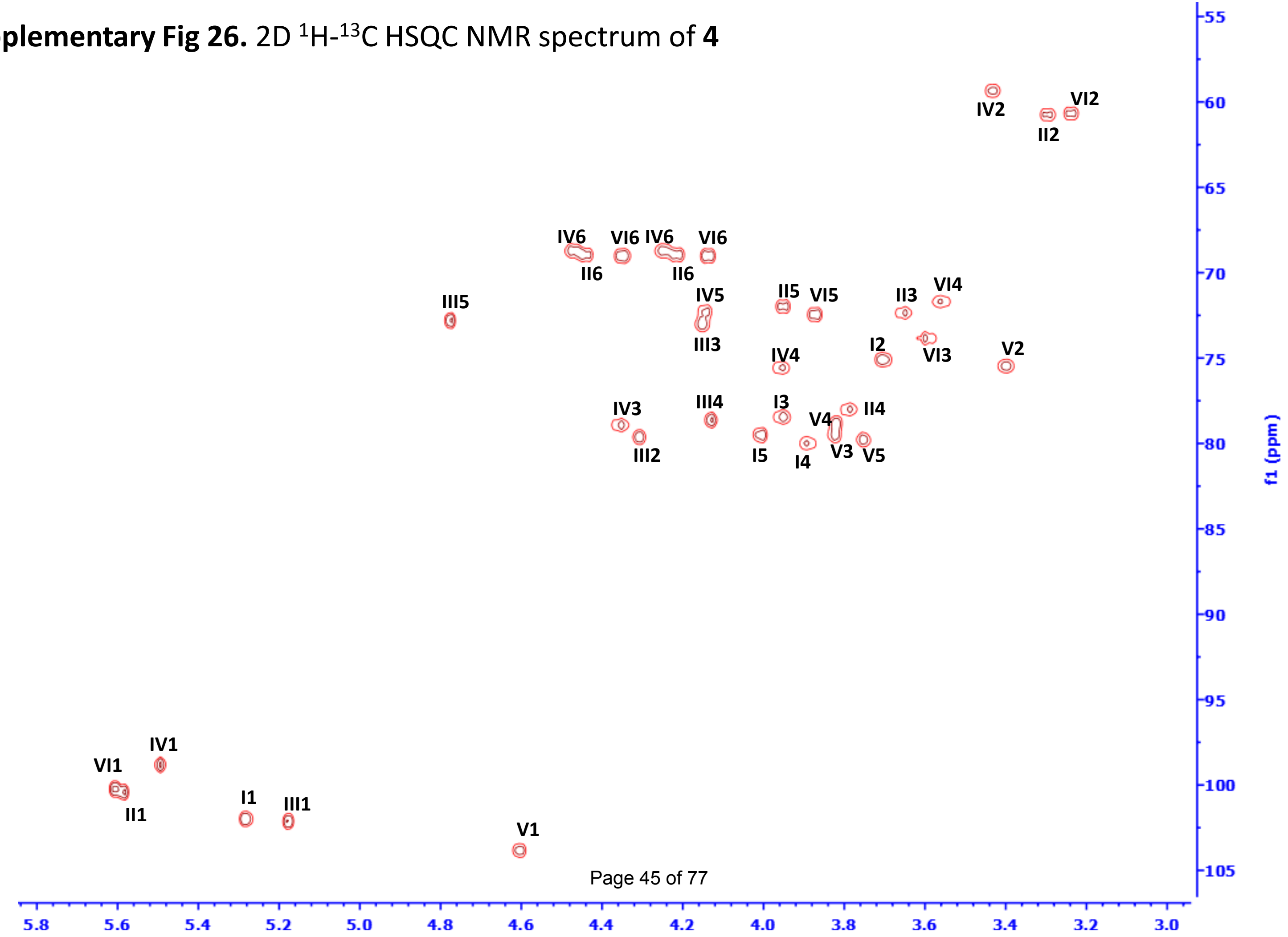

Supplementary Fig 27. 2D ^{1}H-^{1}H COSY NMR spectrum of **4**

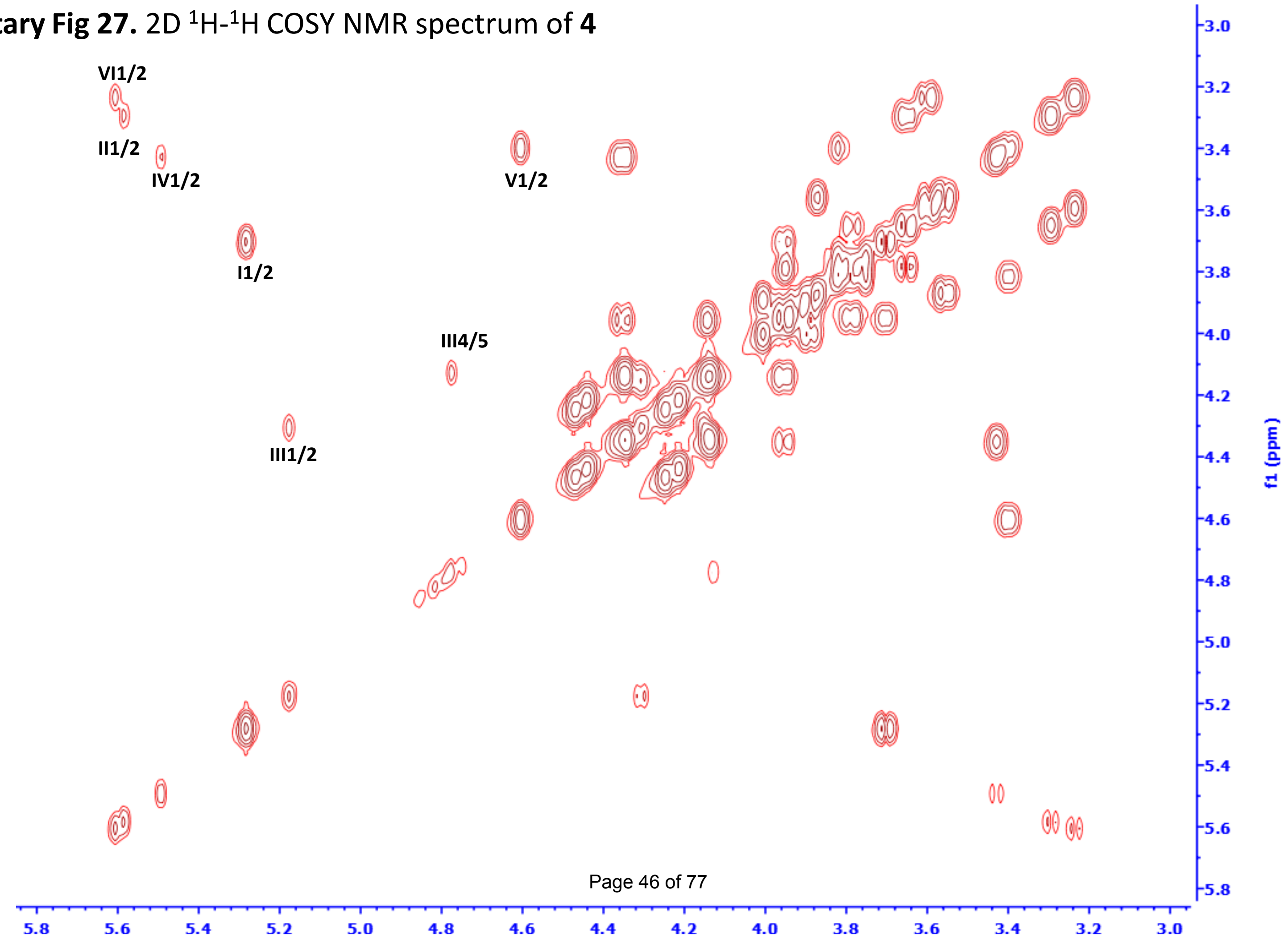

Supplementary Fig 28. 2D ^1H-^{13}C HSQC-TOCSY NMR spectrum of **4**

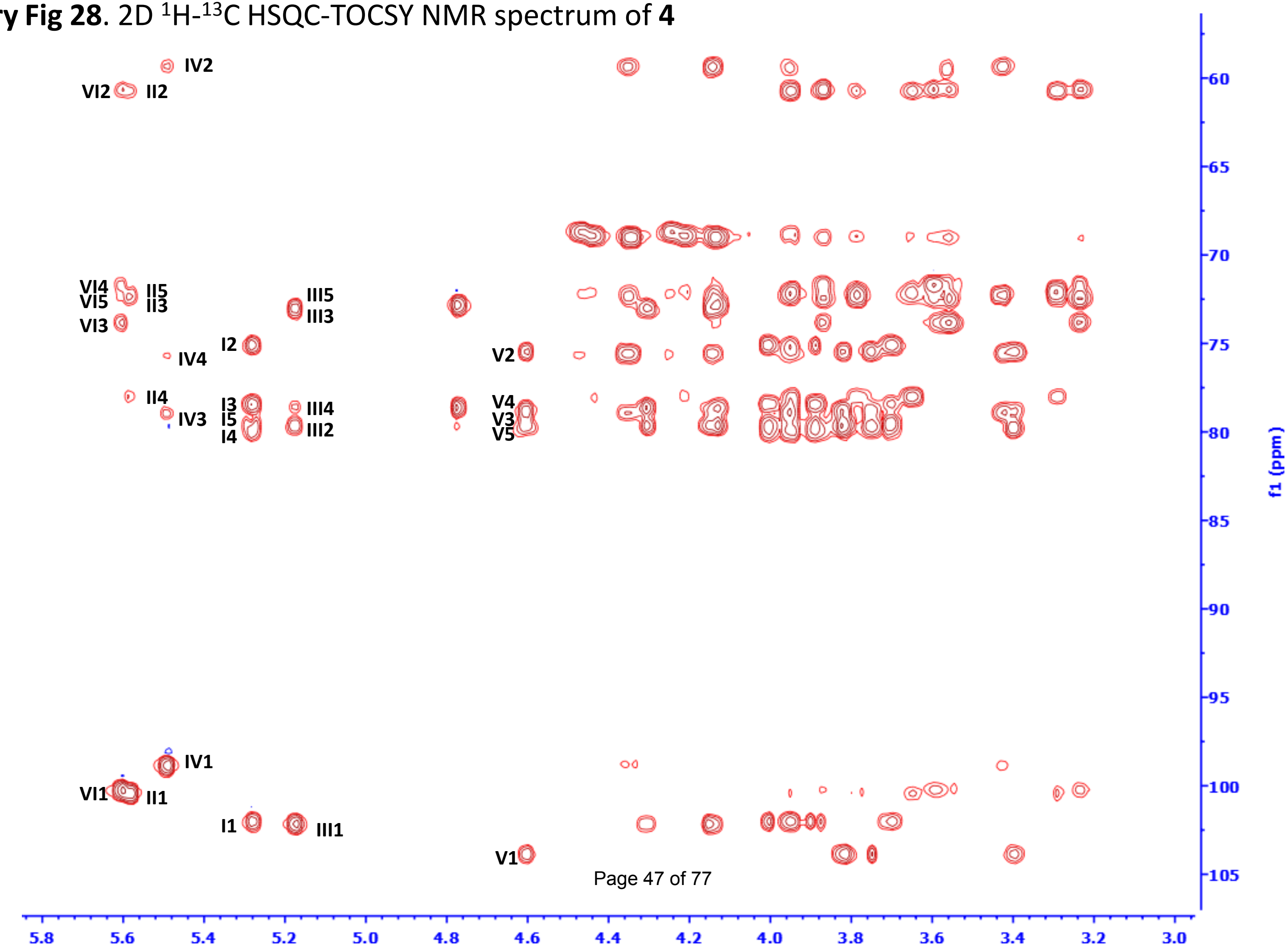

Supplementary Fig 29. 2D ^{1}H-^{13}C HMBC key correlations of **4**

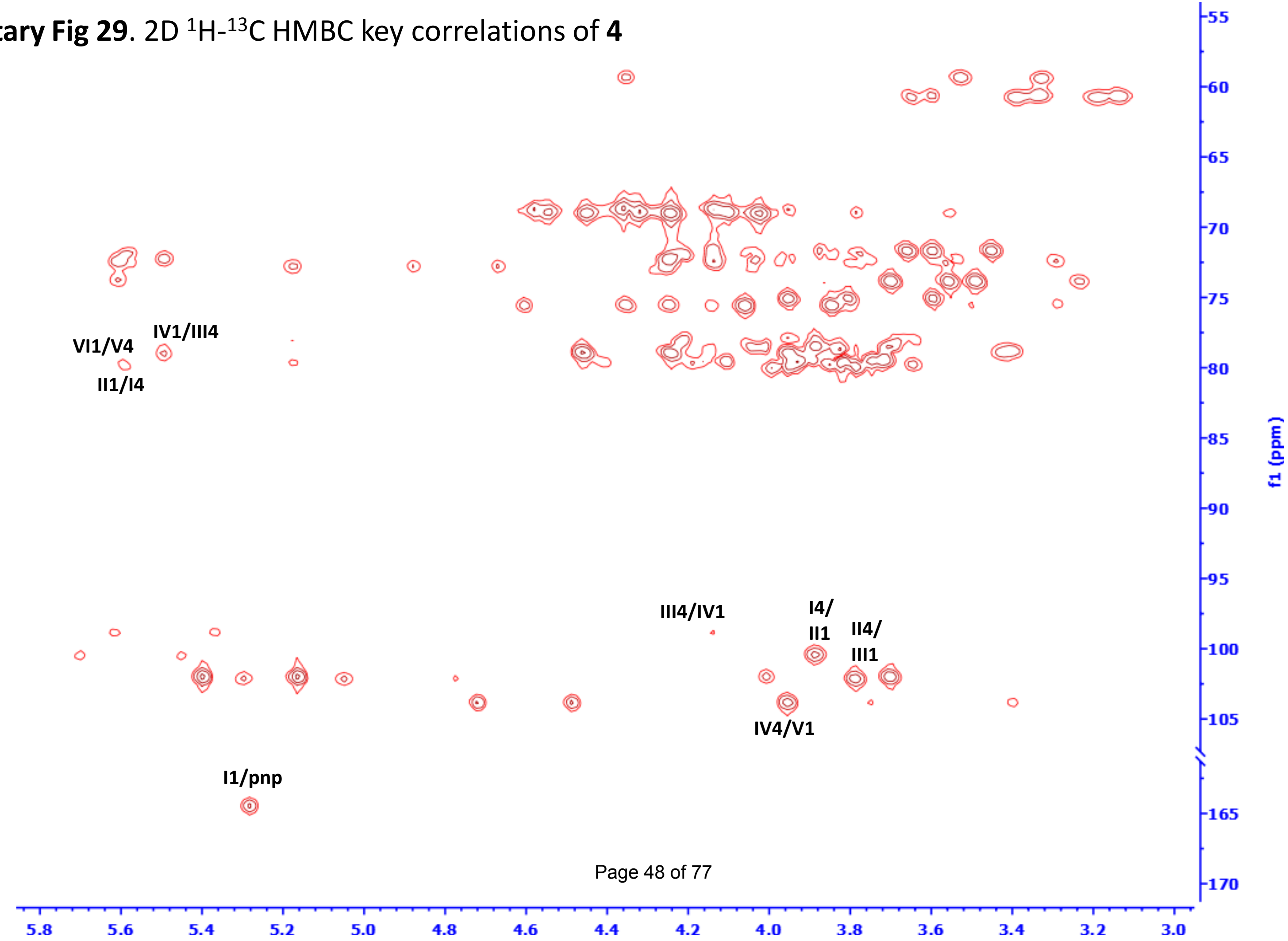

Supplementary Fig 30. 2D ^{1}H-^{1}H NOESY key correlations of **4**

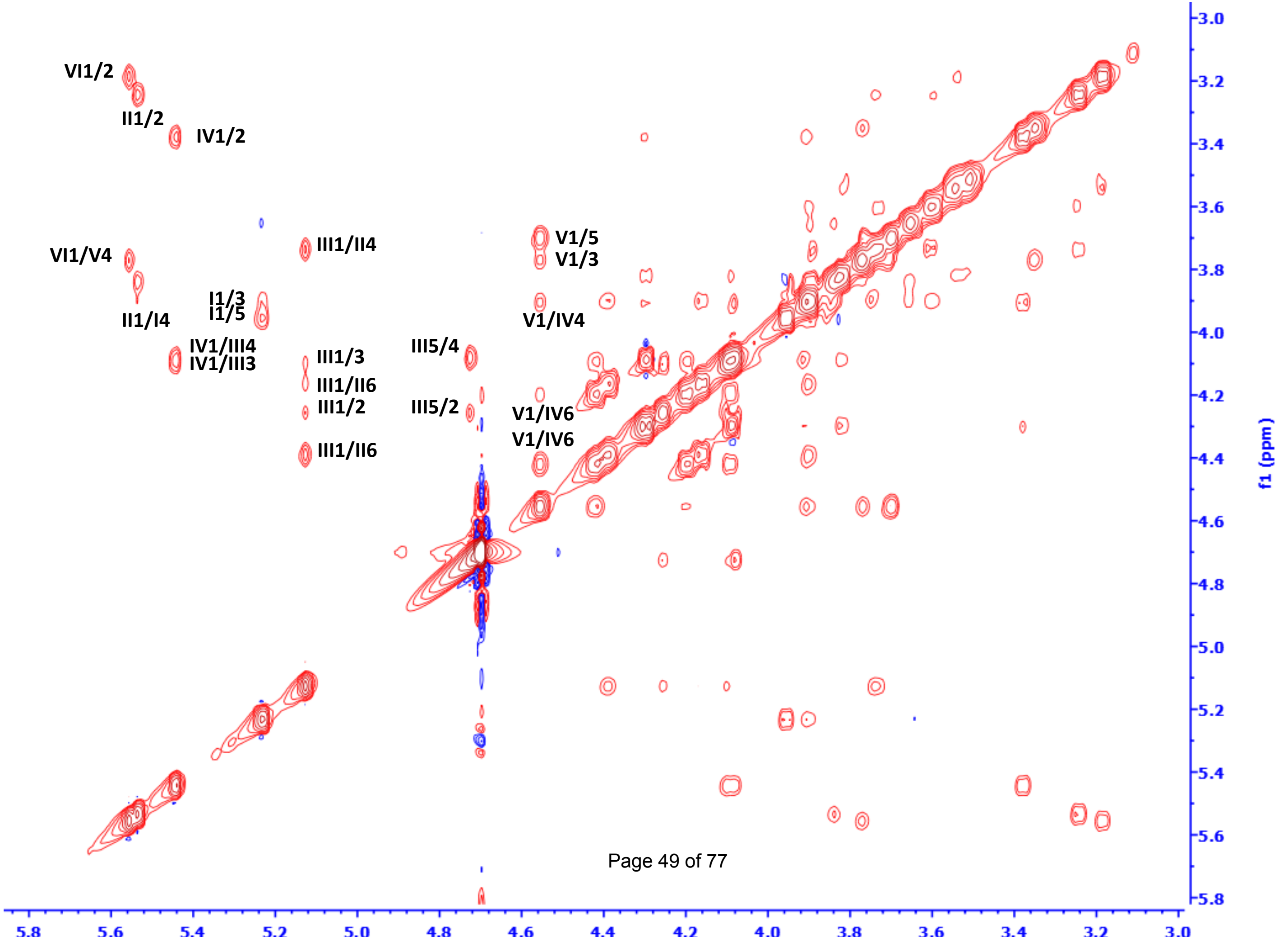

Supplementary Fig 31. ^{1}H-NMR spectrum of **5**

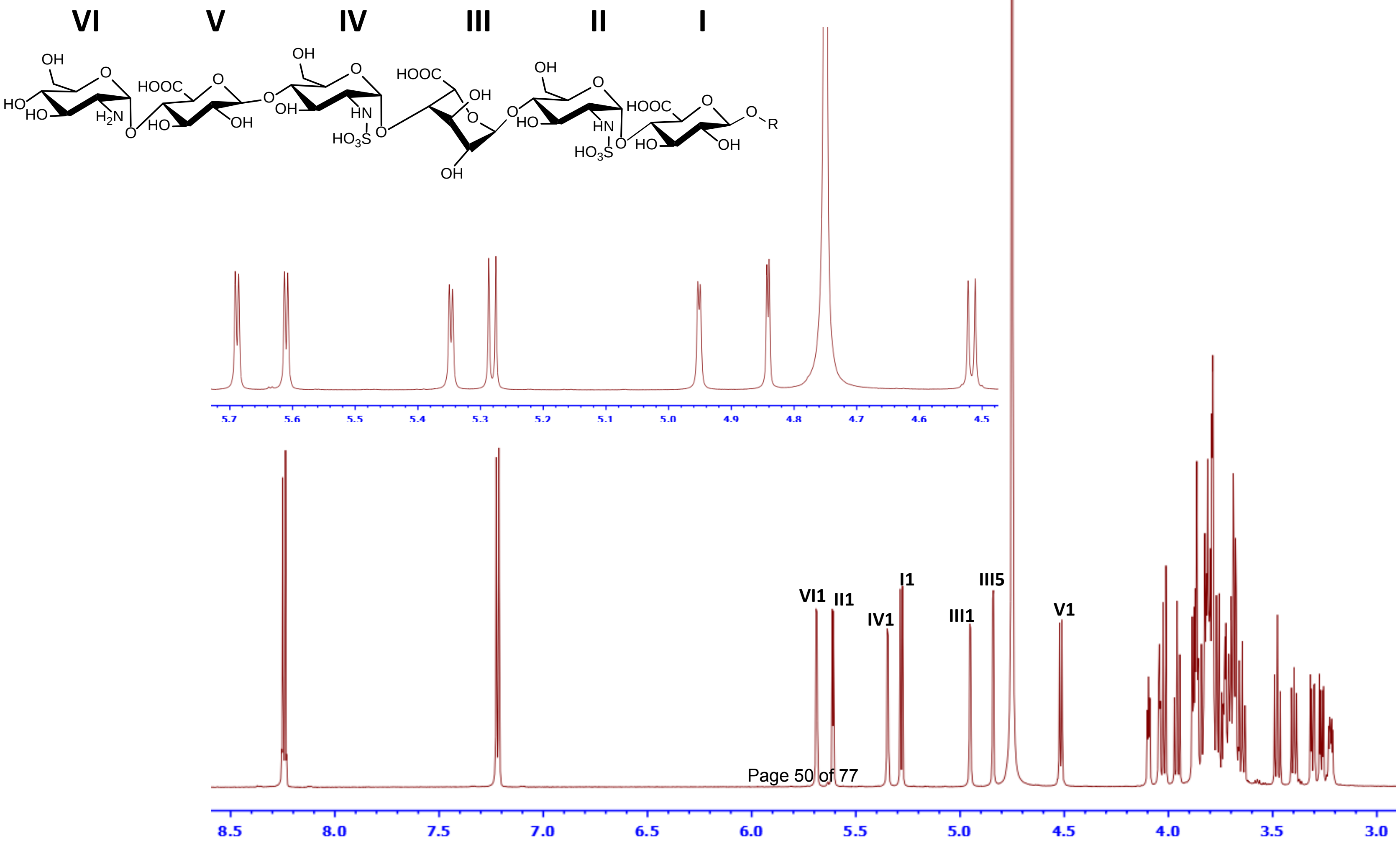

Supplementary Fig 32. ^{13}C-NMR spectrum of **5**

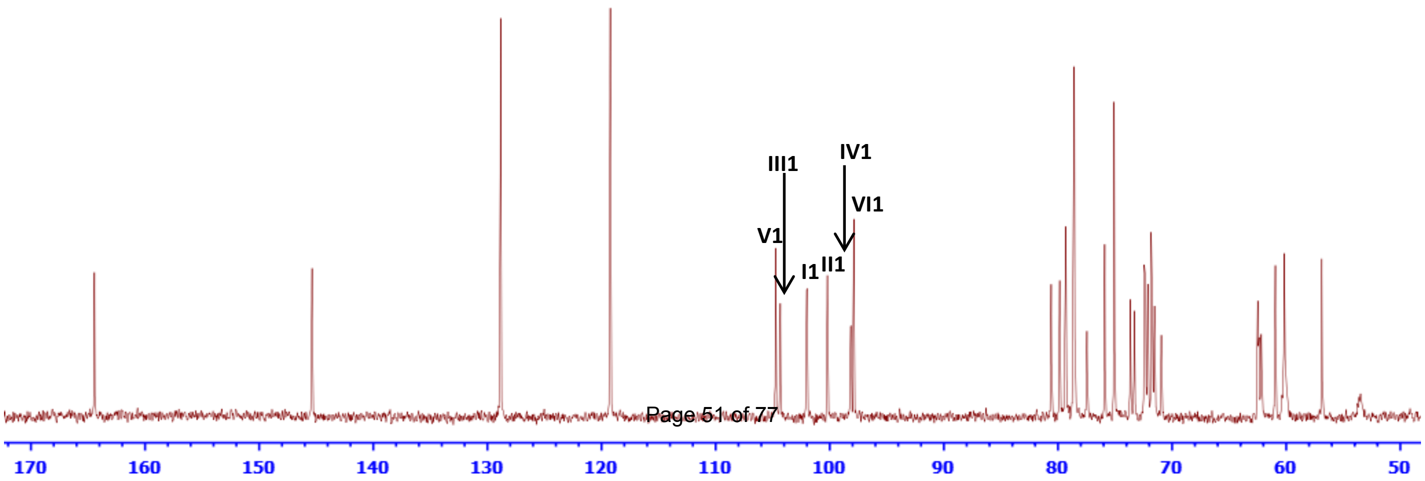

Supplementary Fig 33. 2D ^{1}H-^{13}C HSQC NMR spectrum of **5**

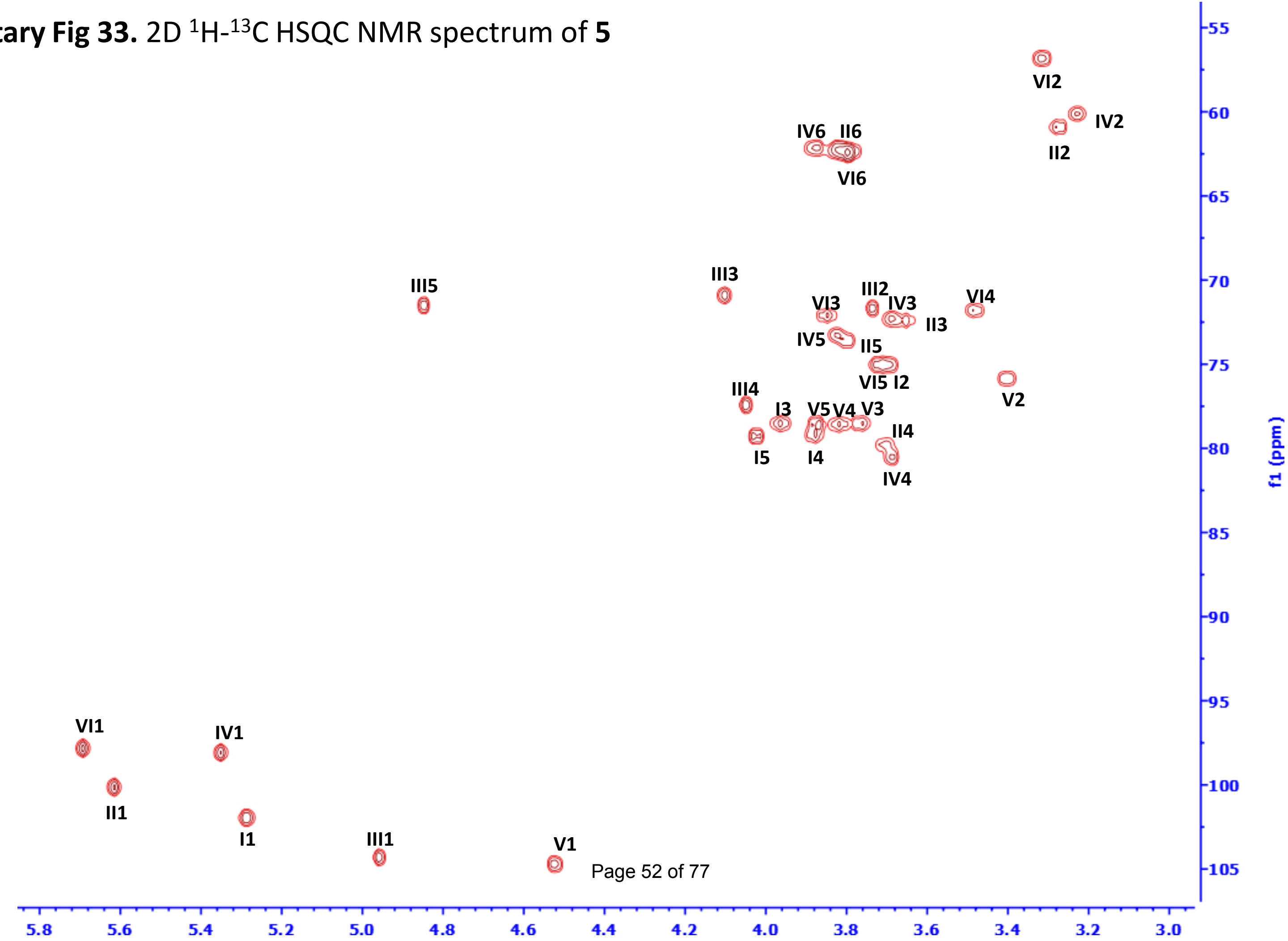

Supplementary Fig 34. 2D ^{1}H-^{1}H COSY NMR spectrum of **5**

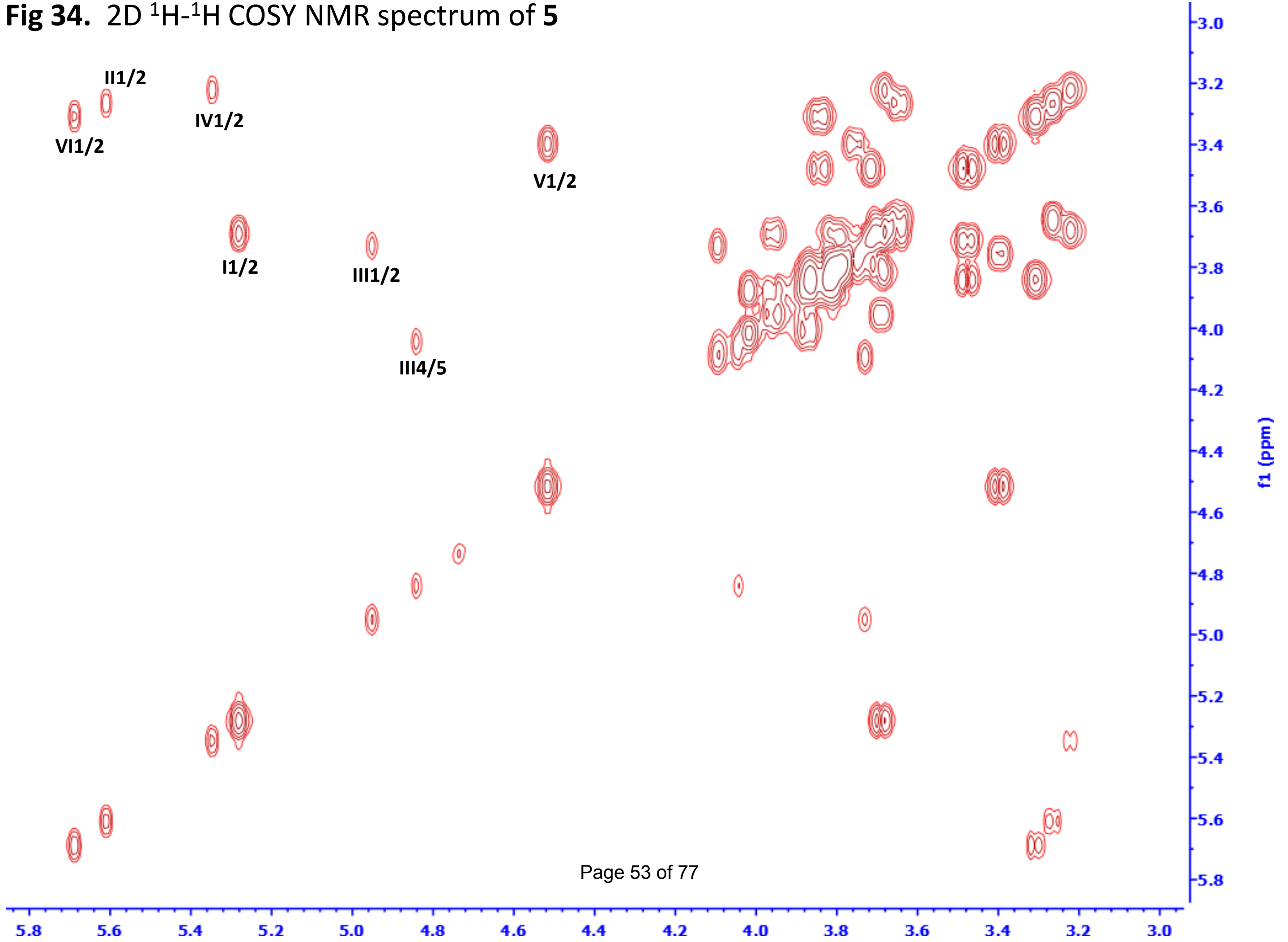

Supplementary Fig 35. 2D ^1H-^{13}C HSQC-TOCSY NMR spectrum of **5**

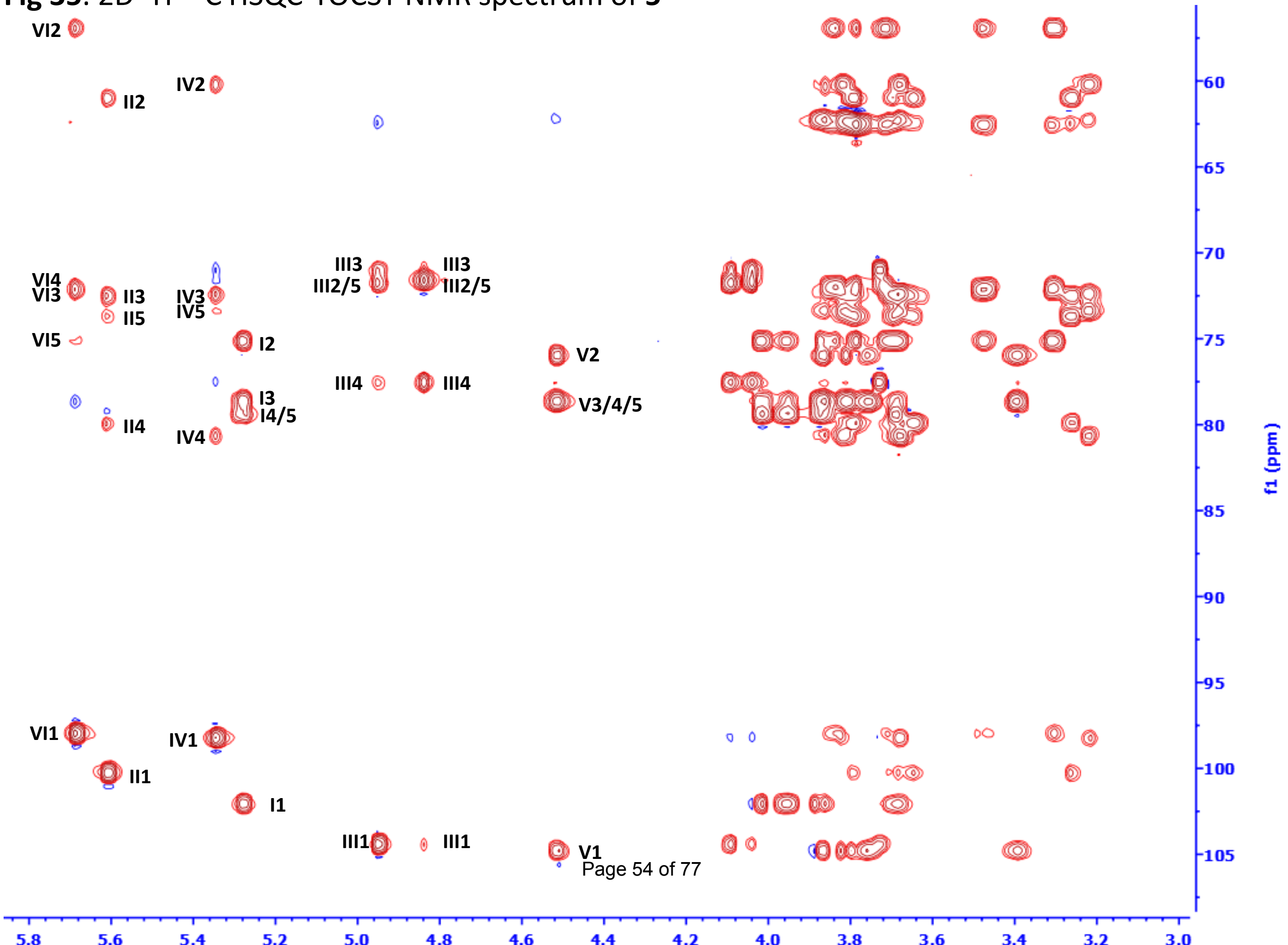

Supplementary Fig 36. 2D ^{1}H-^{13}C HMBC key correlations of **5**

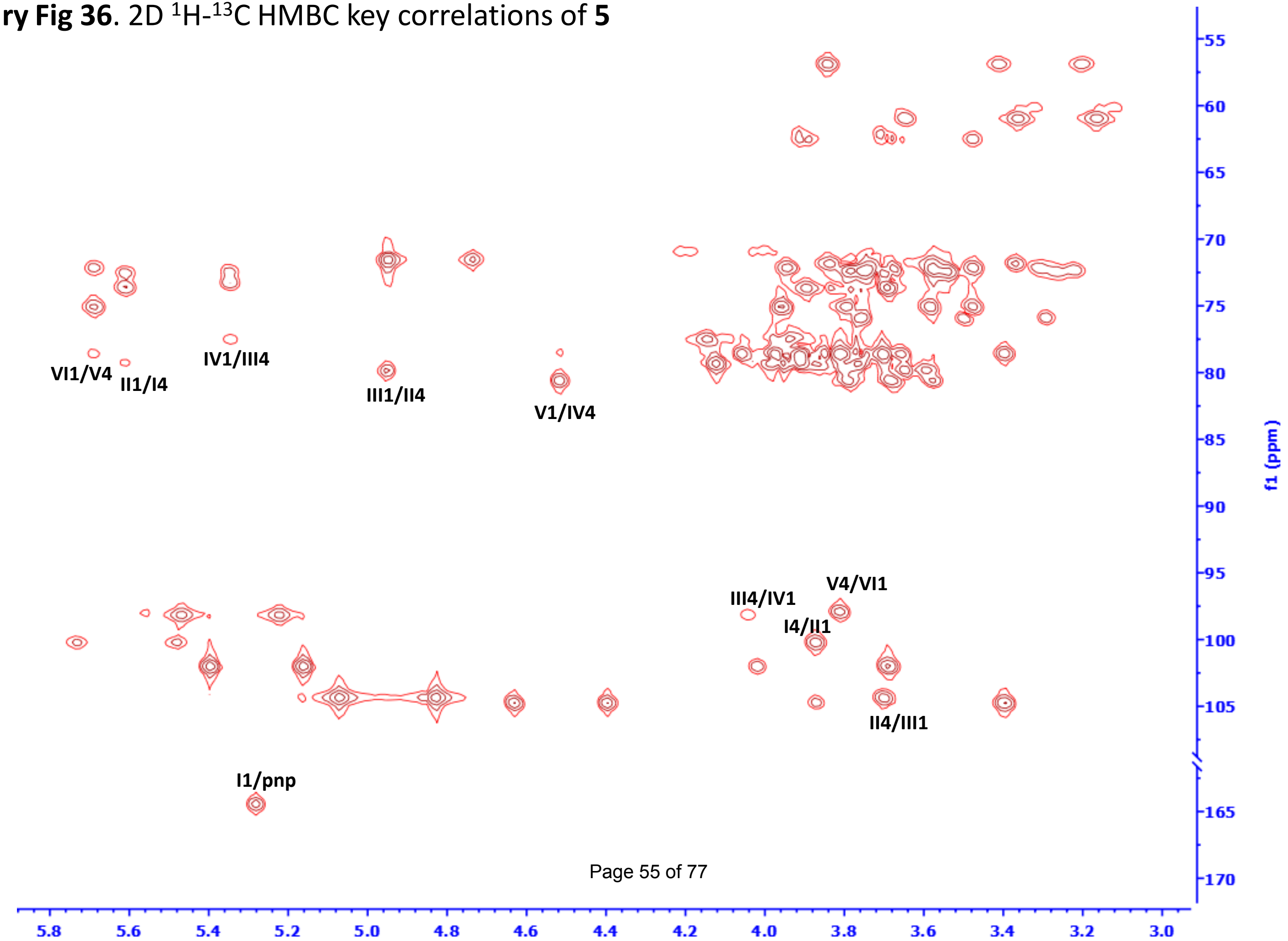

Supplementary Fig 37. 2D ^{1}H-^{1}H NOESY key correlations of **5**

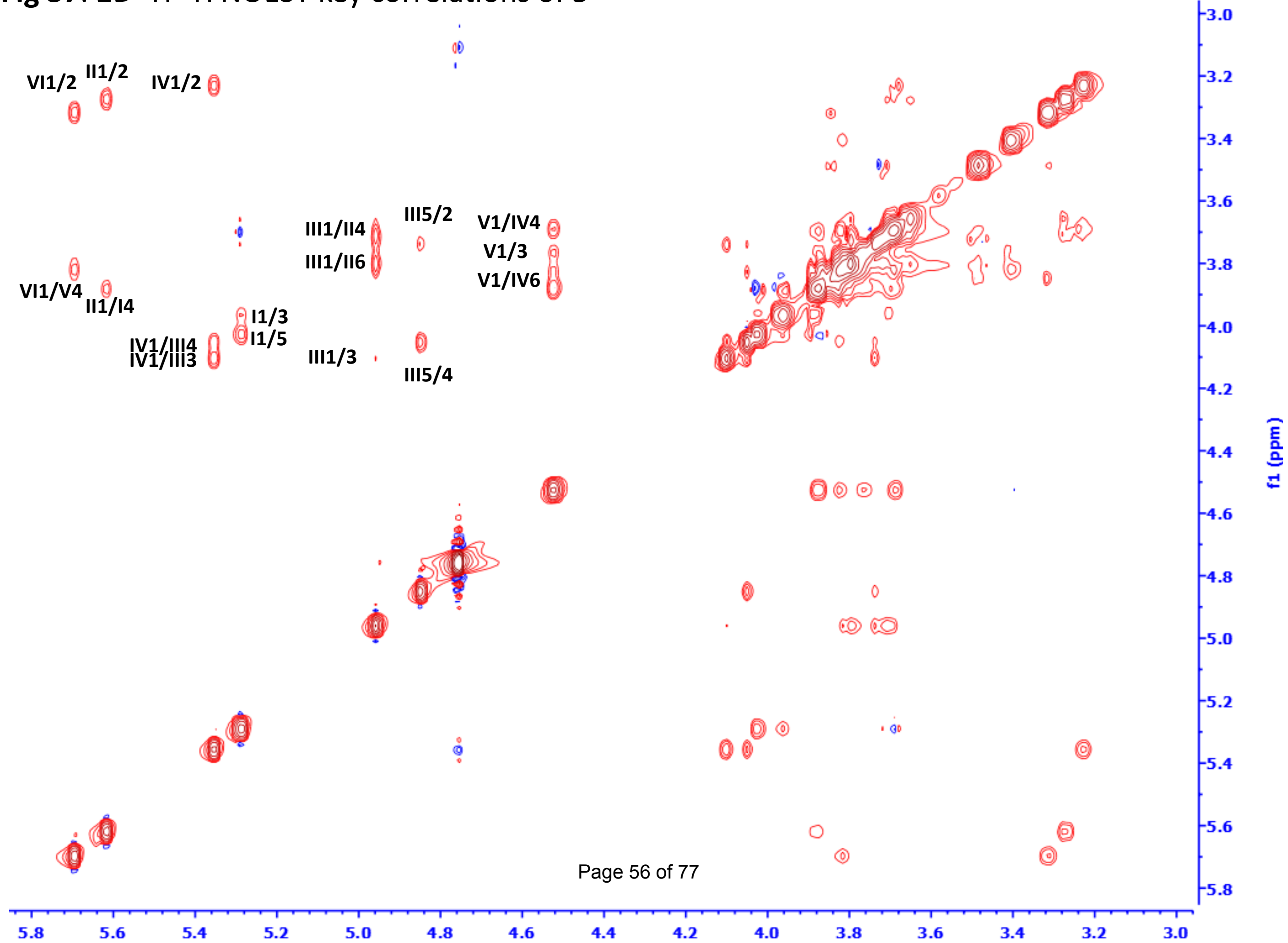

Supplementary Fig 38. ^{1}H-NMR spectrum of **6**

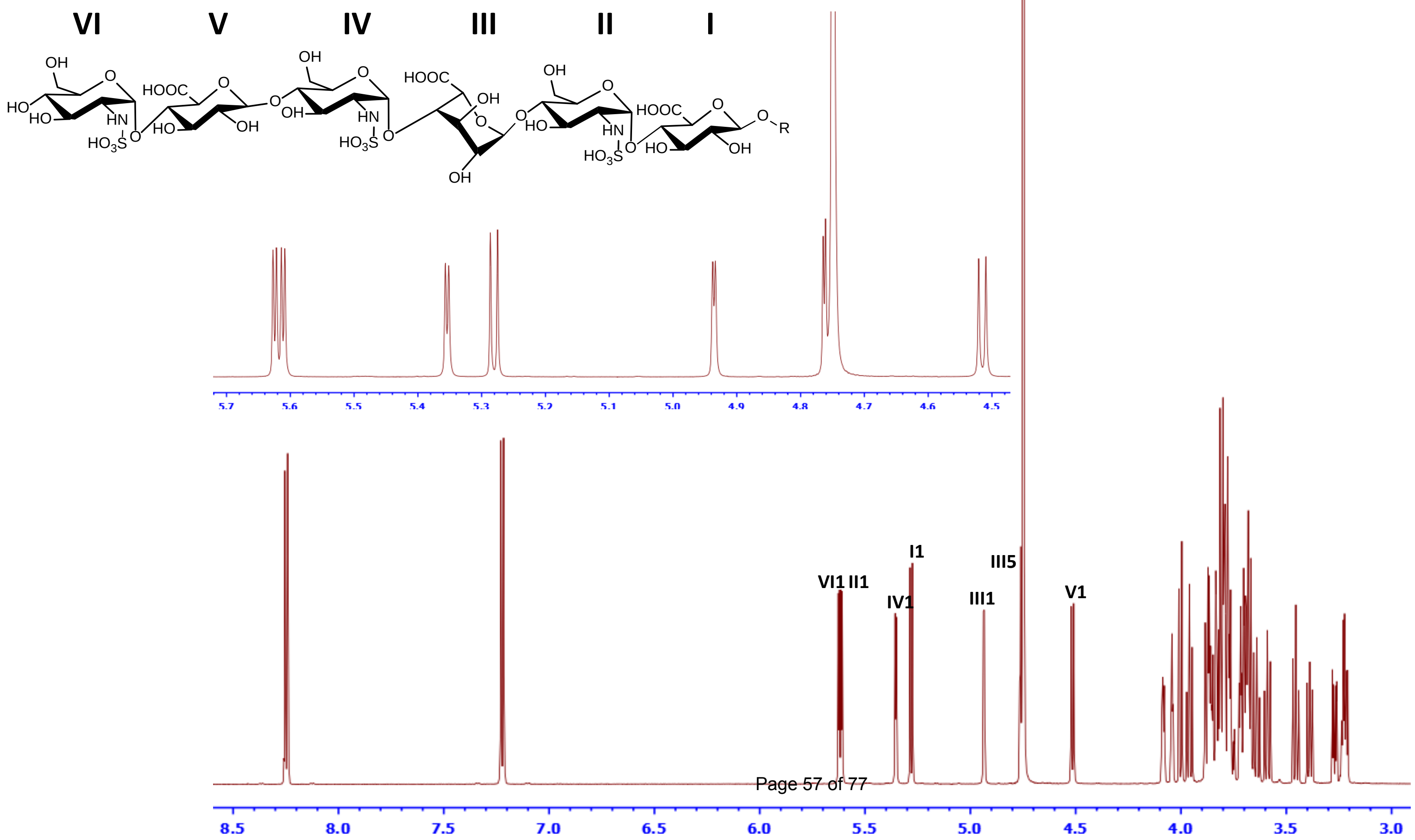

Supplementary Fig 39. ^{13}C-NMR spectrum of **6**

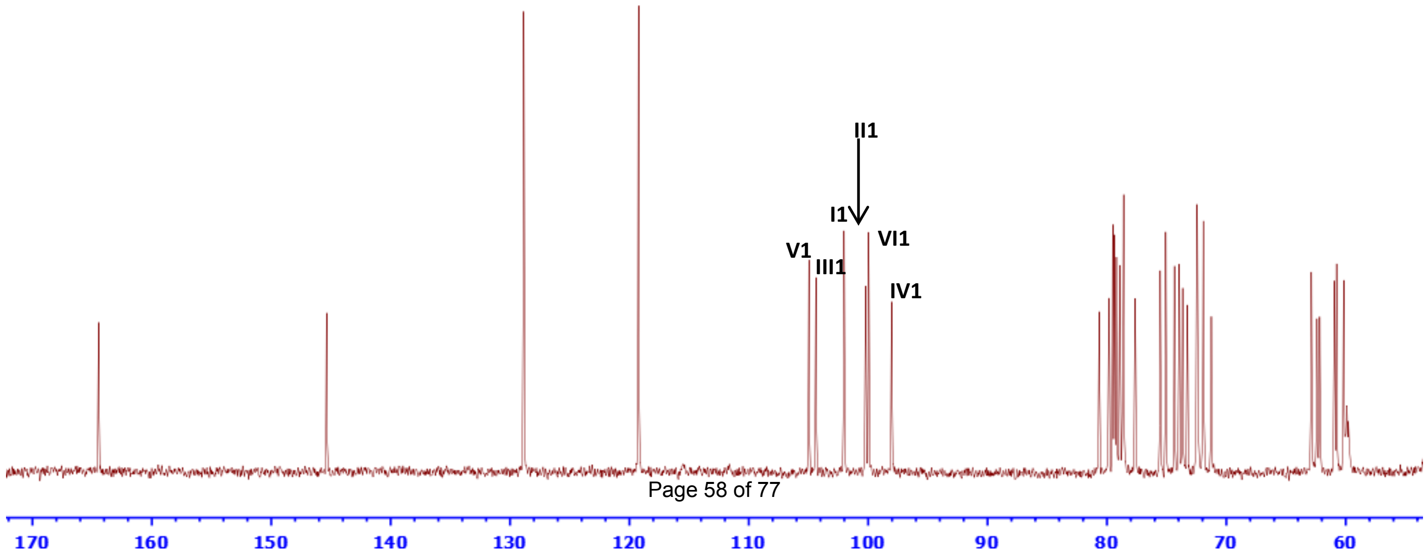

Supplementary Fig 40. 2D ^{1}H-^{13}C HSQC NMR spectrum of **6**

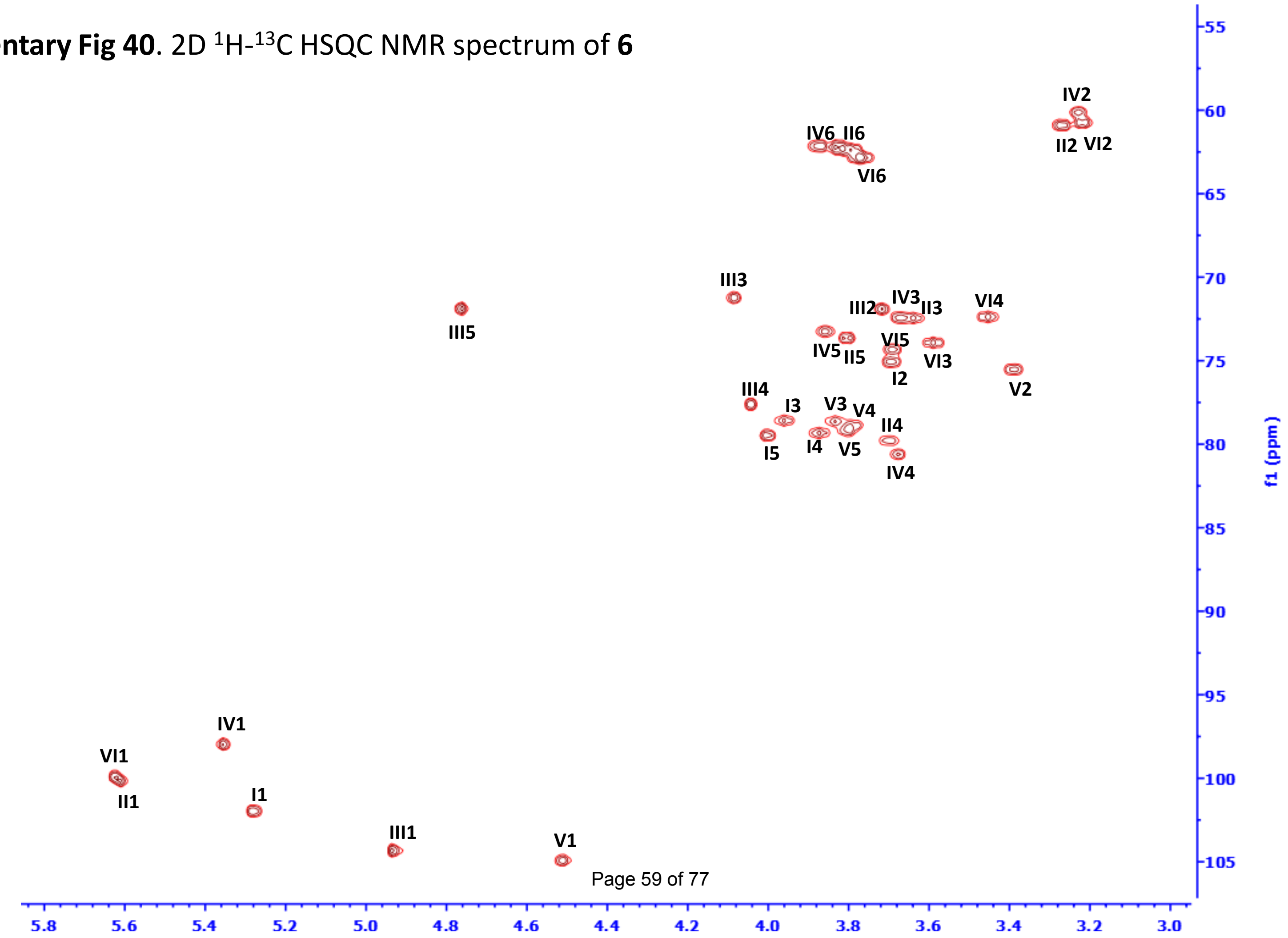

Supplementary Fig 41. 2D ^{1}H-^{1}H COSY NMR spectrum of **6**

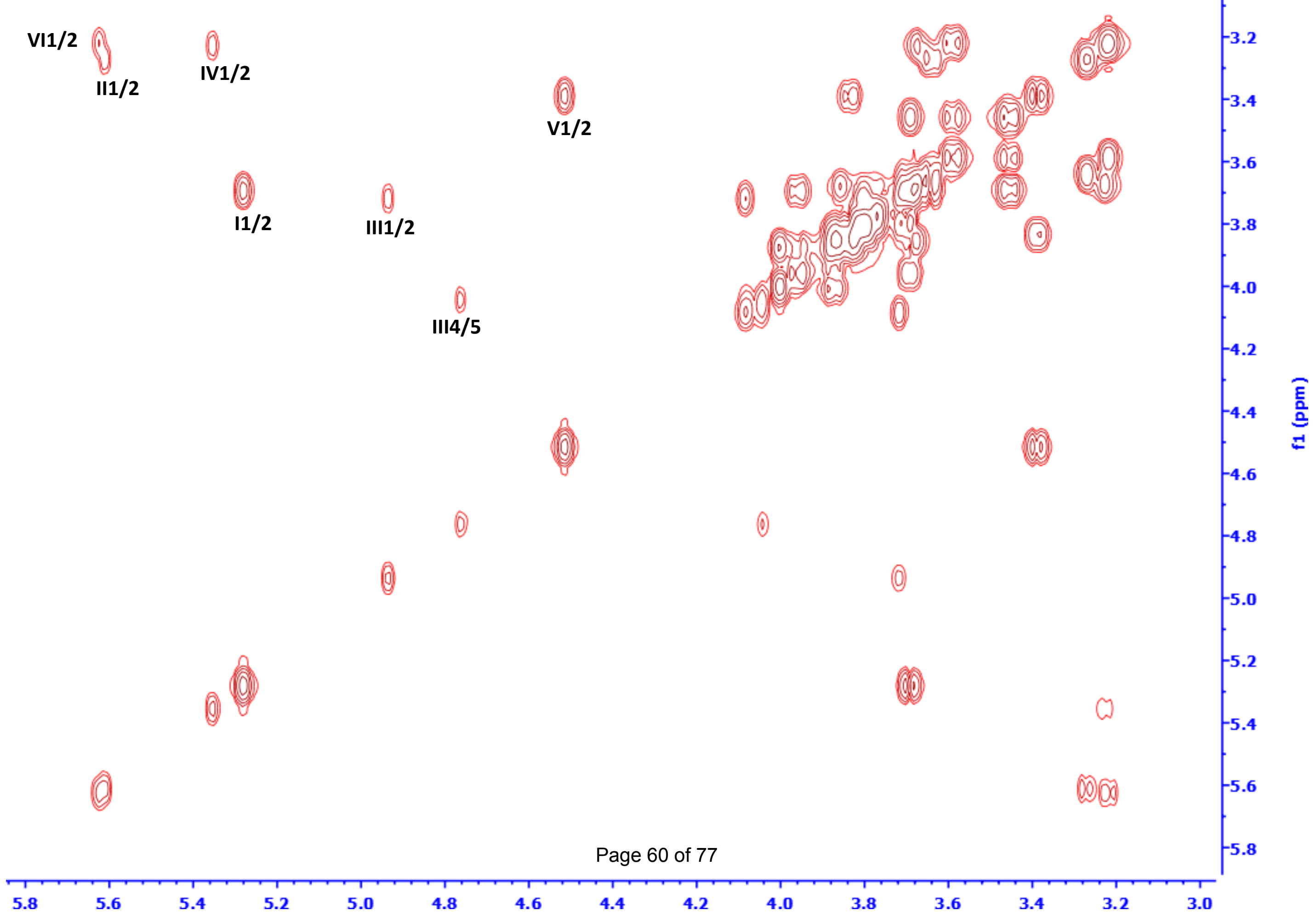

Supplementary Fig 42. 2D ^{1}H-^{13}C HSQC-TOCSY NMR spectrum of **6**

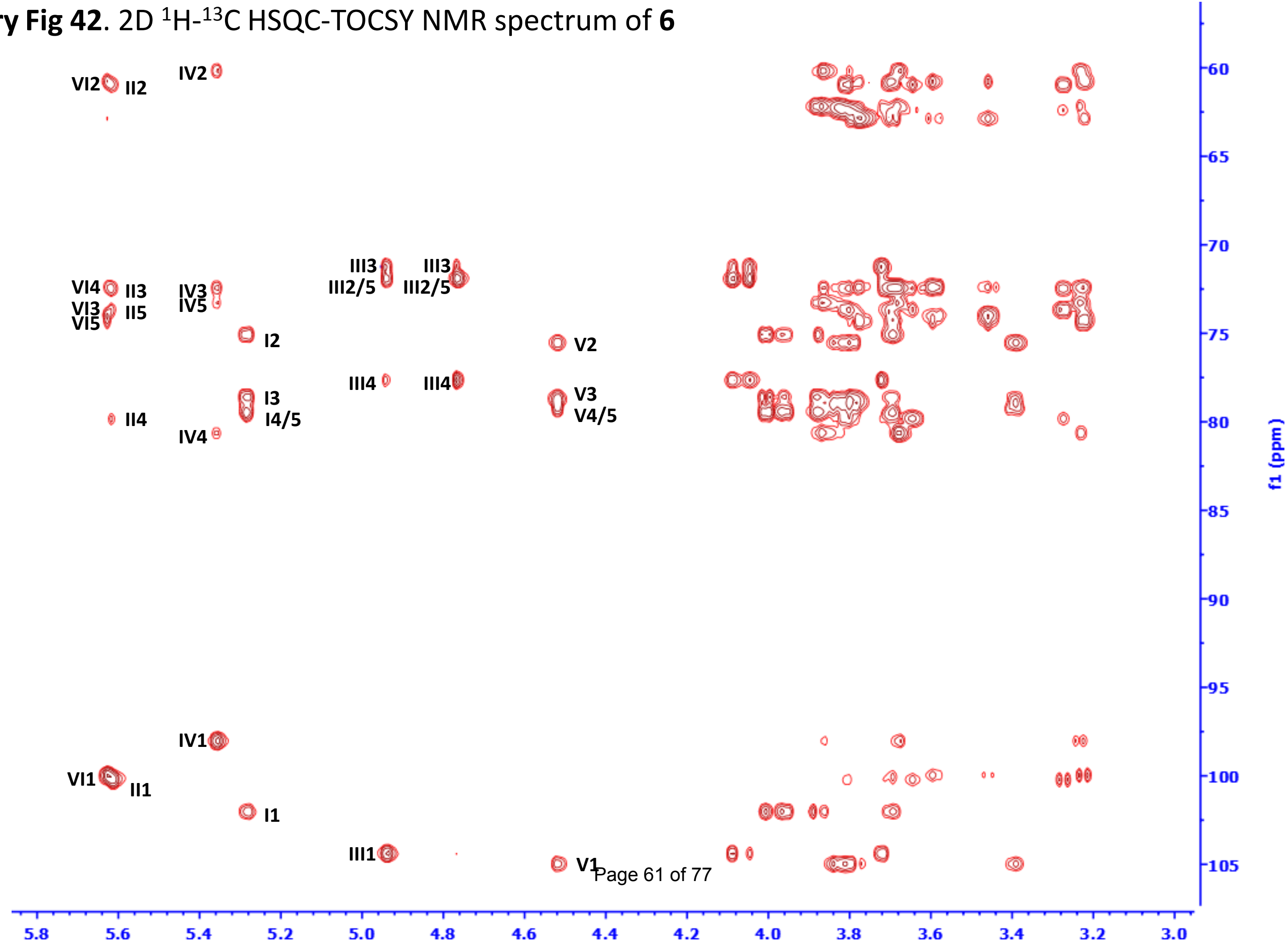

Supplementary Fig 43. 2D ^{1}H-^{13}C HMBC key correlations of **6**

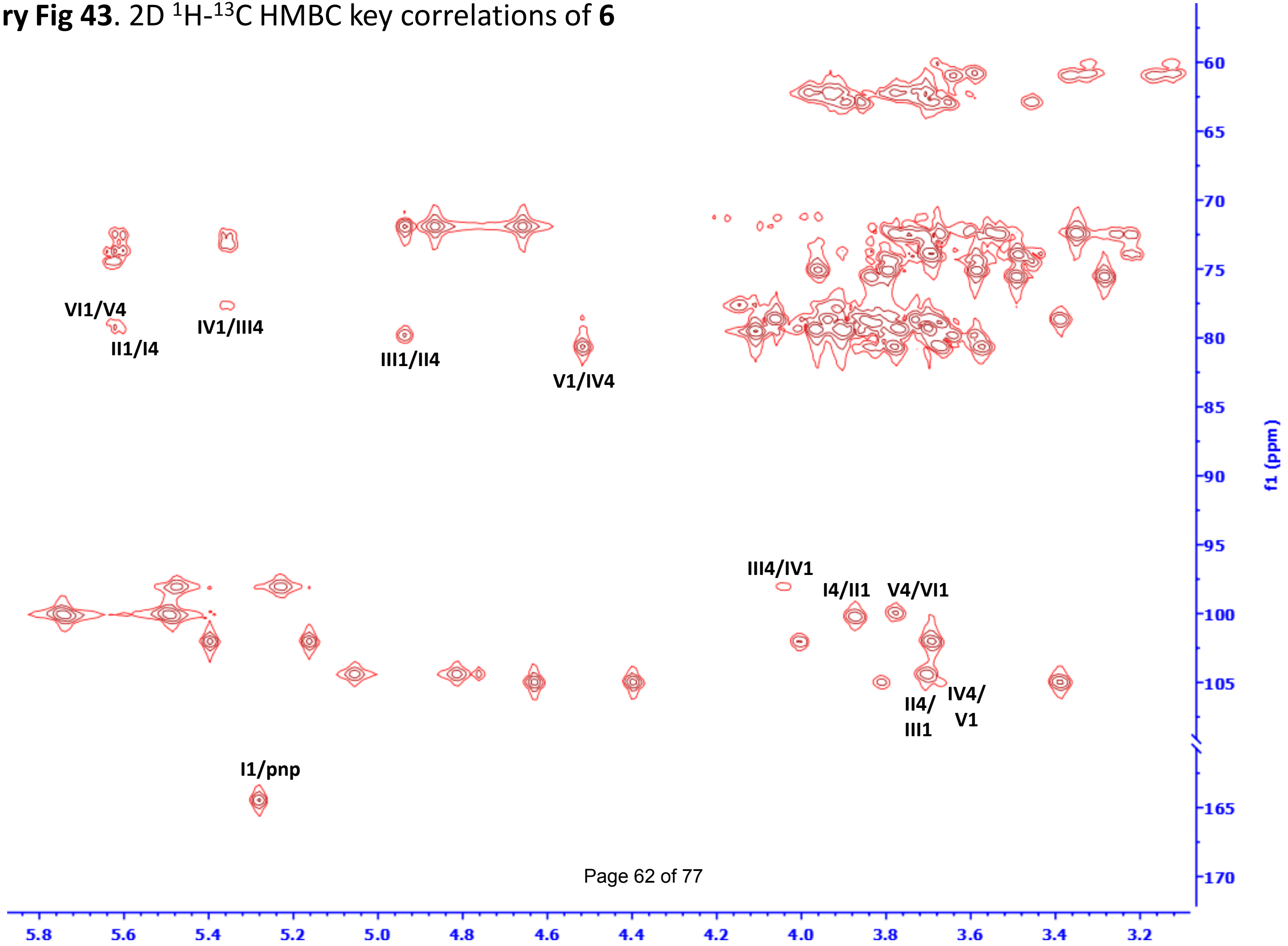

Supplementary Fig 44. 2D ^{1}H-^{1}H NOESY key correlations of **6**

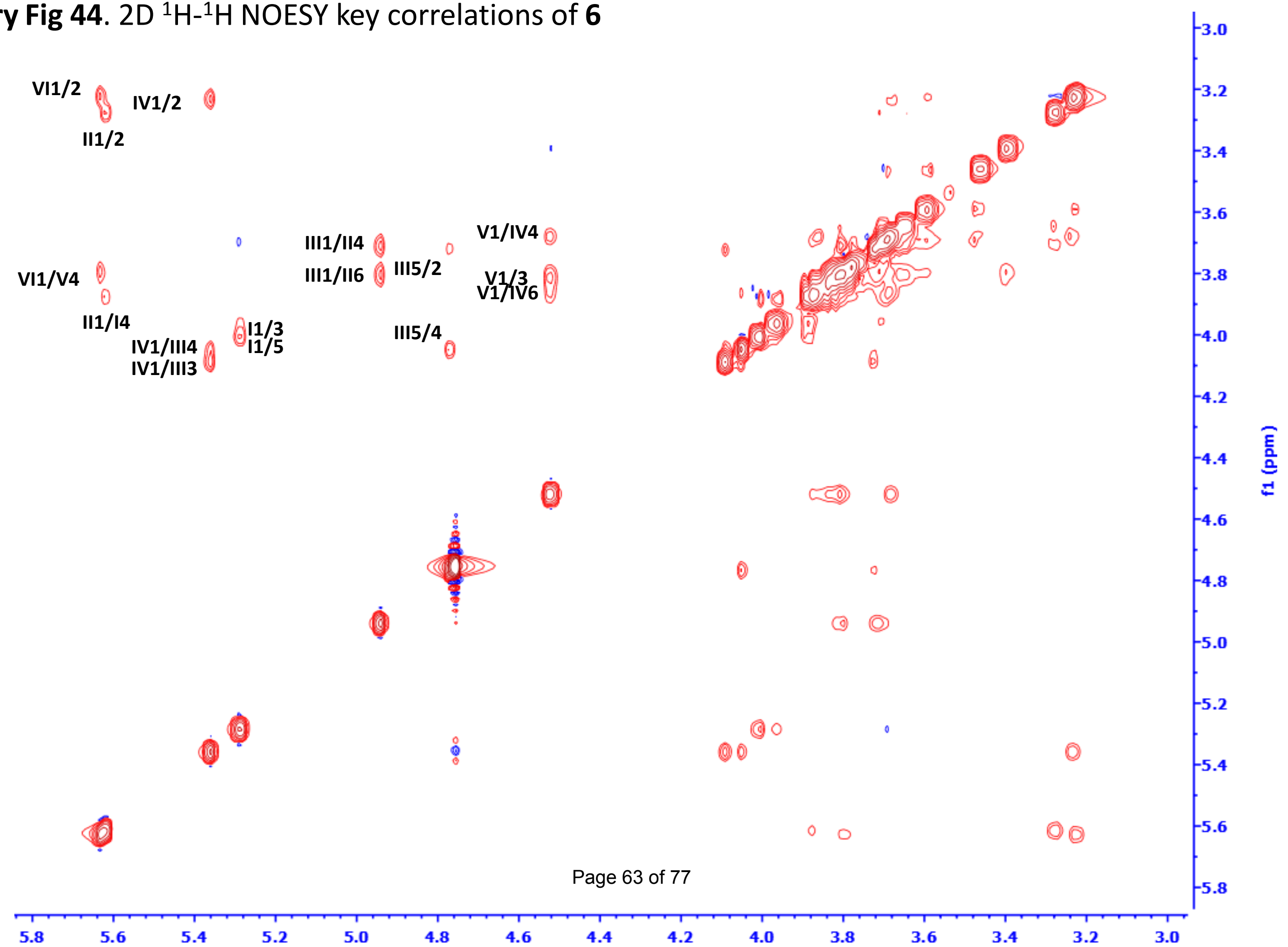

Supplementary Fig 45. ^{1}H-NMR spectrum of **7**

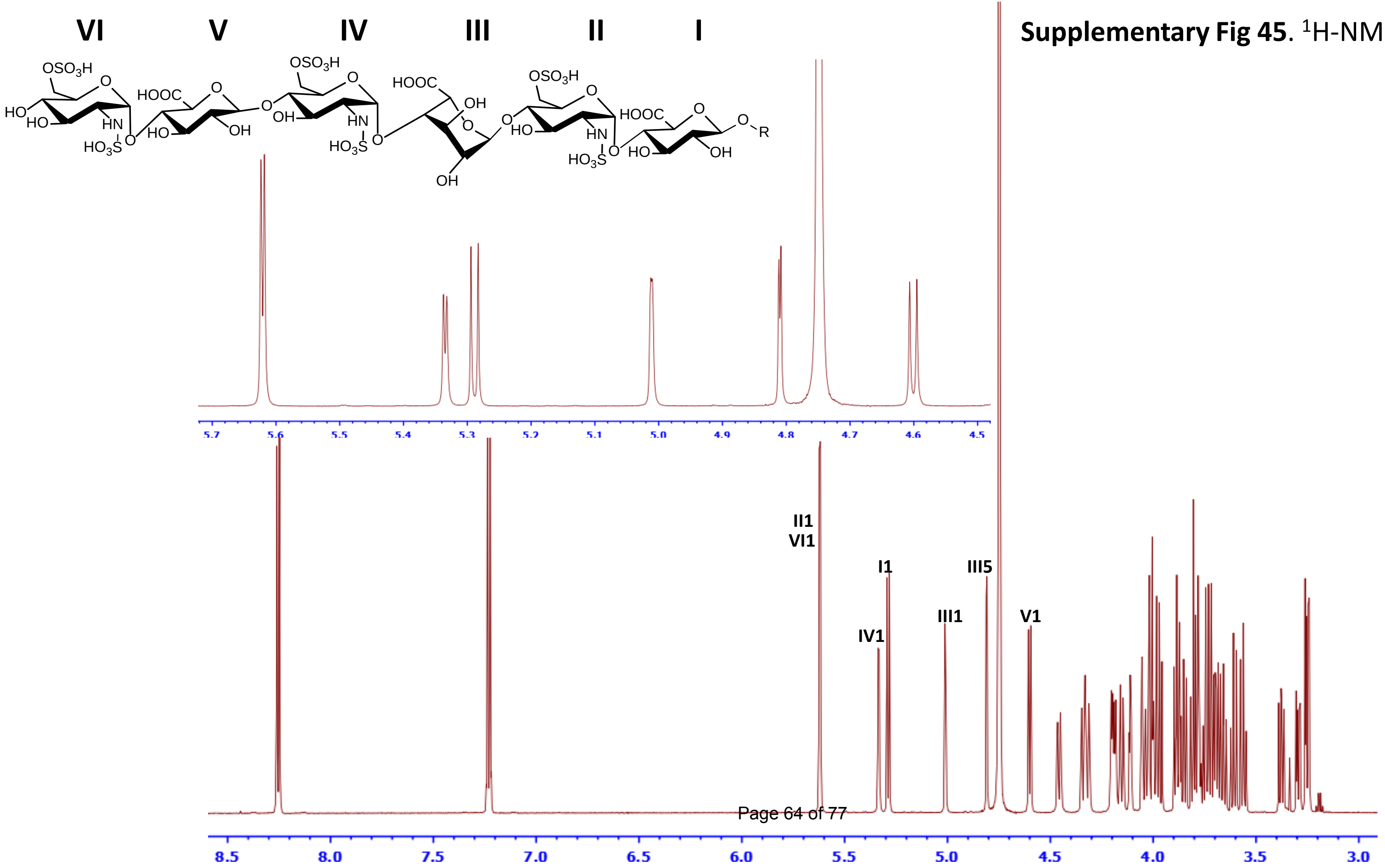

Supplementary Fig 46. ^{13}C-NMR spectrum of **7**

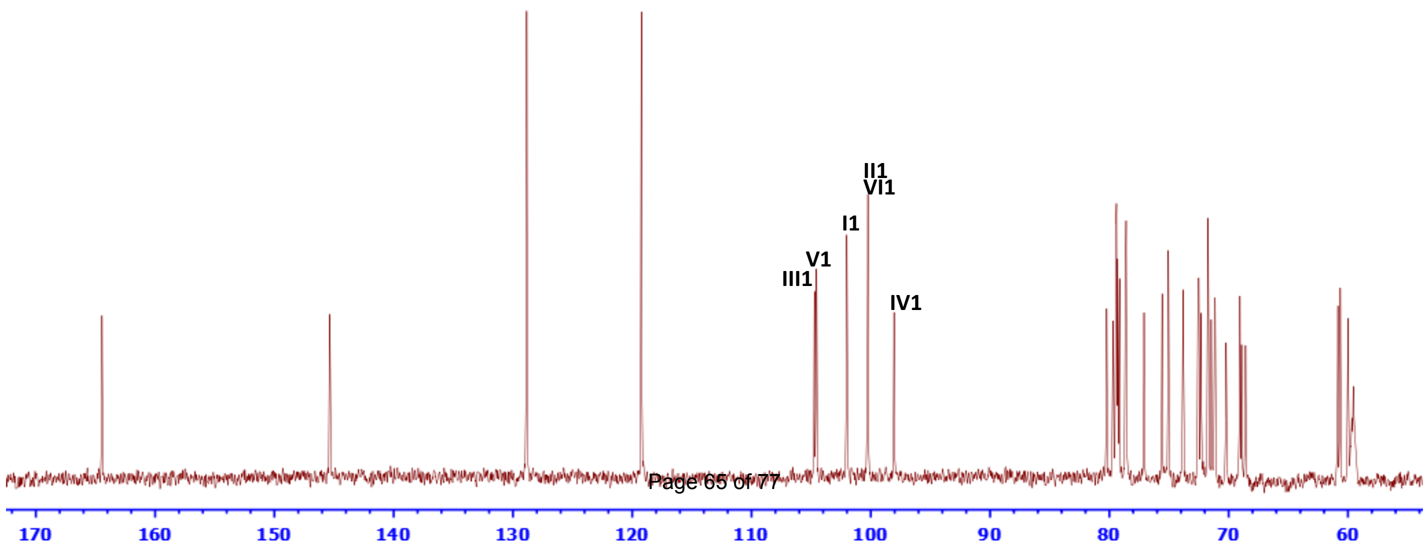

Supplementary Fig 47. 2D ^{1}H-^{13}C HSQC NMR spectrum of **7**

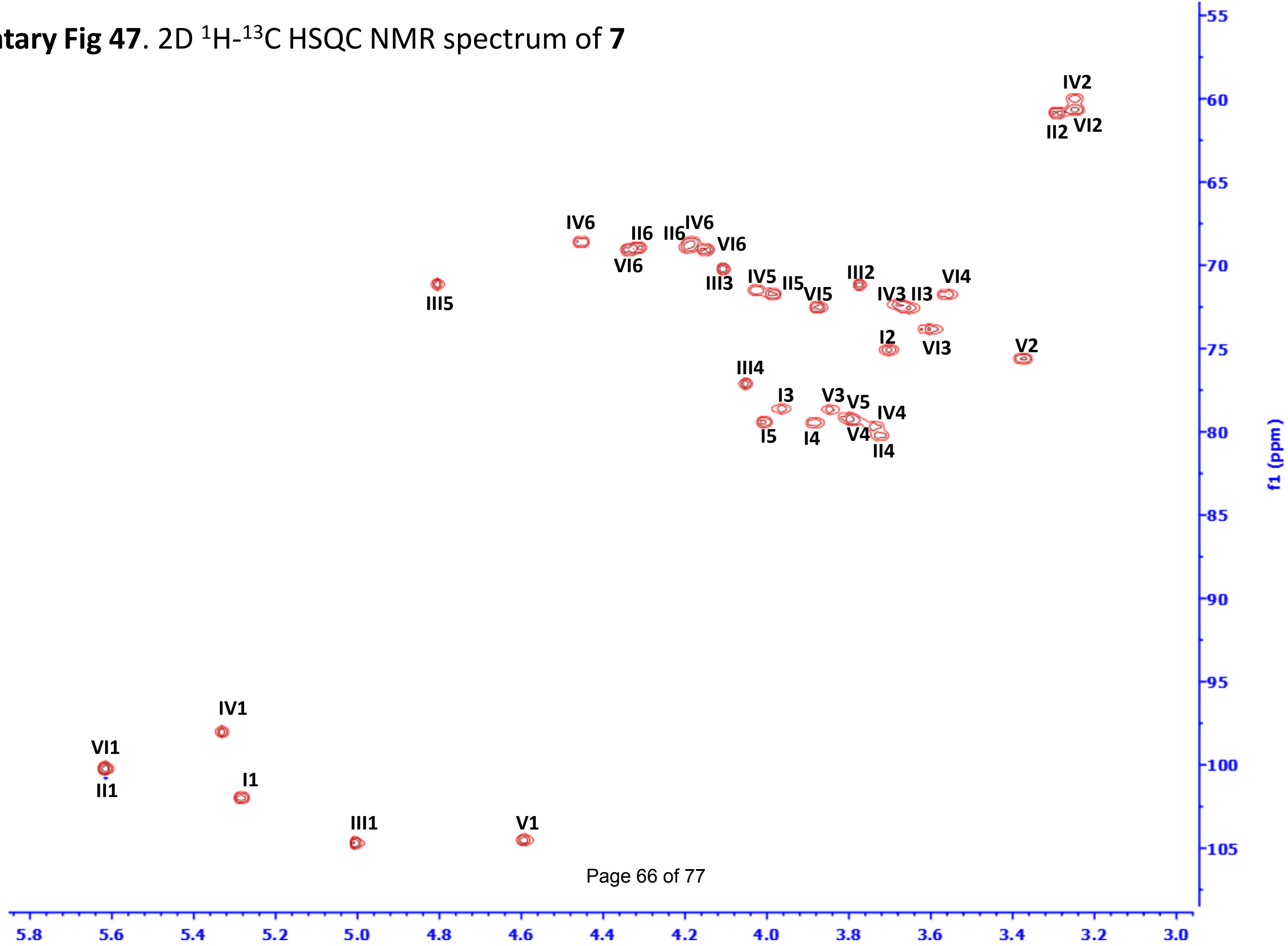

Supplementary Fig 48. 2D ^{1}H-^{1}H COSY NMR spectrum of **7**

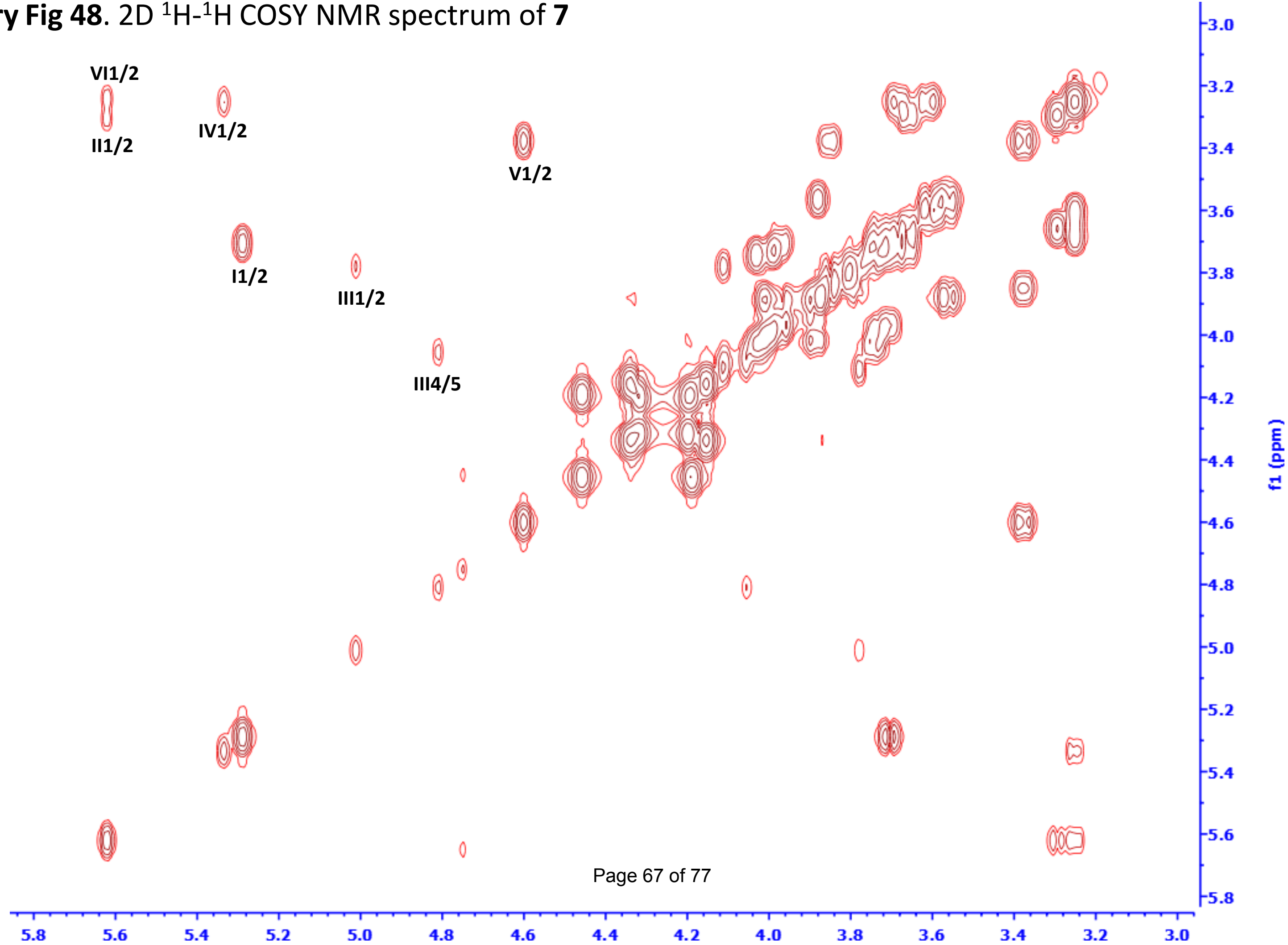

Supplementary Fig 49. 2D ^{1}H-^{13}C HSQC-TOCSY NMR spectrum of **7**

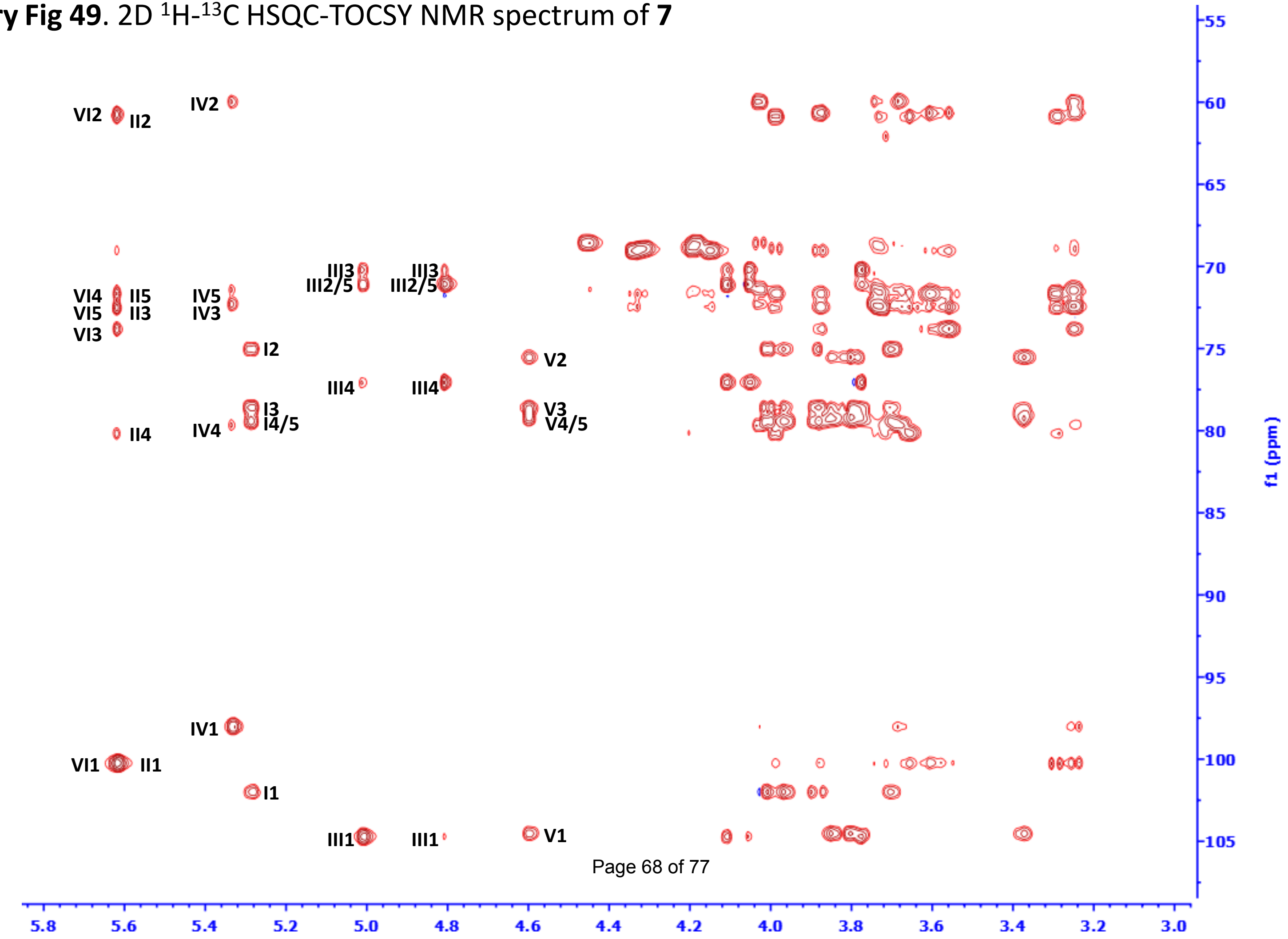

Supplementary Fig 50. 2D ^{1}H-^{13}C HMBC key correlations of **7**

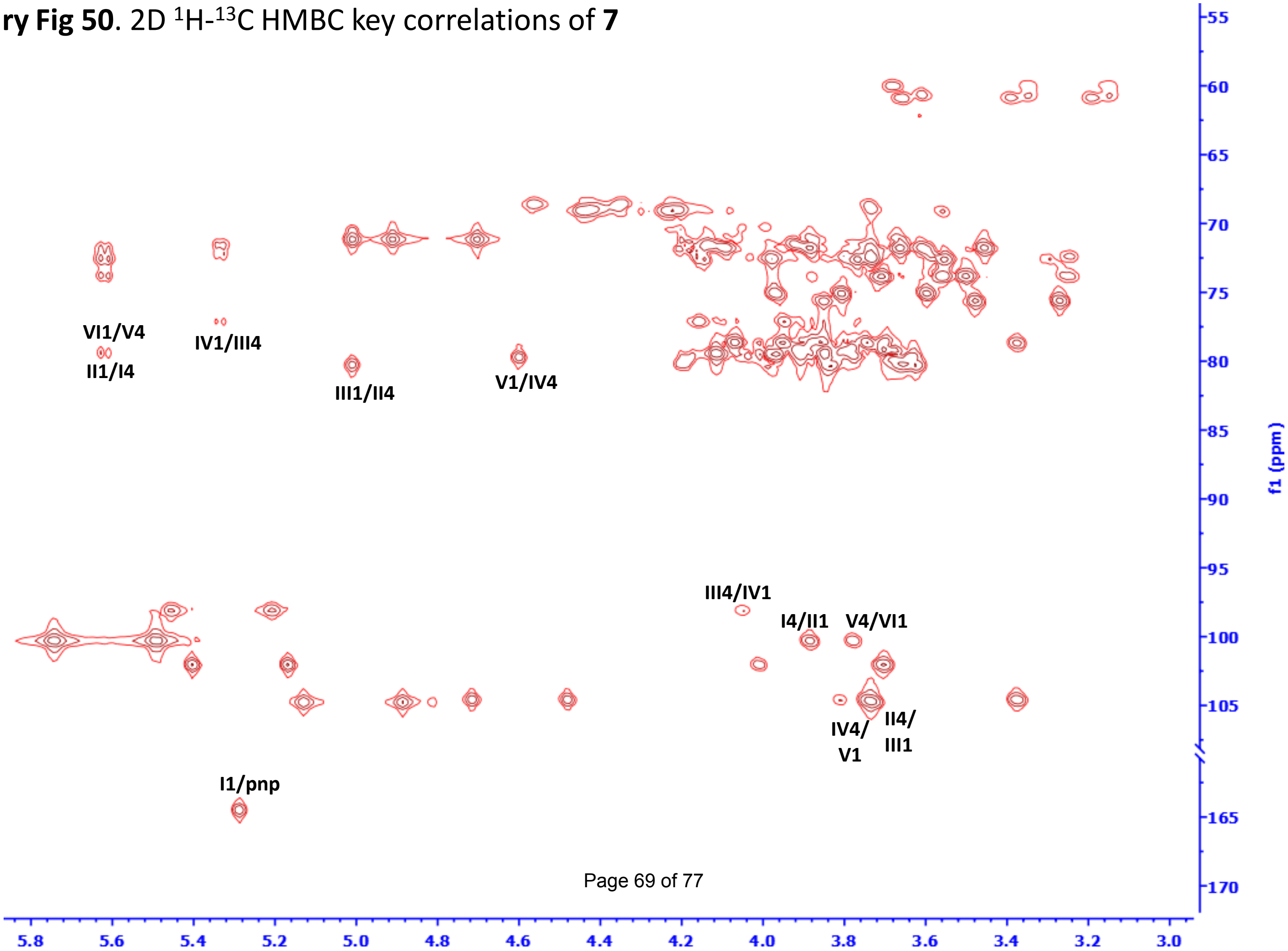

Supplementary Fig 51. 2D ^1H-^1H NOESY key correlations of **7**

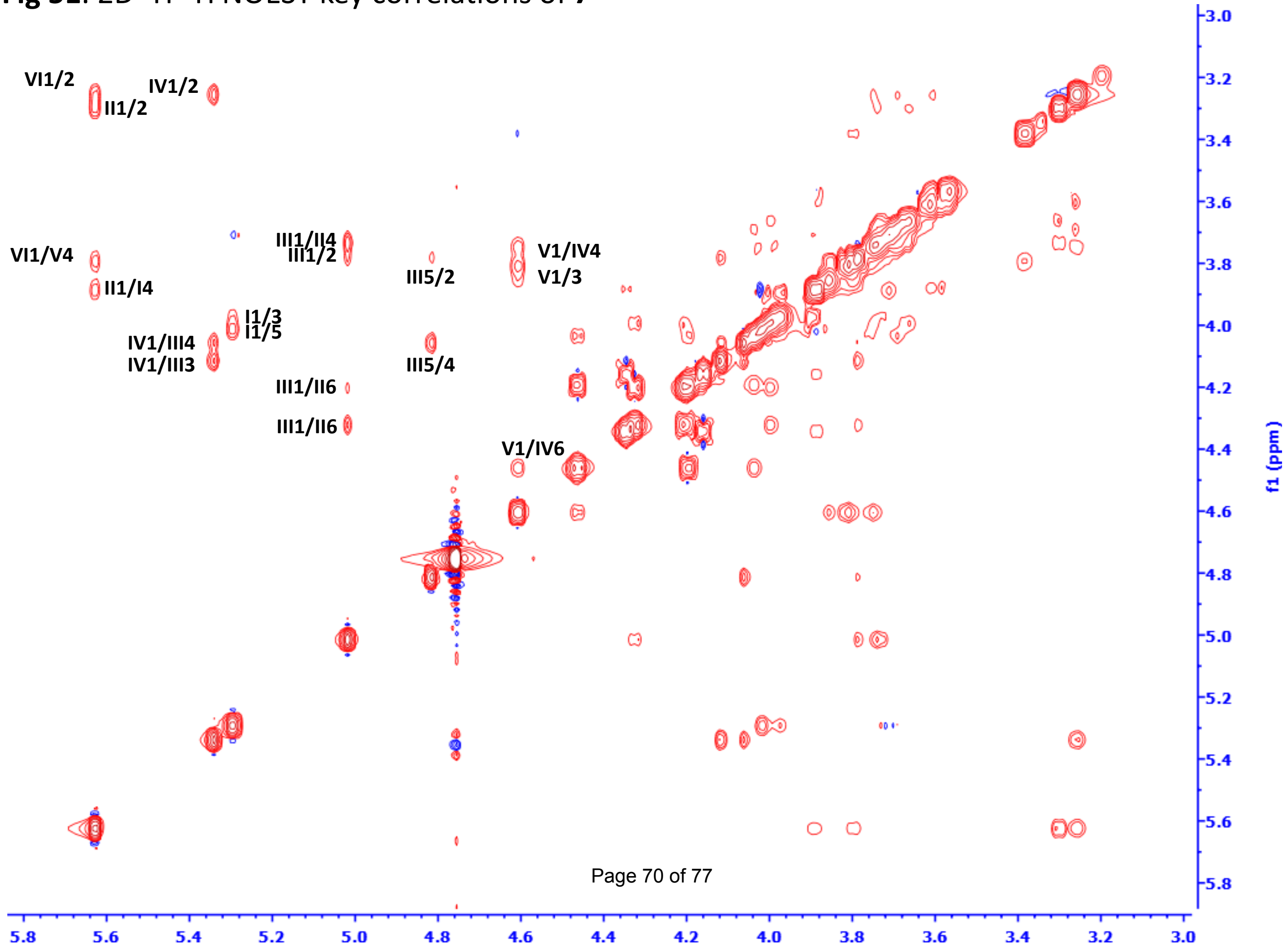

Supplementary Fig 52. ^{1}H-NMR spectrum of **8**

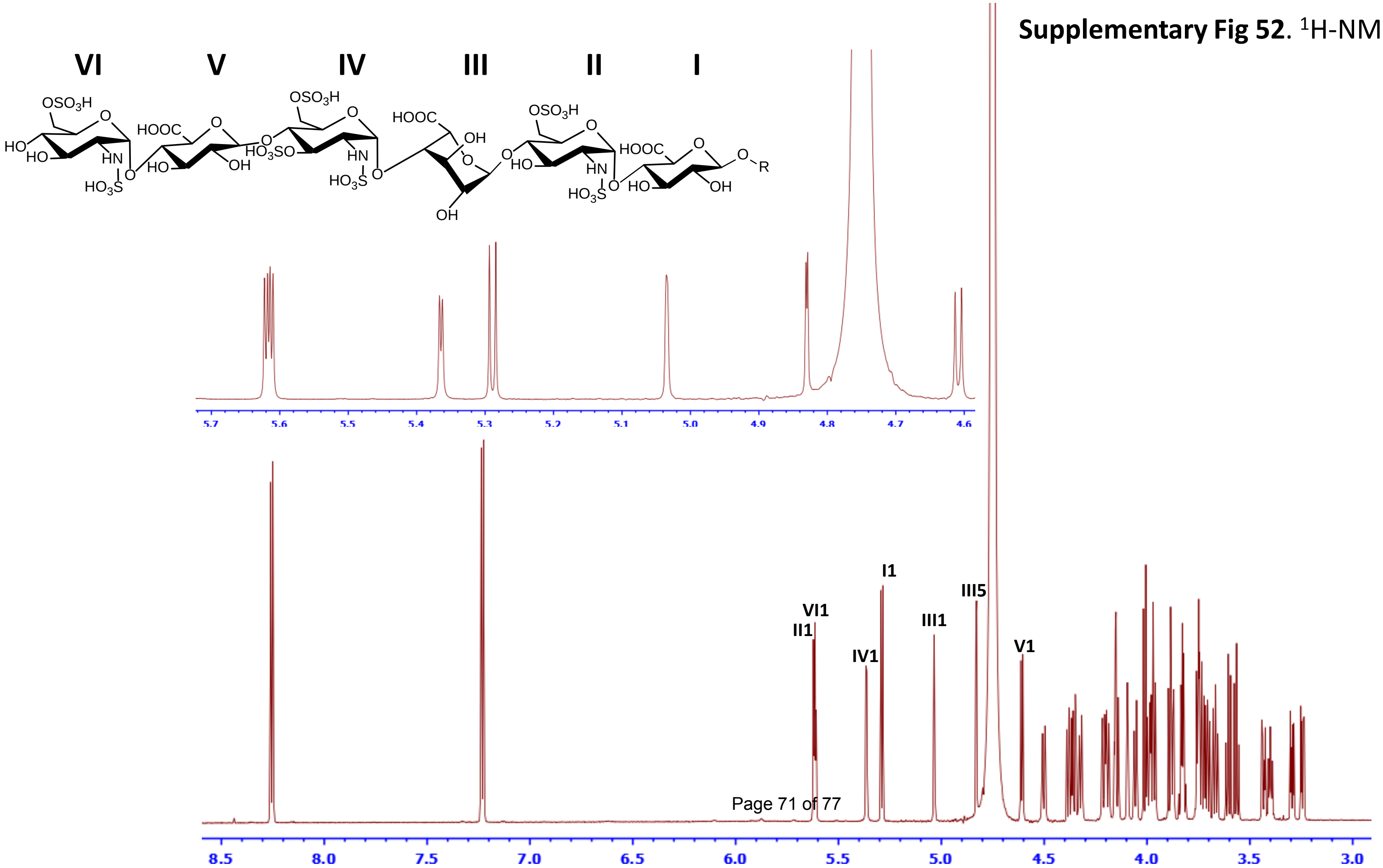

Supplementary Fig 53. ^{13}C-NMR spectrum of **8**

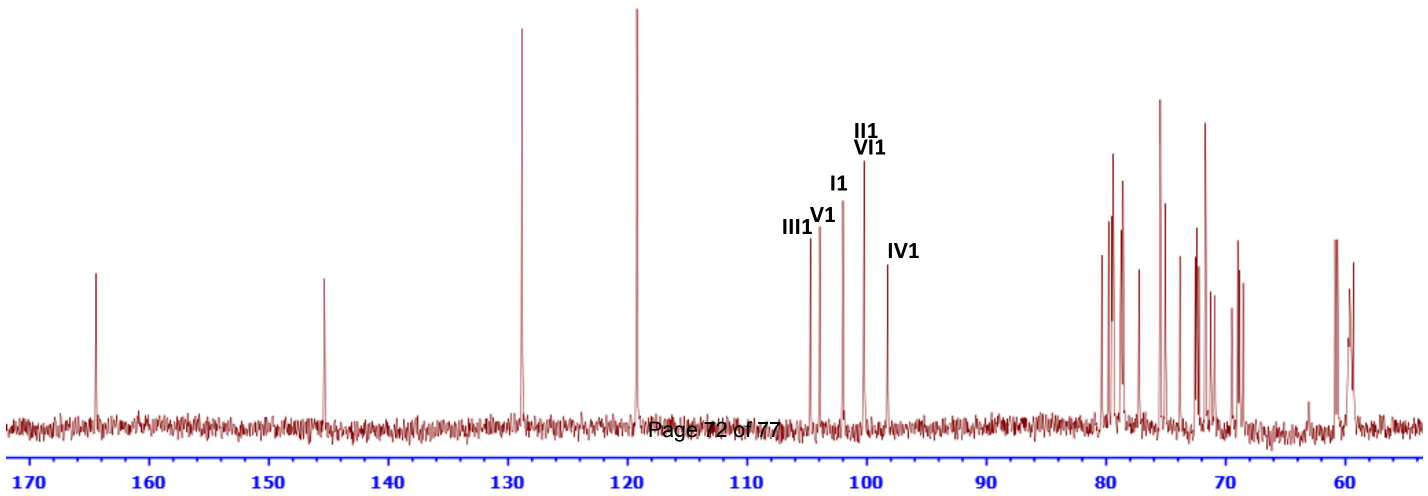

Supplementary Fig 54. 2D ^{1}H-^{13}C HSQC NMR spectrum of **8**

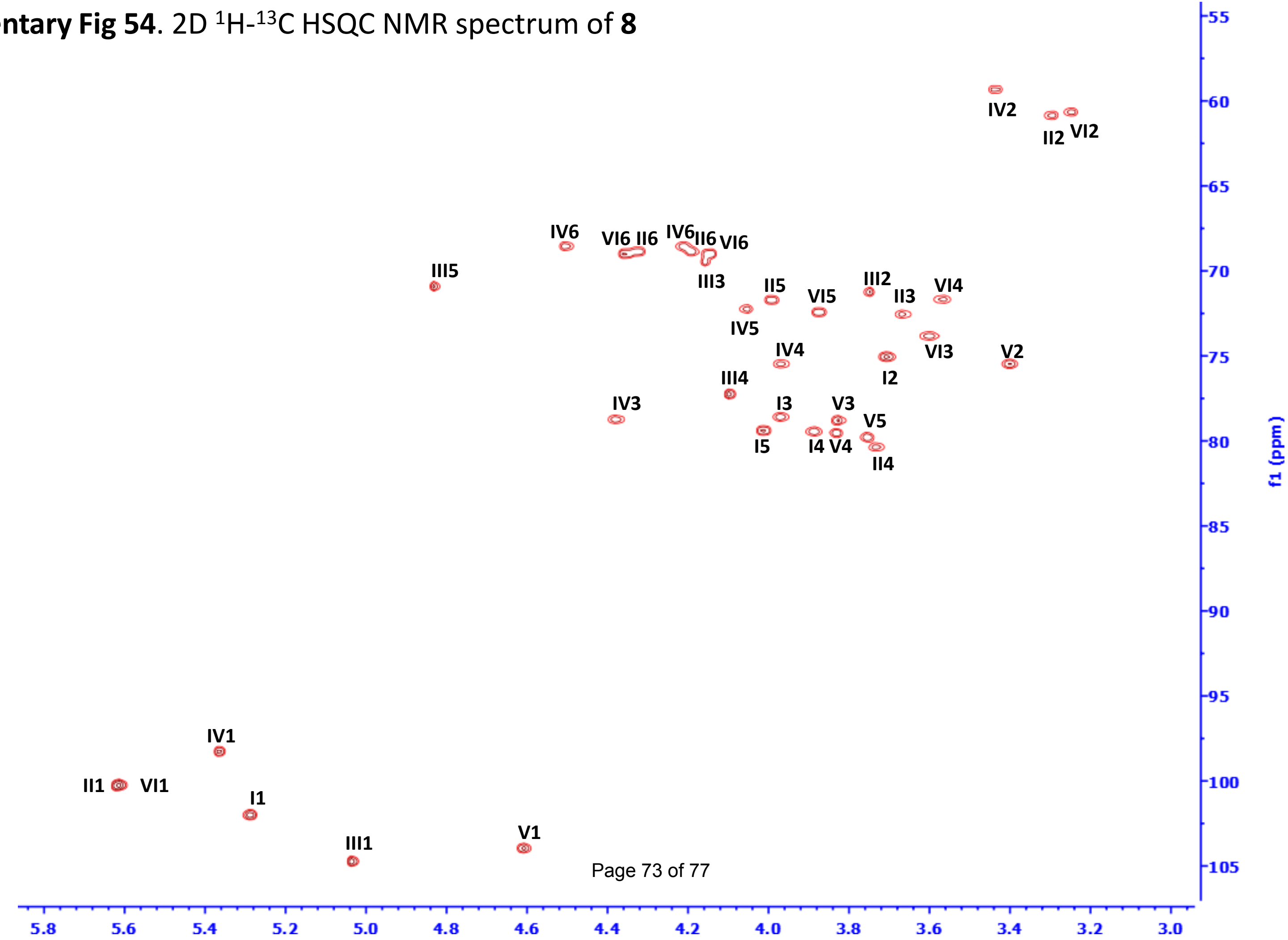

Supplementary Fig 55. 2D ^1H-^1H COSY NMR spectrum of **8**

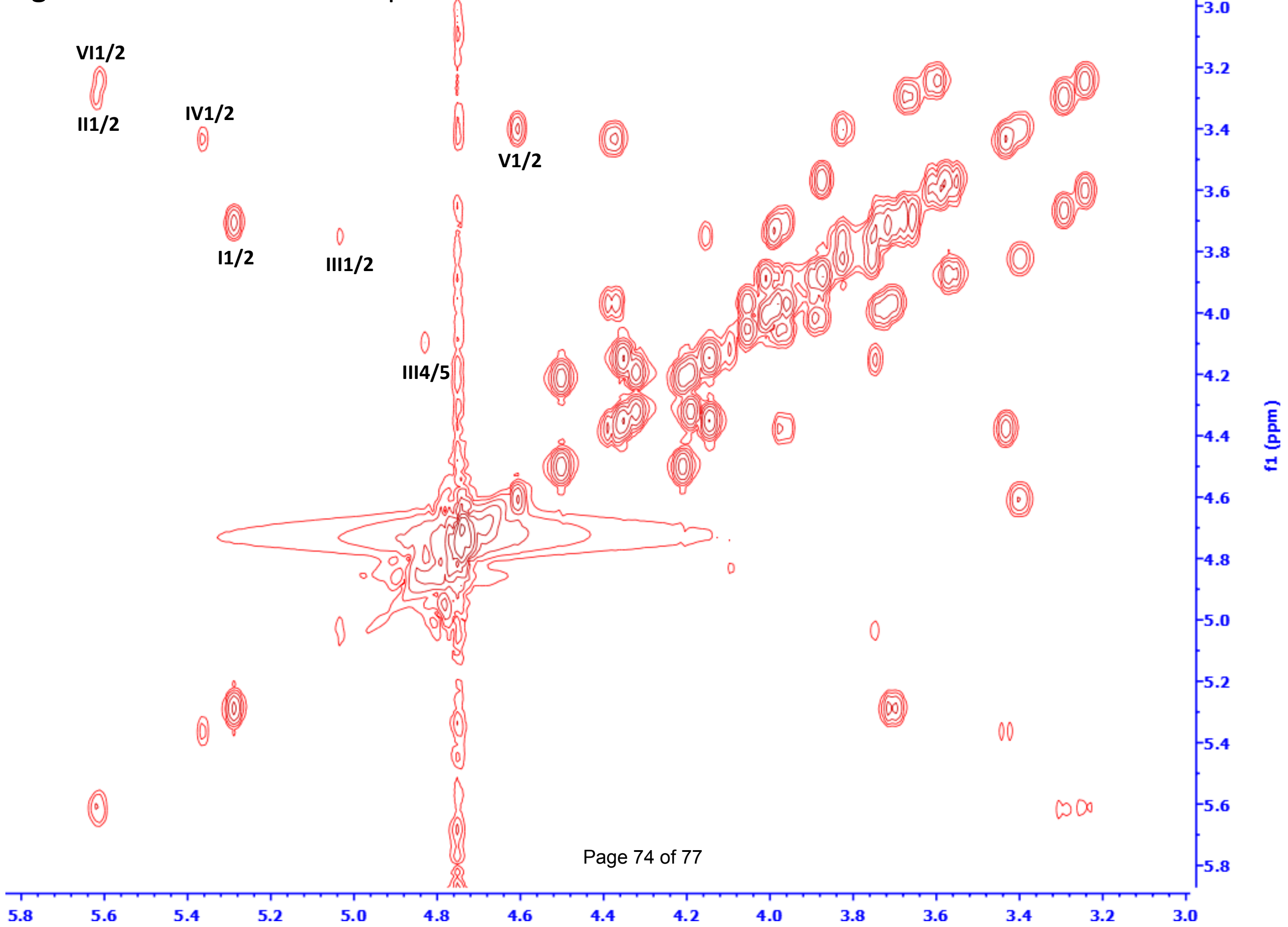

Supplementary Fig 56. 2D ^{1}H-^{13}C HSQC-TOCSY NMR spectrum of **8**

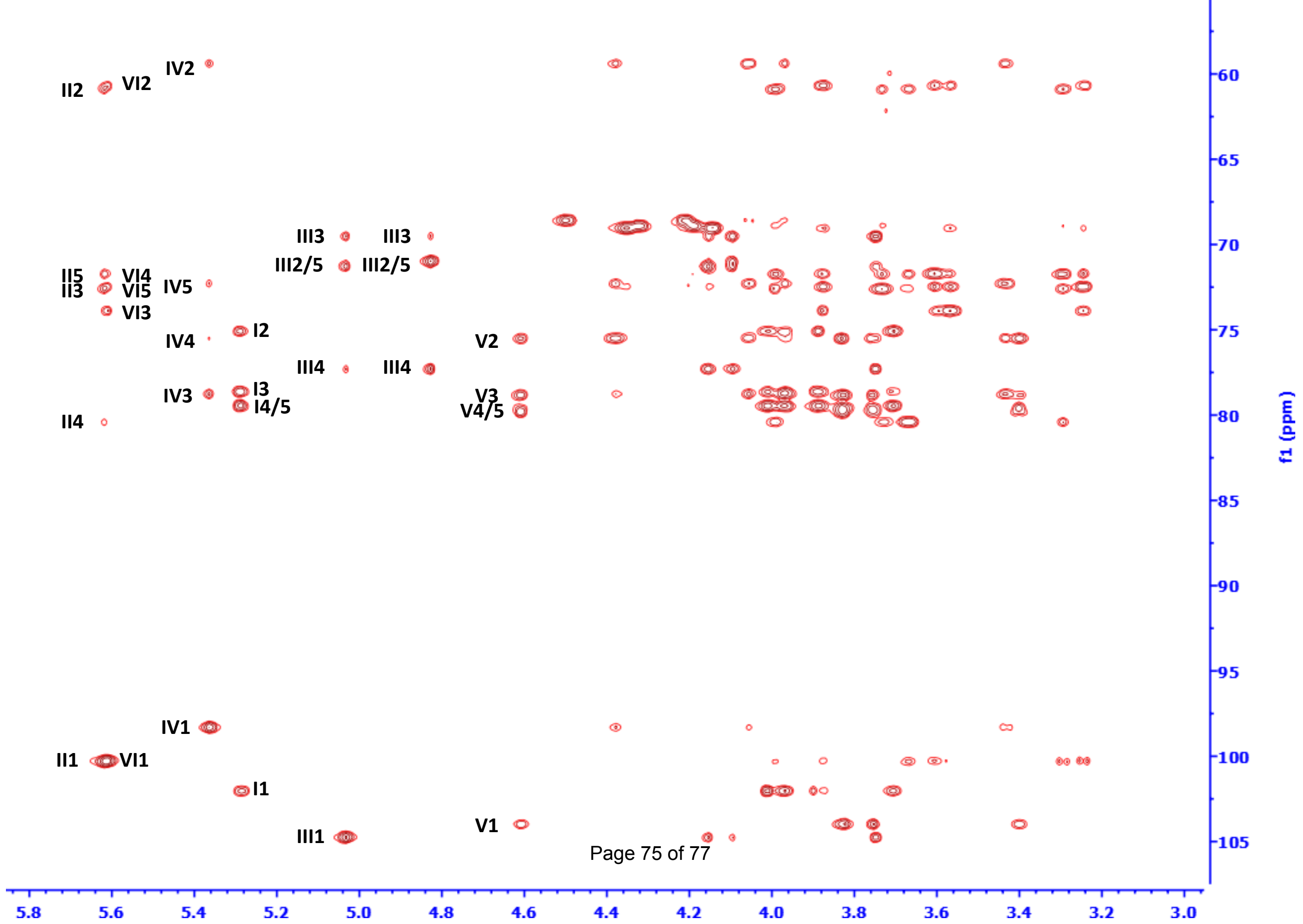

Supplementary Fig 57. 2D ^{1}H-^{13}C HMBC key correlations of **8**

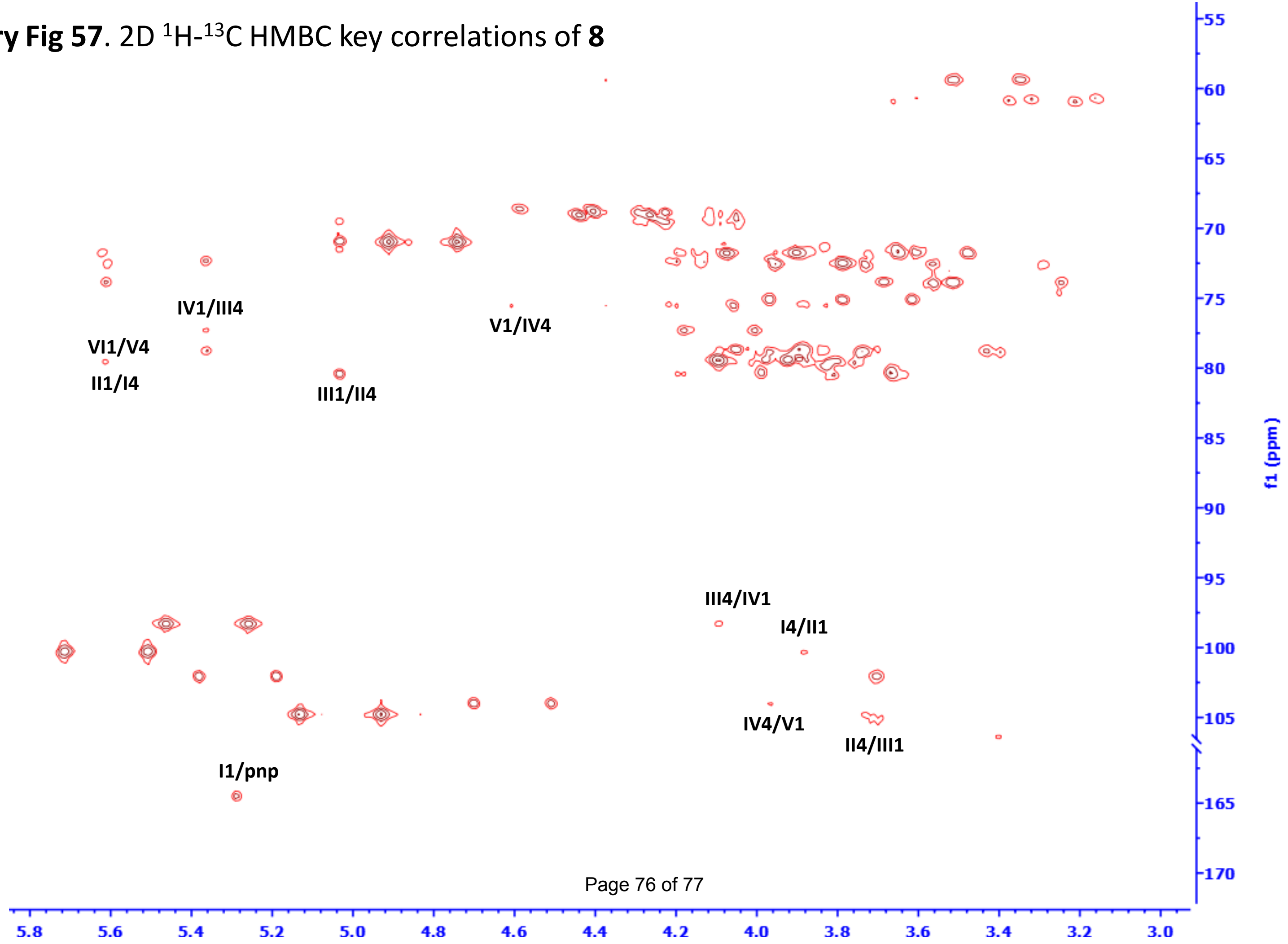

Supplementary Fig 58. 2D ^1H-^1H NOESY key correlations of **8**

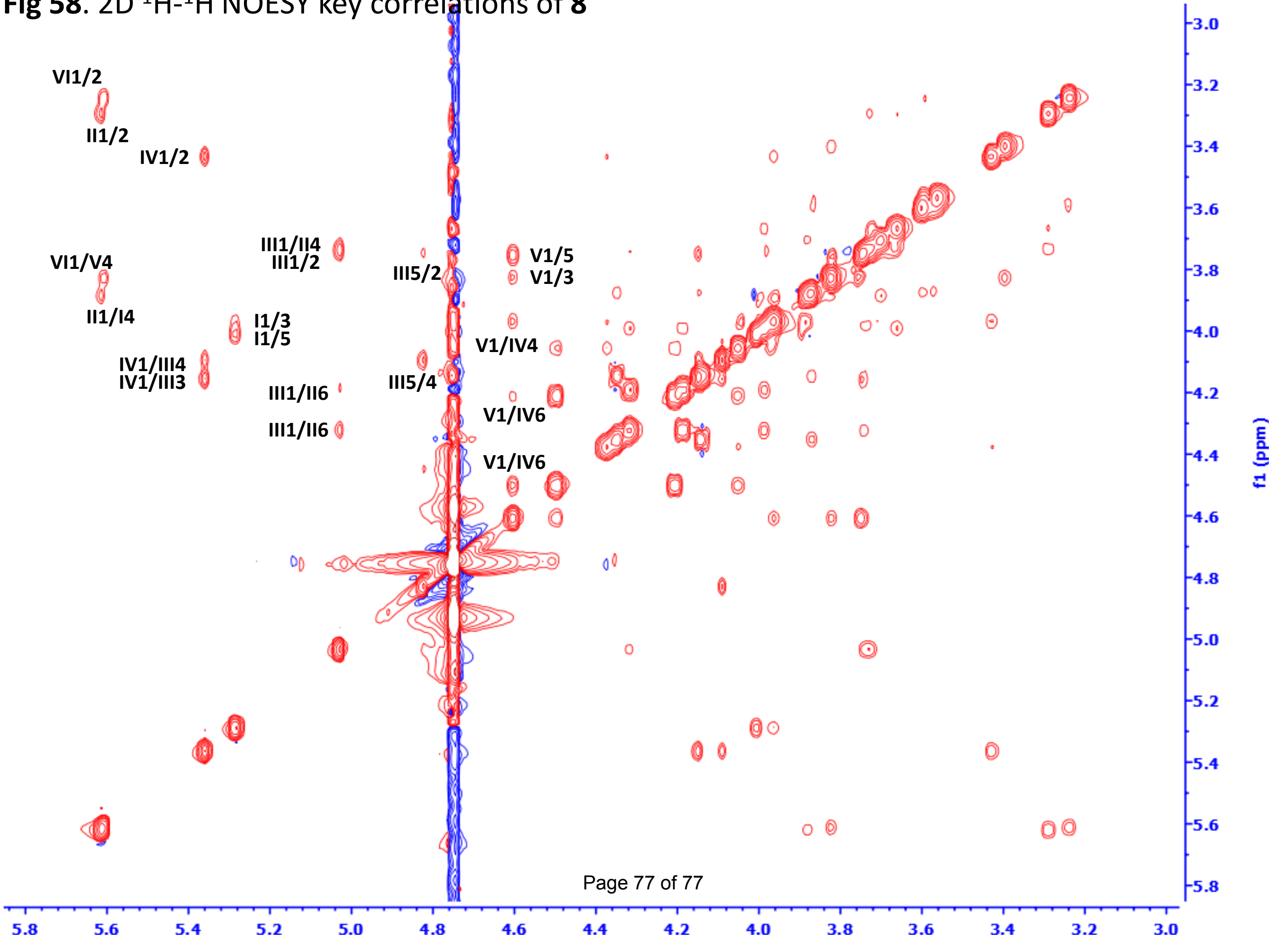

Supplement: Supplementary Information [file srep29602-s1.pdf]
